# Epigenetic anticipation for food and reproduction

**DOI:** 10.1093/eep/dvz026

**Published:** 2020-01-30

**Authors:** Christelle Leung, Bernard Angers, Patrick Bergeron

**Affiliations:** 1 Department of Biological Sciences, Bishop’s University, Sherbrooke, QC, J1M 1Z7, Canada; 2 Department of Biological Sciences, Université de Montréal, Montréal, QC, H3C 3J7, Canada

**Keywords:** anticipation, epigenetics, environmental predictability, phenotypic plasticity, resources fluctuations, reproductive behavior, *Tamias striatus*

## Abstract

Physiological changes in anticipation of cyclic environmental events are common for the persistence of populations in fluctuating environments (e.g. seasons). However, dealing with sporadic resources such as the intermittent production of seed masting trees may be challenging unless reliable cues also make them predictable. To be adaptive, the anticipation of such episodic events would have to trigger the corresponding physiological response. Epigenetic modifications could result in such physiological anticipatory responses to future changes. The eastern chipmunk (*Tamias striatus*) is known to adjust its reproductive activity to match juvenile weaning with peak seed availability of masting trees, which are essential for their survival. We therefore expected that epigenetic changes would be linked to spring reproductive initiation in anticipation for beech seed availability in fall. We correlated the variation of DNA methylation profiles of 114 adult chipmunks captured in May with beech seeds abundance in September, over 4 years, for three distinct populations, as well as individuals sampled twice during reproductive and non-reproductive years. The significant correlation between spring epigenetic variation and the amount of food in the fall confirmed the phenotypic flexibility of individuals according to environmental fluctuations. Altogether, these results underlined the key role of epigenetic processes in anticipatory responses enabling organisms to persist in fluctuating environments.

## Introduction

Immediate response to environmental changes can provide advantages to ensure the survival of organisms, as exemplified by homeostasis [e.g. 1, 2]. However, the benefit of a response to environmental changes does not necessarily need to be immediate, but it could be in expectation of a future environment. For instance, seasonal migration, accumulation of food reserves, hibernation or color changes are spectacular phenotypic modifications in anticipation for winter. Pre-programmed phenotypes could thus evolve in response to regular, consistent and predictable environmental changes such as the one observed on a daily [e.g. circadian rhythm; 3, 4] or seasonal bases [e.g. obligatory migration of birds during winter; 5]. However, environmental changes could also be episodic events; occurring occasionally and at irregular intervals. In such cases, organisms must rely on specific environmental signal interpreted as reliable announcement of future changes.

The capacity to undergo phenotypic changes in prevision to contrasted future environmental conditions is similar to the predictive adaptive response hypothesis described in developmental biology [6]. This form of developmental plasticity is based on the fact that cues received in early life influence the development of a phenotype that will match the environmental condition encountered later in life [6–8]. For instance, the amount of maternal melatonin secretion for the meadow vole (*Microtus pennsylvanicus*) is used as a cue predicting postnatal photoperiod (i.e. either the offspring would born in autumn or spring), and alter the development of different coat thickness by the offspring, in prevision of the cold or warm temperature when they would leave the nest, weeks later [9, 10].

Such adaptive response in anticipation of a future environmental condition could underline phenotypic flexibility and, thus, be advantageous in episodic events. In pulsed-resource systems characterized by intermittent periods of food abundance and periods of resources paucity [11], juvenile conception is typically triggered prior to food availability, in order to match future juvenile needs with resource availability and ensure recruitment [12–14]. This indicates that organisms can perceive particular environmental signal interpreted as reliable announcement of future changes and, in this case, plastically adjust their reproductive behavior in anticipation of food abundance.

Interactions between environmental cues and epigenetic changes can result in phenotypic plasticity associated with anticipatory responses without genomic changes [15–17]. Epigenetic processes consist of enzyme-mediated chemical modifications of histones, DNA and RNA. These changes modify the properties of these molecules, for instance by altering gene expression, while the amino/nucleic acids sequence remained unchanged [17–19]. Interestingly, epigenetic changes might result from several sources, including environmental conditions [17, 20, 21]. Thus, environmentally induced epigenetic variation represents an important mechanism in adjusting individuals’ phenotype in response to environmental fluctuations, being one of the molecular mechanisms underlying phenotypic plasticity [15, 16, 18].

This study aims at testing the hypothesis that epigenetic changes represent one of the first step following the perception of an environmental signal in an anticipatory response, by correlating early epigenetic states (i.e. epigenetic states before the realized and observable phenotype) to environmental episodic events. To address this objective, we used a pulsed-resource system where anticipatory response is expected to synchronize individuals’ reproduction with resource availability and avoids the production of juveniles in dearth periods. Reproduction of eastern chipmunks (*Tamias striatus*) is tightly synchronized with the availability of American beech (*Fagus grandifolia*) seeds in the northeastern part of North America [14]. No juveniles emerged from burrows during the falls associated with low beech seed abundance, whereas reproduction occurred during the summer (June) of years with high beech seed production that coincides with juvenile emergence in the fall (September). Seeds production of American beech is occurring occasionally and at irregular intervals [22], where periods of resource abundance are defined as mast events [11]. To synchronize reproduction with the episodic nature of masting events among years, the environmental cue responsible for the plasticity of reproductive behavior in chipmunks is expected to occur during the same year, early in the spring. Epigenetic modifications in adults was therefore expected prior to reproduction period during the spring, in anticipation of resource availability in the fall.

We focused on DNA methylation as epigenetic markers. We compared DNA methylation differences of individuals sampled early in the spring, prior to the summer reproductive season, for years presenting different quantity of food availability in the following fall. Because the genotype, as well as environmental factors, influence individuals’ epigenetics [20, 21], we additionally computed different pairs of comparisons to control for the accuracy of the selected epigenetic markers. Such comparisons involved three distinct chipmunk populations [23, 24]. We also compared epigenetic differences between juveniles and adults. Our prediction is to detect a correlation between epigenetic variation in the spring and food availability the following fall, independently from genetics and spatial environmental factors.

## Results

### Beech Seeds and Juveniles

**Figure 1:**
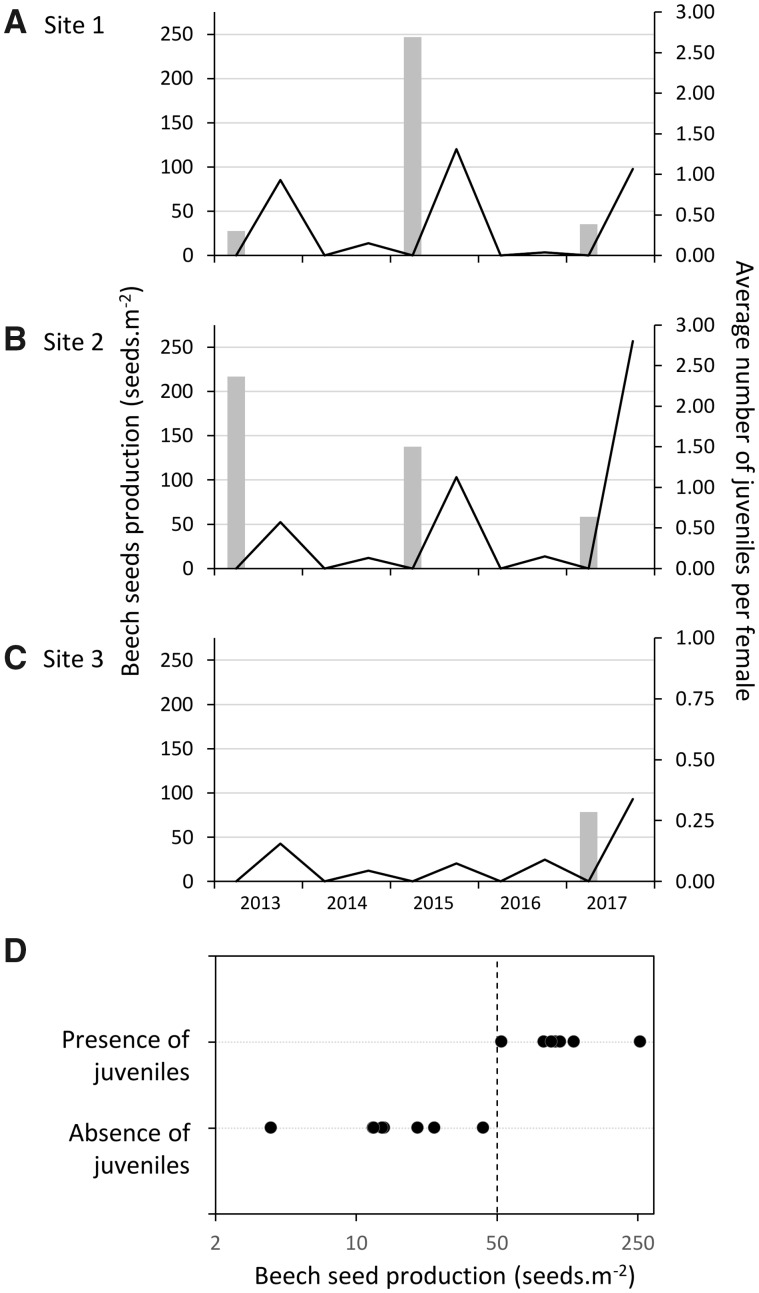
eastern chipmunk’s reproduction as a function of beech seeds production. Number of beech seeds produced per m^2^ (lines, left axis) and average number of juveniles per female resulting from summer reproduction (bars, right axis) among the three sampled sites (**A–C**). (**D**) Fall sampling of juveniles first captured as a function of beech seeds production

Beech seed production strongly varied according to sampling year and, to a lesser extent, among sites (Fig. 1). Sites 1 and 2 were very similar: we observed peaks of beech seeds production during the falls of 2013, 2015 and 2017, while 2014 and 2016 displayed the lowest seed production on average. However, site 3 always displayed a low seed production except in 2017 (Fig. 1). As expected, the presence of juveniles resulting from summer reproduction was associated with years of high beech seeds production, with a threshold of ∼50 seeds per m^2^. For subsequent analyses, we categorized sampling years, for each site, as reproductive or non-reproductive periods based on beech seed quantity with the threshold of 50 seeds per m^2^ in the fall and presence of juveniles resulting from summer reproduction (Fig. 1D).

### MSAP Analysis

**Figure 2:**
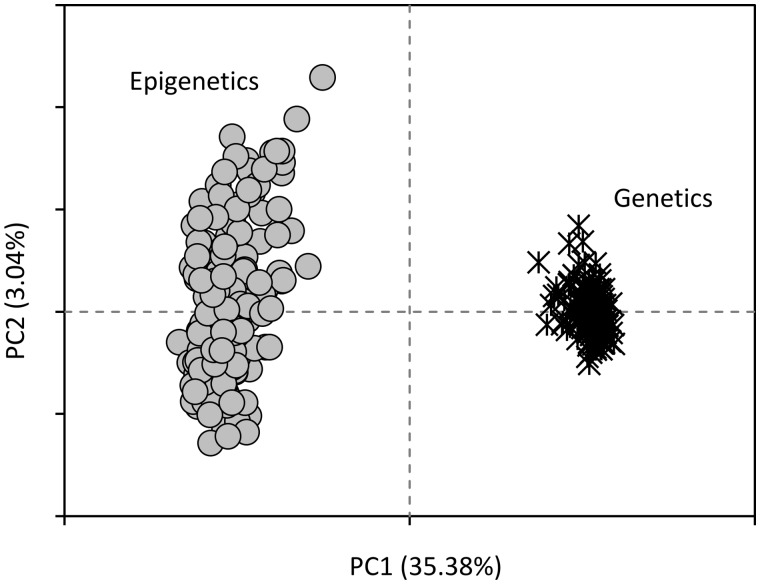
eastern chipmunks genetic and epigenetic variation. PCA with 199 loci generated by MS-AFLP

For the *methyl-sensitive amplification polymorphisms* (MSAP) analysis, a locus is defined by a fragment of a given size amplified in at least one individual. MSAP analysis performed on 114 adults and 48 juveniles provided a total of 208 loci. Only nine (4.33%) non-reproducible loci were observed and removed from the dataset. Within the remaining 199 reproducible loci (present in both MspI and HpaII treatments), 74 loci were genetically variable among individuals (MspI treatment) compared to 109 loci for epigenetic variation (HpaII treatment). Epigenetic variation was significantly higher than genetic variation (*F*_1, 370_ = 2021.2; *P *<* *0.001; Fig. 2).

### Partitioning of Genetic Variation

Despite genetic differentiation among sites, we did not detect consistent genetic differences among individuals according to their age, sex or sampling years (Table 1). Those results suggested that the three sampling sites represented distinct populations, and for a given site, individuals sampled during reproductive or non-reproductive years belonged to the same population.

**Table 1:** eastern chipmunks genetic and epigenetic variation

	Factors	*n*	Percentage of variation	
			Genetics	Epigenetics
Age	Adult	114	*NS*	1.27%***
	Juvenile	48		
Sex	Male	77	*NS*	*NS*
	Female	84		
Site	Site 1 (N 45°6′45″; W 72°25′58″)	68	9.55%***	2.16%***
	Site 2 (N 45°6′14″; W 72°26′20″)	60		
	Site 3 (N 45°7′50″; W 72°23′38″)	33		
Reproductive periods	Reproductive	79	*NS*	1.26%***
	Non-reproductive	82		
Beech seeds fall production			*NS*	0.93%*
Genetics				2.24%***

Marginal effect of different explanatory variables (Factors) on total genetic and epigenetic variation resulting from pRDA. Sample size (*n*) is given for each factor.

*NS*: non-significant.

* *P* < 0.05;

*** *P* < 0.001.

### Partitioning of Epigenetic Variation

Individuals’ age, genetic variation, sampling sites, reproductive years and quantity of beech seed produced in fall of each sampling year explained significantly (*P *<* *0.05) the measured epigenetic variation. However, we did not detect any epigenetic differences between males and females (Table 1). Because of the significant effect of genetics on epigenetic variation, individual genetic profiles were kept as conditioning variables in all subsequent epigenetic variation partitioning.

**Figure 3:**
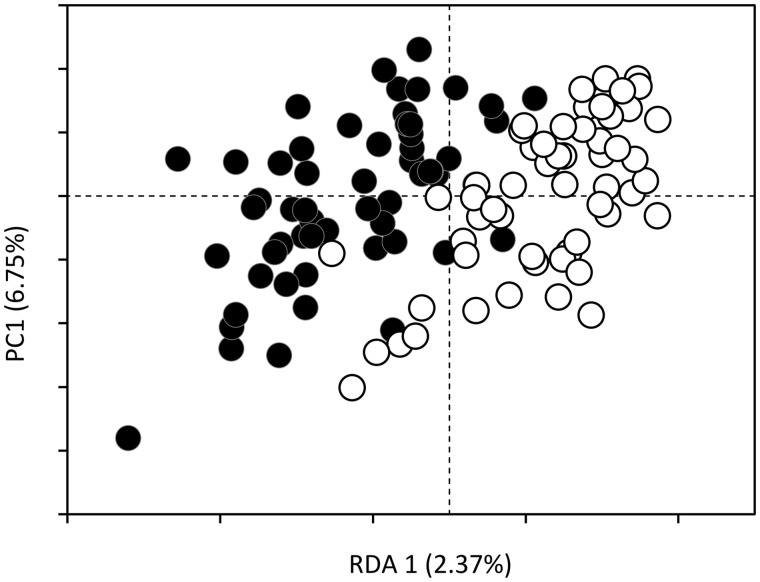
correlation of epigenetic variation with reproductive behavior. RDA of epigenetic variation for adult individuals sampled during reproductive (black circles) and non-reproductive (white circles) years

For juveniles, genetic variation among individuals and sampling sites explained 6.00% (*P *=* *0.002) and 6.77% (*P *=* *0.001), respectively of the epigenetic variation. However, we did not detect epigenetic differences between juveniles born following spring or summer reproduction (*R*^2^ = 2.50%; *P *=* *0.119). For adults, we also detected significant pure effect of genetic variation (*R*^2^ = 2.71%; *P *=* *0.001) and sampling site (*R*^2^ = 2.61%; *P *=* *0.001) on epigenetic variation. When taking into account these genetic and sites effects, we detected a significant correlation between epigenetic variation and reproductive periods categorized according the presence/absence of newly produced juveniles (*R*^2^ = 2.37%; *P *=* *0.001; Fig. 3). The same signals were also detected when we took into account the amount of beech seed produced the fall following individuals sampling (*R*^2^ = 2.00%; *P *=* *0.001).

Variation partitioning confirmed significant effect of reproductive periods on epigenetic variation for individuals sampled in site 1 (*R*^2^ = 4.02%; *P *=* *0.002) and site 2 (*R*^2^ = 3.42%; *P *=* *0.032). At the opposite, while variation of beech seed production was still observed in site 3, only 2017 displayed the highest beech seed production associated with chipmunk reproduction (Fig. 1). Interestingly, for site 3 epigenetic variation among individuals sampled in spring could not be explained by only the quantity of beech seed produced in the following fall (*R*^2^ = 5.60%; *P *=* *0.072). Rather variation in DNA methylation was correlated with the reproductive periods, i.e. the association of beech seed production and the presence of juveniles in the fall (*R*^2^ = 6.67%; *P *=* *0.006), resulting from summer reproduction specific to sampling site.

### Epigenetic Variation for Individuals Sampled Twice

**Figure 4:**
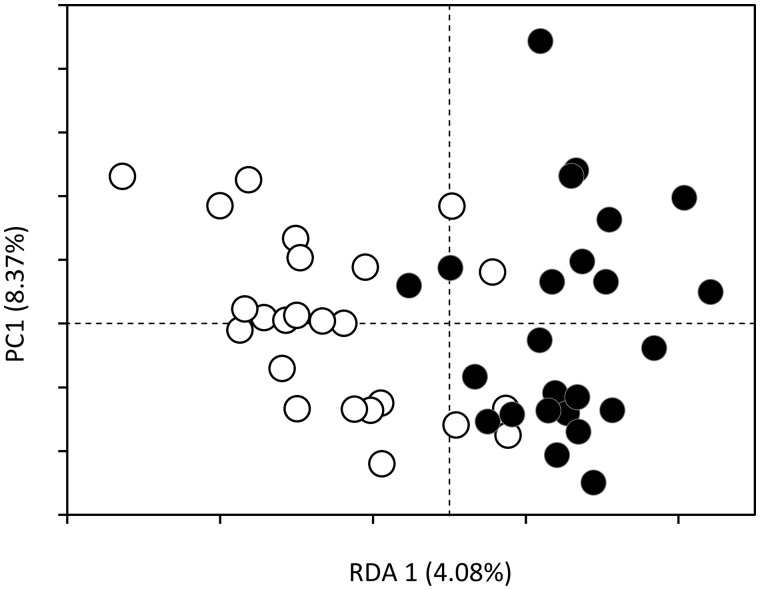
epigenetic variation of individuals sampled twice. RDA of epigenetic variation for adult individuals sampled in both reproductive (black circles) and non-reproductive (white circles) years

Partitioning of epigenetic variation among the 24 adults sampled during two distinct years revealed high individual effect (*R*^2^ = 51.97%; *P *=* *0.002). While 76.33% of the epigenetic loci remained identical between the two sampling years, individual displayed on average 29.58 (7.2 SD; 23.64%) different loci between reproductive and non-reproductive years. The number of different epigenetic loci between no-summer reproduction and summer reproduction years was not different whether individuals were first sampled in 2014 (28, 8.83 SD loci) or 2016 (30.71, 6.07 SD loci). When we took into account these individual effects as conditioning variable, we still detected DNA methylation differences between reproductive and non-reproductive years (Fig. 4). The constrained ordination analyses resulted in a correlation between epigenetic variation and reproductive years (*R*^2^ = 3.90%; *P *=* *0.007) as well as beech seed fall production (*R*^2^ = 3.50%; *P *=* *0.013), underlining our hypothesis that DNA methylation in spring predicts food availability in fall.

## Discussion

The objective of this study was to detect an epigenetic signal of anticipation for food availability in chipmunks. In pulsed-resource systems characterized by episodic resource availability, reproduction is typically favored when resources are abundant [12]. We therefore predicted a correlation between individuals’ DNA methylation profile during the spring season, prior to reproduction, and beech seed production it the following fall.

As expected, we detected a significant correlation between variation of DNA methylation among adult eastern chipmunks and beech seed production. This supports the hypothesis that at every spring, chipmunks can undergo (or not) an epigenetic reprograming triggered by an environmental signal announcing future beech seed abundance. This conclusion was underlined by a consistent epigenetic signal for three genetically distinct populations facing similar environmental variation (e.g. reproduction and high beech seed productivity), as well as by distinct epigenetic profiles of individuals sampled during both a reproductive and a non-reproductive year.

### Genetic and Spatial Variation

We detected a significant epigenetic differentiation among the three sampling sites. This is expected due to the cumulative effects of the genetic variation and differences in environmental conditions among sites, representing the major sources explaining consistent spatial epigenetic variation [21, 25, 26]. Interestingly, we can disentangle both genetic and sampling site effects on epigenetic variation by partialling out the genetic variation from MspI treatment. This portion of epigenetic variation that is significantly explained by sampling site could be associated to environmental influence on epigenetic variation, if considering sampling sites as a proxy for environmental conditions.

We also detected differences between the epigenetic profiles of adult and juvenile individuals. In addition to physiological differences specific to these developmental stages, different environmental conditions may be responsible of these epigenetic differences. For instance, juveniles remained in the maternal burrow and undergoing maternal care the majority of their life prior to DNA sampling at emergence while adults have experienced different environments out of the burrow.

Our results confirmed therefore the genetics and environmental sources of epigenetic variation among individuals and, foremost, underlined the appropriate resolution of our MSAP markers for detecting environmentally induced epigenetic variation. In addition to spatial variation, several individual components may influence epigenetic variation. This includes genetic differences, random epimutations, individual temperament/personality, social interactions, health, specific life history, etc. explaining the high proportion (>50%) of total epigenetic variation specific to each individual [20, 27–31].

### Temporal Epigenetic Variation: Anticipation for Food and Reproduction

As observed in [14], chipmunks summer reproduction was again correlated with beech seeds production in the fall, underlining chipmunks reproductive behavioral plasticity. Environmentally induced epigenetic changes could underline the capacity of a given genotype to adjust its phenotype to match the environment [20]. Chipmunks experienced various environmental conditions during their lifespan, explaining the high epigenetic variation that we observed among individuals (>50%). Despite the high individual effect on the total epigenetic variation, we still detected a consistent, but weak, epigenetic signal among adult individuals sampled in years of low vs high food abundance, and the associated reproductive condition. This observation support our hypothesis that epigenetic processes underlie chipmunks’ plasticity of reproductive behavior to cope with annual fluctuation of beech seed production, while the environmental cue triggering this change remains unknown [14].

We assessed epigenetic variation in a punch of ear tissue. The goal of this study was not to find the specific locus responsible for reproductive behavior, but rather to detect a global signal related to reproductive behavior. Reproduction is associated to specific individual state [32, 33]. Indeed, the current epigenetic signal was not expected to directly be associated to genes specific to reproduction, but to the indirect conditions and global changes associated with reproductive behavior. This may include the result of environmental conditions specific to reproductive condition, like food type and abundance, intra- and interspecific interactions and other behaviors [33–35]. For instance, during years of no or low beech seed production, adults skipped summer reproduction and could remain in their burrows from July until the next spring [36]. Alternatively, chipmunks anticipated beech masting by mating in June, synchronizing juvenile emergence with ripening of beech seeds in September [14].

Episodic resource availability did not coincide among sites (e.g. site 3 compared to sites 1 and 2). While the general tendency was similar among the three sites, with higher beech seeds production during 2013, 2015 and 2017 compared to 2014 and 2016, they did not display the same beech seed production among years. At the opposite of sites 1 and 2, site 3 did not reach the threshold of 50 seeds per m^2^ resulting in the absence of summer reproduction in 2013 and 2015. This also suggest a non-cyclic pattern of reproduction in chipmunks. Interestingly, we detected a correlation between epigenetic signals and local seed production in sites 1 and 2, but not in site 3, suggesting that each site displayed its own signals of future beech seed availability in spring and underlining chipmunks reproductive behavior plasticity. While such signals remain to be elucidated, local ecology is of importance for chipmunks survival since they display a small home range. Therefore, in a locality displaying a seeds production reduction, individuals should be able to adjust their reproductive behavior to cope with such environmental constrain.

Finally, part of the measured DNA methylation differences between individuals sampled both during years of summer reproduction and no-summer reproduction could be the result of an accumulation of stochastic epimutations or environmentally induced variation resulting from individual experiences during their lifespan [30, 31]. Nonetheless, when comparing DNA methylation pattern between two samples of the same individual (i.e. reproductive vs non-reproductive years), we did not detect differences in terms of epigenetic differences, when comparing one-year interval (to 2016 vs 2017) vs three-years interval (2014 vs 2017). This result indicates that the epigenetic variation measured in the current study could not be only attributed to accumulation of epigenetic modifications occurring during their lifespan, and supports the hypothesis of a consistent signal related to beech seed production and chipmunks’ reproductive behavior.

### Predictability of Future Environment

Phenotypic plasticity relies on the predictability of environmental changes to be advantageous [37–40]. At the same time, reproduction is often a relatively long and energetically costly process for many species [33]. Consequently, adaptive plasticity of reproductive behavior is expected to rely on environmental signals enabling the anticipation of food availability once juveniles will be born. In the context of this study, our results suggest that every spring, while chipmunks displayed in general similar DNA methylation profile from one year to another, they can undergo an epigenetic resetting and reprograming at some loci, triggered by an environmental signal announcing future beech seed abundance.

The cues leading to chipmunks’ reproduction remain to be determined, but several hypotheses may be advanced. For instance, the observed epigenetic differences between reproductive and non-reproductive years, could be triggered by abiotic factors, such as winter/spring average temperatures and precipitations, presence of predators or other biotic interactions [41], secondary plant components [42], or quantity of food-resources both during the winter or the spring including insects, berries, nuts or others plants [43]. For instance, chipmunks shift their diet to increase red maple consumption early in spring prior to a summer reproduction, which is also strongly correlated with beech seed production in the fall [44]. To further address these questions, studies could experimentally modify resources availability to better understand the proximate effects that correlated factors can have on epigenetic reprogramming.

One cost fatally associated with phenotypic plasticity is the consequences of ‘unreliable’ signals. When the predicted and actual environments differ, a mismatch between the developed phenotype and the actual environment could result in adverse consequences. For instance, some species like Snowshoe hares (*Lepus americanus*) can undergo seasonal molts to white or brown coat to match the presence or absence of snow, allowing a background matching and thus a camouflage strategy to reduce risks of predation [45]. Therefore, the environmental signal (photoperiod) triggering the changes of individuals’ seasonal color became a non-reliable environmental signal to future background coloration, because snow cover duration decreases due to climate change. As a result, coat color mismatched with the background, which resulted in an increased of predation and decreased survival among hares [46, 47]. Similarly, Bergeron *et al.* reported reproduction of chipmunks during the summer 2009 in anticipation of a mast [14]. However, beech seed production in the following autumn was actually very low, as most seed coat were empty, which resulted in a lower than average juvenile survival rate in the following winter.

In conclusion, the epigenetic variation represents a powerful tool to investigate anticipation of organisms. Epigenetics enable the detection of anticipatory response even when specific environmental signals are unknown for a given organism. Further epigenomic analyses may identify genes involved in the phenotypic change. These toolkits could provide relevant information to orient ecological experimentations.

## Methods

### Sampling

We sampled individuals from three different sites within a 10-km area, in the Mont Sutton region, southern Quebec (Canada), as part of a long-term study [24]. Because eastern chipmunks are solitary rodents with small home range [42], the different sampling locations represent distinct populations [24, 48]. We used Longworth traps checked at 2-h intervals during their active period, every week from the end of April to September (see [14, 24] for detailed protocol). Each individual was marked with unique alphanumerical ear tags, sexed and aged. We used the mass at emergence, with a threshold of 80 g, or the absence of darkened scrotum or developed mammae to distinguish juvenile from adult individuals. We assigned juveniles to spring or summer reproduction when the first capture occurred prior to or after August 1, respectively. We calculated chipmunk average reproductive success as the ratio of the number of juveniles to the number of adult females captured after August 1 for summer reproduction [14].

For each site and for each year, we quantified fall seed production using plastic buckets placed under the canopy of beech trees having a diameter at breast height of ≥10 cm as described in [49]. All seed coats were open, nuts counted and tree production calculated as the average number of seed per m^2^ per site.

We obtained DNA samples from ear-punch of each individual. Juveniles were sampled at maternal burrow emergence during spring 2014 (*n* = 27) and falls 2013 and 2015 (*n* = 3 and 18, respectively), while adult individuals (*n* = 114) were sampled in May, during 4 years (2013, 2014, 2016 and 2017) early in the spring, prior to summer reproduction period (Table 1, Supplementary Material S1). The sampling included 25 individuals sampled twice for biological replicates, first in 2014 or 2016 and the second time in 2017. Interestingly, this double sampling included 24 adults that allowed assessing epigenetic changes during their lifetime while experiencing distinct environmental conditions, separated by 1 or 3 years. The choice of tissue to assess the anticipatory response to reproduction was dictated by two factors: (i) a non-invasive tissue sampling to minimize the stress of manipulations; (ii) a tissue not directly involved in the reproduction process to avoid intrinsic differences between males and females for a common anticipatory response.

### Methyl-Sensitive Amplification Polymorphism

DNA extractions from ear-punch tissues were conducted using a salting out method [24, 50]. To detect methylation patterns in the different DNA samples, we carried out MSAP, a modified AFLP technique using methylation-sensitive restriction enzyme (MspI and HpaII) digestion [51, 52]. This method allowed the assessment of both genetic and DNA methylation patterns, on a genome-wide level by using a subsample of the whole variation, for a large number of individuals. Briefly, we used EcoRI as the rare cutter restriction enzyme and MspI and HpaII as the frequent cutter ones. For both MspI and HpaII digestion treatments, pre-selective amplification involved EcoRI_A and Msp*/*HpaII_C extremities. We thereafter performed five selective PCRs using the following combination of primers, for respectively Eco_ANN and MspI/Hpa_CNN: AAC/CTC, AAC/CAG, ACG/CTC, AAC/CCT and ACG/CCG. Loci were separated by electrophoresis on denaturing 6% polyacrylamide (19:1 acrylamide: bis-acrylamide) gels and visualized using silver nitrate staining [53].

We constructed a presence/absence matrix for both genetic and epigenetic variation from banding patterns resulting from MspI and HpaII treatments, respectively. However, we did not consider loci within the HpaII treatment associated with polymorphic loci within the MspI treatment to construct the epigenetic matrix in order to avoid loci likely associated with genetic variation.

In order to assess the consistency of PCR-amplified loci, we carried out biological and technical replicates. Biological replicates were performed by comparing the genetic profiles from tissues of 25 individuals sampled twice, each individuals sampled during two distinct years. Technical replicates consisted of the same DNA sample for which we performed the whole MSAP analyses (genetic and epigenetic banding patterns) independently twice. We used 16 individuals as technical replicates. We did not consider loci that inconsistently amplified or occurred at highly variable intensities between two replicates for subsequent statistical analyses.

### Statistical Analyses

To visualize both genetic and epigenetic variation among individuals, we computed a principal component analysis (PCA) with the presence/absence matrix resulting from MSAP analyses. We used a multi-variate statistical approach to assess how groups of markers covary in response to environmental predictors, as multi-variate analyses enable to consider many loci simultaneously and, thus, to detect multi-locus molecular signatures [54, 55]. To estimate the amount of genetic differentiation among sites, we calculated the Jaccard similarity coefficient among each individual from the MspI matrix to avoid taking into account mutual absence of loci. We thereafter constructed the genetic matrix by performing a principal coordinate analysis (PCoA). We tested whether eastern chipmunks displayed genetic differences among sites by using redundancy analyses (RDA) [56]. PCoA of genetic distance among individuals constituted the response variable with the different sites as explanatory variable. We assessed the significance of constraints by computing an ANOVA-like permutations test using 999 randomizations [57].

To determine the influence of different predictors on the total epigenetic variation, we first generated an automatic stepwise model building for constrained ordination [58]. The total epigenetic patterns obtained from the presence/absence HpaII treatment matrix was the response variable, and the model included individuals’ age and sex, the geographical site (site 1, 2 or 3), reproductive period (reproductive, non-reproductive), quantity of beech seed production during fall of sampling years and genetic distance among individuals as described above as the explanatory variables (Table 1).

Subsequently, we estimated the proportion of epigenetic variation explained by the different explanatory variables that remained significant from the previous stepwise model building analyses with partial redundancy analyses (pRDA) [56]. The marginal effect of the different factors was based on the *R*^2^ and significance of each fraction was tested by ANOVA-like permutation tests using 999 randomizations [57, 59]. We computed all statistical analyses using the statistical programming environment R version 3.5.3 [60] with the *vegan* package version 2.5-4 [61] for the multi-variate analyses.

## Supplementary Material

dvz026_Supplementary_Data
