# Supplementary material for: Epigenetic anticipation for food and reproduction
Source: Environ Epigenet. 2020 Jan 30;6(1):dvz026. doi: 10.1093/eep/dvz026 (PMC6991620; doi:10.1093/eep/dvz026)
Supplement: dvz026_Supplementary_Data [file dvz026_supplementary_data.pdf]

# 1) List of individuals.

| Ind | Sex | Age | Site |
|---|---|---|---|
| F023 | F | A | Site_1 |
| H002 | F | A | Site_1 |
| H004 | F | A | Site_1 |
| H007 | F | A | Site_1 |
| H024 | F | A | Site_1 |
| H042 | M | A | Site_1 |
| I011 | M | A | Site_1 |
| I017 | M | A | Site_1 |
| I018 | M | A | Site_1 |
| I019 | M | A | Site_1 |
| I020 | F | A | Site_1 |
| I021 | F | A | Site_1 |
| I026 | F | A | Site_1 |
| I029 | F | A | Site_1 |
| I030 | M | A | Site_1 |
| I031 | M | A | Site_1 |
| I036 | F | A | Site_1 |
| I037 | F | A | Site_1 |
| I038 | M | A | Site_1 |
| I039 | M | A | Site_1 |
| J002 | M | A | Site_1 |
| J004 | F | A | Site_1 |
| J007 | F | A | Site_1 |
| J009 | F | A | Site_1 |
| J010 | F | A | Site_1 |
| J011 | M | A | Site_1 |
| J017_2014 | F | J | Site_1 |
| J017_2017 | F | A | Site_1 |
| J019 | F | A | Site_1 |
| J020 | M | A | Site_1 |
| J021 | M | A | Site_1 |
| J022 | F | A | Site_1 |
| J026 | F | A | Site_1 |
| J028 | M | A | Site_1 |
| J030 | M | A | Site_1 |
| J043 | F | A | Site_1 |
| J047 | F | A | Site_1 |
| J048 | M | A | Site_1 |
| J051 | M | A | Site_1 |
| J052 | M | J | Site_1 |
| J054 | F | A | Site_1 |
| J061 | F | J | Site_1 |
| J070 | F | A | Site_1 |
| J078 | F | A | Site_1 |
| J082 | F | J | Site_1 |
| J084 | F | J | Site_1 |

| Ind | Sex | Age | Site |
|---|---|---|---|
| J087 | M | J | Site_1 |
| J092 | F | A | Site_1 |
| J122 | M | J | Site_1 |
| J147 | M | J | Site_1 |
| K025 | F | J | Site_1 |
| K027 | M | J | Site_1 |
| K029 | M | J | Site_1 |
| K034 | M | J | Site_1 |
| K035 | M | J | Site_1 |
| K037 | F | J | Site_1 |
| K040 | F | J | Site_1 |
| K053 | M | J | Site_1 |
| K062 | M | J | Site_1 |
| L009 | M | A | Site_1 |
| L010 | M | A | Site_1 |
| L011 | M | A | Site_1 |
| L013 | M | A | Site_1 |
| L014 | M | A | Site_1 |
| L015 | M | A | Site_1 |
| L016 | M | A | Site_1 |
| L027 | F | A | Site_1 |
| L056 | F | A | Site_1 |
| L060 | M | A | Site_1 |
| I001 | F | A | Site_2 |
| I002 | F | A | Site_2 |
| I003 | F | A | Site_2 |
| I004 | F | A | Site_2 |
| I006 | M | A | Site_2 |
| I013 | M | A | Site_2 |
| I014 | F | A | Site_2 |
| I015 | F | A | Site_2 |
| I025 | F | A | Site_2 |
| I033 | F | A | Site_2 |
| I034 | F | A | Site_2 |
| I050 | F | A | Site_2 |
| I052 | F | A | Site_2 |
| I075 | F | J | Site_2 |
| I078 | F | J | Site_2 |
| I082 | F | J | Site_2 |
| J006 | F | A | Site_2 |
| J008 | F | A | Site_2 |
| J014 | M | A | Site_2 |
| J015 | M | A | Site_2 |
| J023 | M | A | Site_2 |
| J032 | M | A | Site_2 |
| J035 | F | A | Site_2 |

| Ind | Sex | Age | Site |
|---|---|---|---|
| J036 | M | A | Site_2 |
| J037 | M | A | Site_2 |
| J041 | M | A | Site_2 |
| J049 | F | J | Site_2 |
| J058 | M | J | Site_2 |
| J064 | M | A | Site_2 |
| J065 | F | J | Site_2 |
| J066 | M | A | Site_2 |
| J068 | M | A | Site_2 |
| J069 | F | J | Site_2 |
| J088 | M | A | Site_2 |
| J089 | M | J | Site_2 |
| J090 | M | J | Site_2 |
| J091 | F | J | Site_2 |
| J104 | F | J | Site_2 |
| J106 | F | A | Site_2 |
| J108 | M | A | Site_2 |
| J144 | M | J | Site_2 |
| K046 | F | J | Site_2 |
| K050 | M | J | Site_2 |
| K051 | M | J | Site_2 |
| K058 | F | J | Site_2 |
| K059 | F | J | Site_2 |
| K060 | F | J | Site_2 |
| K061 | F | J | Site_2 |
| K063 | F | J | Site_2 |
| K064 | M | J | Site_2 |
| L017 | M | A | Site_2 |
| L037 | M | A | Site_2 |
| L039 | M | A | Site_2 |
| L043 | F | A | Site_2 |
| L048 | F | A | Site_2 |
| L092 | F | A | Site_2 |
| L097 | F | A | Site_2 |
| L099 | M | A | Site_2 |
| L102 | F | A | Site_2 |
| L103 | M | A | Site_2 |
| H056 | F | A | Site_3 |
| H058 | F | A | Site_3 |
| H062 | F | A | Site_3 |
| H063 | F | A | Site_3 |
| H065 | F | A | Site_3 |
| H068 | F | A | Site_3 |
| H073 | M | A | Site_3 |
| J101 | M | J | Site_3 |
| J102 | F | A | Site_3 |

| Ind | Sex | Age | Site |
|---|---|---|---|
| J103 | M | J | Site_3 |
| J110 | M | J | Site_3 |
| J111 | M | A | Site_3 |
| J113 | F | J | Site_3 |
| J116 | F | J | Site_3 |
| J117 | M | J | Site_3 |
| J118 | F | J | Site_3 |
| J120 | M | J | Site_3 |
| J121 | M | J | Site_3 |
| J124 | M | J | Site_3 |
| J129 | M | A | Site_3 |
| J132 | F | A | Site_3 |
| J133 | F | A | Site_3 |
| J134 | M | A | Site_3 |
| J135 | M | A | Site_3 |
| L002 | M | A | Site_3 |
| L003 | F | A | Site_3 |
| L004 | M | A | Site_3 |
| L005 | F | A | Site_3 |
| L006 | F | A | Site_3 |
| L020 | F | A | Site_3 |
| L035 | M | A | Site_3 |
| L087 | F | A | Site_3 |
| L090 | M | A | Site_3 |

F: Female
M: Male
J: Juvenile
A: Adult

**# 2) Genetic profiles from MSAP analysis:** loci from *MspI* treatment. For each individual, ID and sampling year are provide.

Ind_year,Sel1-330,Sel1-297,Sel1-239,Sel1-235,Sel1-230,Sel1-227,Sel1-220,Sel1-214,Sel1-211,Sel1-193,Sel1-187,Sel1-185,Sel1-174,Sel1-161,Sel1-155,Sel1-151,Sel1-150,Sel1-143,Sel1-141,Sel1-121,Sel2-271,Sel2-258,Sel2-250,Sel2-233,Sel2-167,Sel2-155,Sel2-154,Sel2-148,Sel3-381,Sel3-370,Sel3-350,Sel3-341,Sel3-332,Sel3-292,Sel3-290,Sel3-288,Sel3-275,Sel3-267,Sel3-265,Sel3-256,Sel3-242,Sel3-236,Sel3-189,Sel3-155,Sel3-133,Sel3-130,Sel3-108,Sel3-103,Sel4-325,Sel4-307,Sel4-306,Sel4-305,Sel4-294,Sel4-255,Sel4-244,Sel4-239,Sel4-225,Sel4-222,Sel4-210,Sel4-200,Sel4-190,Sel4-169,Sel4-162,Sel4-160,Sel4-143,Sel4-138,Sel4-131,Sel5-340,Sel5-258,Sel5-225,Sel5-194,Sel5-164,Sel5-159,Sel5-158
F023_2013,1,0,1,1,0,0,0,1,1,0,0,1,1,1,0,1,1,0,0,0,1,1,0,1,0,1,0,1,1,1,1,1,1,1,0,1,1,0,1,0,1,1,0,0,0,1,1,1,1,0,1,1,0,1,1,0,1,1,1,0,1,1,1,1,0,1,1,0,0,0,0,1,1,0
H002_2013,0,0,1,0,0,0,1,1,0,0,0,1,1,1,1,1,1,0,0,1,1,0,1,1,1,1,0,1,1,1,1,1,1,1,1,1,0,0,0,1,0,0,0,0,1,0,1,1,1,1,0,1,1,1,1,1,1,1,1,1,0,1,1,1,1,0,1,1,0,0,0,0,0,1,1
H004_2013,1,1,1,1,0,0,1,1,1,0,0,1,1,1,0,1,1,0,1,1,1,1,0,1,0,1,1,1,1,1,1,1,1,1,1,0,1,0,1,1,0,1,0,1,0,0,1,1,1,1,0,1,1,1,1,1,0,1,1,1,0,1,0,0,0,0,1,1,1,0,0,1,0,1,1
H007_2013,0,0,1,1,0,0,0,1,0,0,1,1,1,1,0,1,1,0,0,0,1,1,1,1,0,0,0,1,1,1,1,1,1,1,1,0,0,1,0,1,1,1,0,0,0,0,0,1,0,1,1,1,1,1,0,1,1,1,1,1,0,1,1,1,1,0,1,1,1,0,0,0,1,1,1
H024_2013,0,0,1,0,0,0,1,1,1,0,0,1,1,1,1,0,1,0,1,1,1,1,0,0,0,0,0,1,1,1,1,1,1,1,1,0,1,0,0,1,0,1,0,1,1,0,1,1,1,1,1,1,1,1,1,1,1,1,1,1,1,0,0,1,1,1,0,1,1,0,0,1,0,0,1,1
H042_2013,1,0,1,1,0,0,1,1,0,1,1,1,1,1,0,0,1,0,1,0,1,1,1,0,0,0,1,1,1,1,1,1,1,1,1,0,1,0,1,1,0,1,1,0,0,0,1,1,0,1,1,1,1,1,0,1,1,1,1,1,0,1,0,1,1,0,1,1,0,0,1,0,0,0,1
H056_2013,1,0,1,1,0,0,0,1,1,1,0,1,1,1,0,1,0,0,0,1,1,1,1,0,0,0,1,1,1,1,1,1,0,1,1,1,1,0,0,1,1,1,1,0,0,0,1,1,1,1,1,1,1,1,1,1,1,0,1,1,1,0,0,1,1,1,1,1,0,1,0,0,0,1,1,1
H058_2013,0,0,1,1,0,0,1,1,1,0,0,1,1,1,0,1,1,0,1,0,1,0,0,0,0,0,1,1,1,1,1,1,1,1,1,0,0,1,0,1,0,1,0,1,1,0,1,1,1,1,1,1,1,1,0,0,1,1,1,1,1,0,0,0,1,0,0,1,1,0,0,1,0,1,1,1
H062_2013,1,1,1,1,0,0,0,1,1,1,0,1,1,1,1,1,1,1,0,1,1,1,1,0,1,1,0,1,1,1,1,1,1,1,1,1,0,1,0,0,0,1,1,0,0,1,1,0,1,1,0,1,1,1,1,0,1,1,1,1,1,1,1,0,1,1,1,0,0,1,1,1,1,1,1,1,0,1,0
H063_2013,1,0,1,1,0,0,0,1,1,0,0,1,1,1,1,1,1,1,0,1,1,1,1,0,0,1,1,1,0,0,1,1,1,1,1,1,1,1,0,0,1,1,1,1,1,1,1,0,0,1,1,1,1,1,1,1,0,1,1,1,1,1,1,1,0,0,0,1,0,0,1,1,1,0,1,0,1,1,1
H065_2013,1,0,1,0,0,0,1,0,0,0,0,1,1,1,0,1,0,0,0,0,1,0,1,0,0,1,0,1,1,1,0,1,1,1,0,1,0,1,0,0,1,1,0,1,0,0,1,1,1,1,1,1,0,0,1,1,1,1,1,0,1,1,0,0,0,1,0,1,0,1,0,1,1,0
H068_2013,1,0,1,0,0,0,0,1,1,0,0,1,1,1,0,1,0,0,1,0,0,1,0,1,0,0,0,1,1,1,1,1,1,1,1,1,1,0,1,1,0,1,0,1,1,0,1,1,1,1,1,1,1,1,1,1,1,1,1,1,1,0,0,1,1,0,0,1,1,1,1,0,0,0,1,1
H073_2013,1,0,1,1,0,0,0,0,0,0,0,1,1,1,1,1,1,0,1,0,1,1,1,1,1,0,0,1,1,1,1,1,1,1,1,0,1,1,0,1,0,1,0,1,0,0,1,1,0,1,1,1,1,1,1,1,1,1,1,1,0,0,1,0,0,0,1,1,0,0,0,0,0,1,1
I001_2013,1,1,1,1,0,0,0,1,0,0,1,0,0,1,0,1,0,0,0,1,1,1,1,0,0,1,1,1,1,1,1,1,1,1,1,0,0,0,0,1,1,1,0,1,1,0,1,1,1,1,1,1,1,1,1,1,0,1,1,1,0,1,0,1,0,0,1,1,1,1,1,1,1,1,1
I002_2013,0,0,1,1,0,0,0,1,1,1,1,0,1,1,0,1,0,0,1,1,1,0,1,0,1,1,1,1,1,1,1,0,1,0,1,0,0,0,0,1,1,1,0,0,1,1,1,1,1,1,1,1,1,1,1,1,1,1,0,1,1,0,1,1,0,1,0,0,0,1,0,1,0,1,0,1
I003_2013,0,0,1,1,0,0,1,1,1,0,1,1,1,1,1,1,1,0,1,1,0,1,0,1,1,0,1,1,0,1,1,1,1,1,1,1,1,1,0,1,0,1,1,0,0,0,1,1,1,0,0,1,1,0,1,1,1,1,1,1,1,0,1,1,1,0,1,1,1,0,0,0,0,0,1,1
I004_2013,1,1,1,1,0,0,1,1,1,0,0,1,1,1,0,1,1,0,1,1,1,1,0,1,0,1,0,1,1,1,1,1,1,1,1,0,1,1,1,1,0,1,0,1,1,0,1,1,1,1,1,1,1,1,1,1,1,1,1,1,1,0,1,1,0,0,0,1,1,1,1,1,1,1,1,1
I006_2013,1,1,1,1,0,0,1,1,1,0,1,1,1,1,0,1,1,1,1,1,1,1,0,0,0,1,1,1,1,1,1,1,1,1,1,1,1,0,1,1,1,1,1,1,1,1,0,1,1,1,1,1,1,1,1,0,1,1,1,1,1,1,0,0,1,0,1,0,1,1,1,0,0,0,1,1,0
I011_2013,1,1,1,1,0,0,1,1,1,0,0,1,1,1,1,1,0,0,1,0,0,0,0,0,0,0,1,1,0,1,1,0,1,1,1,1,0,0,1,0,0,1,0,0,0,1,1,0,1,1,1,1,0,1,1,1,1,1,1,0,0,1,1,1,1,1,1,0,0,0,0,0,0,1
I013_2013,0,0,1,1,0,0,1,1,1,0,0,1,1,1,0,1,1,0,1,1,1,1,1,1,1,1,1,0,1,0,1,1,1,1,1,1,1,1,0,0,1,0,1,1,0,0,0,1,1,1,1,1,1,1,1,0,1,1,1,1,1,1,0,1,1,1,1,0,1,1,1,0,1,0,1,1,0
I014_2013,0,0,1,0,0,0,0,1,1,0,0,1,1,1,0,1,1,0,1,1,0,1,0,1,1,1,1,1,1,1,1,1,1,1,1,1,0,0,0,1,0,1,1,0,0,0,1,1,1,1,0,1,1,0,1,1,1,1,1,1,0,0,1,1,1,0,1,1,0,1,0,0,1,1,1
I015_2013,1,0,1,1,0,0,1,1,1,0,1,1,1,1,0,1,0,0,1,1,0,1,0,1,1,1,0,1,1,1,1,1,1,1,1,0,0,0,0,1,0,1,1,1,1,0,1,1,1,1,0,1,1,0,1,1,1,1,1,1,0,0,0,0,1,0,1,1,0,0,0,0,1,0,1
I017_2013,0,0,1,1,0,0,1,1,0,0,0,0,1,1,0,1,0,0,1,0,1,0,0,0,0,1,0,1,1,1,0,1,1,0,0,1,0,0,1,0,1,0,1,1,0,1,1,1,1,0,1,1,0,1,1,1,1,1,1,0,1,1,0,0,0,1,1,0,0,1,0,0,0,1
I018_2013,1,1,1,1,0,0,1,1,1,0,1,1,1,1,1,1,1,0,1,1,1,1,1,1,1,1,1,1,1,1,1,1,1,1,1,1,0,1,0,1,0,1,0,1,1,0,1,1,1,1,0,1,1,1,1,1,1,1,1,1,1,0,1,1,1,1,0,1,1,0,1,1,0,1,1,1
I019_2013,0,0,1,1,0,0,0,1,1,0,0,1,1,1,1,1,1,0,1,1,1,0,0,0,0,0,1,1,0,1,1,1,1,1,1,1,0,1,0,1,0,1,0,1,1,0,0,1,1,1,0,1,1,1,1,1,1,1,1,1,0,1,1,1,1,0,1,1,1,0,1,1,1,1,1
I020_2013,1,1,1,1,0,0,0,1,1,0,0,1,1,1,0,1,1,0,1,1,1,1,0,1,1,0,0,1,1,1,1,1,1,1,1,1,1,0,0,1,0,1,0,0,0,0,1,1,1,1,0,1,1,1,1,1,1,1,1,1,1,0,1,1,0,1,0,1,1,0,0,1,1,0,0,1
I021_2013,0,1,1,1,0,0,1,1,1,0,0,1,1,1,0,1,1,0,1,0,1,1,1,0,1,1,0,1,1,1,1,1,1,1,1,0,1,0,0,1,1,0,0,1,1,0,1,1,1,1,0,1,1,1,1,1,1,1,1,1,0,1,1,0,1,0,1,1,0,0,1,0,0,1,1
I025_2013,1,0,1,1,0,0,1,1,1,0,0,1,1,1,0,1,1,0,1,1,0,1,0,1,1,1,0,1,0,1,1,1,0,1,0,1,1,1,1,0,1,1,0,0,0,1,1,0,1,0,1,1,1,0,1,1,1,1,1,0,1,0,1,1,0,1,1,0,1,0,1,0,1,0
I026_2013,0,1,1,1,0,0,1,1,1,0,0,0,1,1,0,1,1,0,0,1,1,1,1,1,0,1,0,1,1,1,0,1,1,1,0,0,1,0,1,0,1,1,1,0,0,1,1,1,1,0,1,1,1,1,1,1,1,1,0,0,1,1,0,1,0,1,0,0,0,1,0,0,1,1
I029_2013,0,0,1,1,0,0,1,1,1,1,0,1,1,1,0,1,1,0,0,1,1,1,0,1,0,1,0,1,1,1,1,1,1,1,1,0,1,0,0,1,0,1,0,1,1,0,1,1,1,1,1,1,1,1,1,1,1,1,1,1,1,0,1,1,1,0,1,1,1,0,0,0,0,1,1
I030_2013,1,0,1,1,0,0,1,1,1,0,0,1,1,1,1,1,0,0,1,0,0,1,0,1,1,1,1,1,1,1,1,1,1,1,1,0,1,1,0,1,0,1,1,1,1,0,1,0,0,1,1,1,1,1,1,1,1,1,1,1,0,1,1,1,1,0,1,1,0,0,0,1,1,1,0
I031_2013,1,1,1,1,0,0,1,1,1,0,0,1,1,1,1,1,1,0,1,1,1,1,1,1,1,1,1,1,1,1,1,1,1,1,1,0,0,0,0,1,1,1,0,0,1,0,1,1,1,1,1,1,1,1,1,1,1,1,1,1,1,0,1,0,1,0,1,1,0,0,1,1,0,0,1
I033_2013,1,0,1,1,0,0,0,1,1,0,1,1,1,1,1,1,1,0,1,1,0,1,0,0,1,1,0,1,1,1,1,1,1,1,1,0,0,0,0,1,0,1,1,1,0,0,1,1,1,1,0,1,1,1,1,0,1,1,1,1,0,0,1,1,0,0,1,1,0,0,0,0,1,1,1
I034_2013,1,1,1,1,0,0,1,1,1,0,0,1,1,1,0,1,0,0,1,1,1,1,0,1,0,0,1,1,1,1,1,1,1,1,1,1,1,1,0,1,0,1,0,0,1,0,1,1,1,1,0,1,1,1,1,1,1,1,1,1,1,1,1,1,1,1,0,1,1,1,1,1,0,0,1,1
I036_2013,1,1,1,1,0,0,1,1,1,1,1,1,1,1,1,1,1,0,1,1,1,1,1,0,0,1,0,1,1,1,1,1,1,1,1,1,0,0,1,1,0,1,1,1,1,0,1,1,1,1,0,1,1,1,1,1,1,1,1,1,0,1,1,1,0,1,1,1,1,1,1,0,1,1,1
I037_2013,1,0,1,1,0,0,0,1,1,1,0,1,1,1,0,1,1,0,0,0,1,0,0,1,1,0,1,1,1,1,1,1,1,1,0,1,1,1,0,1,0,1,0,0,1,0,1,1,1,1,0,1,1,1,1,1,1,1,1,1,0,0,1,0,0,0,1,1,0,0,1,0,1,1,1
I038_2013,1,1,0,1,0,0,1,1,1,0,0,1,1,1,0,1,1,0,1,1,1,0,0,1,0,1,1,1,1,1,1,1,1,1,1,0,1,0,0,1,0,1,0,0,0,0,1,1,1,1,1,1,1,1,1,1,1,1,1,1,0,0,0,0,1,0,1,1,1,0,0,1,1,1,1
I039_2013,1,1,1,1,0,0,1,1,1,0,0,1,1,1,0,1,1,0,1,0,1,1,1,1,1,1,1,1,1,1,1,1,1,1,1,1,0,1,0,1,0,1,0,1,0,0,1,1,1,1,1,1,1,1,1,1,1,1,1,1,1,0,1,1,1,0,1,1,1,1,1,0,1,1,0,1
I050_2013,0,0,1,1,0,0,1,1,1,0,0,1,0,1,0,1,0,0,1,0,0,1,1,1,1,1,1,1,1,0,1,1,0,1,1,1,0,1,0,1,1,1,1,1,0,0,1,1,1,1,0,1,1,0,0,1,1,1,1,1,1,0,1,1,1,0,1,1,0,0,0,0,1,1,1
I052_2013,1,1,1,1,0,0,1,1,1,1,0,1,1,1,0,1,0,1,1,1,1,1,1,1,1,0,0,1,0,1,1,1,1,1,1,0,1,0,1,1,1,0,0,1,0,1,1,1,1,1,1,1,1,0,1,1,1,1,1,0,0,0,1,1,1,1,1,0,1,0,0,0,1,1
I075_2013,1,1,1,1,0,0,1,1,1,1,0,1,1,1,0,1,1,0,1,1,1,1,0,1,1,1,0,1,1,1,1,0,1,1,0,1,1,1,1,0,1,1,1,1,1,0,1,1,1,1,1,1,0,1,1,1,1,1,1,0,1,0,1,1,0,1,1,0,0,1,1,1,1,1
I078_2013,0,1,1,1,0,0,1,1,1,0,0,1,1,1,0,1,1,0,1,1,1,1,0,1,0,1,0,1,0,1,1,1,1,1,0,1,1,0,1,0,1,0,0,0,0,1,1,1,1,0,1,1,1,1,1,1,1,1,1,0,1,1,1,1,1,1,1,0,0,1,0,1,1,1
I082_2013,1,1,1,1,0,0,1,1,1,0,1,1,1,1,0,1,1,0,1,0,0,0,0,0,0,1,0,1,1,1,1,1,1,1,1,0,1,0,0,1,0,1,0,0,0,0,1,1,1,1,0,1,1,0,1,1,1,1,1,1,0,1,1,0,1,0,1,1,1,0,1,0,0,1,1
J002_2014,1,1,1,1,0,0,1,1,1,0,0,1,1,1,1,1,0,0,1,1,0,0,0,0,1,0,1,1,1,1,1,0,1,1,1,1,0,0,1,0,0,0,0,1,0,1,1,0,1,1,1,1,1,1,1,1,1,1,1,1,0,1,1,1,0,1,1,1,0,0,0,0,0,0,1
J004_2014,1,1,1,1,0,0,1,1,1,0,0,1,1,1,0,1,1,0,1,1,1,1,1,1,1,1,0,1,1,1,1,1,1,1,1,1,1,1,0,0,1,1,0,1,0,1,0,1,1,1,1,0,1,1,0,1,1,1,1,1,1,0,0,1,1,1,0,1,1,0,0,1,1,0,1,1
J004_2017,1,1,1,1,0,0,1,1,1,0,0,1,1,1,0,1,1,0,1,1,1,1,1,1,1,1,0,1,1,1,1,1,1,1,1,1,1,1,0,0,1,1,0,1,0,1,0,1,1,1,1,0,1,1,0,1,1,1,1,1,1,0,0,1,1,1,0,1,1,0,0,1,1,0,1,1
J006_2014,1,1,1,1,0,0,1,1,1,0,1,1,1,1,0,1,1,0,1,1,1,1,0,1,1,0,1,1,1,1,1,1,1,1,1,0,1,1,0,1,0,1,1,1,1,0,0,1,1,1,1,1,1,1,1,1,1,1,1,0,1,0,0,1,0,1,0,1,1,0,0,1,1,0,1,1
J007_2014,1,1,1,1,0,0,1,1,1,0,0,1,1,1,0,1,1,0,0,1,1,1,0,1,0,1,0,1,1,1,1,1,1,1,1,0,1,1,0,1,1,1,0,1,1,0,1,1,1,1,0,1,1,1,1,1,1,1,1,1,0,0,0,1,0,0,1,1,0,0,1,1,0,1,1

J007_2017,1,1,1,1,0,0,1,1,1,0,0,1,1,1,0,1,1,0,0,1,1,1,0,1,0,1,0,1,1,1,1,1,1,1,0,1,1,0,1,1,1,0,1,1,0,1,1,1,1,0,1,1,1,1,1,1,1,1,1,0,0,0,1,0,0,1,1,0,0,1,1,0,1,1
J008_2014,1,1,1,1,0,0,1,1,1,0,0,1,1,1,1,1,1,0,0,0,1,1,1,1,1,0,1,1,1,1,1,1,1,1,0,1,0,1,1,0,1,0,1,0,0,1,1,1,1,0,1,1,0,0,1,1,1,1,1,0,1,1,0,1,0,1,1,0,0,0,0,0,0,0
J009_2014,1,0,1,1,0,0,0,1,1,0,0,1,1,1,1,1,1,1,1,0,1,1,0,1,0,0,1,1,1,1,1,1,1,1,1,1,0,0,0,1,1,1,0,0,0,0,0,1,1,1,1,1,1,1,1,1,1,1,1,1,1,0,1,0,1,1,0,1,1,0,0,0,1,0,1,1
J010_2014,1,0,1,1,0,0,0,1,1,0,0,1,1,1,0,1,1,0,1,1,1,1,1,0,0,0,1,1,1,1,1,1,1,1,1,0,1,0,1,0,1,0,0,0,0,0,1,0,1,1,1,1,1,1,1,1,1,1,1,0,1,0,0,1,0,1,1,1,0,0,0,0,1,1
J010_2017,1,0,1,1,0,0,0,1,1,0,0,1,1,1,0,1,1,0,1,1,1,1,1,0,0,0,1,1,1,1,1,1,1,1,1,0,1,0,1,0,1,0,0,0,0,0,1,0,1,1,1,1,1,1,1,1,1,1,1,0,1,0,0,1,0,1,1,1,0,0,0,0,1,1
J011_2014,0,1,0,1,0,0,1,1,1,1,0,1,1,1,0,1,1,0,1,1,1,1,1,0,1,0,0,1,0,1,1,1,1,1,1,1,1,0,0,1,1,1,1,0,1,0,0,1,1,1,0,1,1,0,1,1,1,1,1,1,1,0,1,1,0,0,1,1,1,0,0,1,1,0,0,1
J014_2014,1,1,1,1,0,0,1,1,1,1,0,1,1,1,0,0,1,0,1,1,0,1,1,1,1,0,1,1,0,1,1,1,1,1,0,1,1,0,1,0,1,0,1,0,0,1,1,1,1,1,1,1,1,1,0,1,1,1,1,1,0,1,1,1,0,0,1,1,1,0,1,0,1,1,1
J015_2014,1,0,1,1,0,0,0,0,0,0,0,1,1,1,0,1,0,0,1,0,0,1,1,1,1,0,1,1,1,1,1,1,1,1,1,1,1,0,0,1,0,1,0,1,1,0,0,1,1,1,1,1,1,1,1,1,1,1,1,1,1,0,0,1,0,0,0,1,1,0,1,1,1,0,1,1
J017_2014,1,0,1,1,0,0,0,1,1,0,0,1,1,1,0,1,1,1,1,0,1,1,0,1,0,0,1,1,1,1,1,1,1,1,1,1,0,0,1,1,1,0,0,0,0,0,1,1,1,1,0,1,1,1,1,1,1,1,1,1,1,0,1,1,1,0,0,1,1,1,0,0,1,0,1,1
J017_2017,1,0,1,1,0,0,0,1,1,0,0,1,1,1,0,1,1,1,1,0,1,1,0,1,0,0,1,1,1,1,1,1,1,1,1,1,0,0,1,1,1,0,0,0,0,0,1,1,1,1,0,1,1,1,1,1,1,1,1,1,1,0,1,1,1,0,0,1,1,1,0,0,1,0,1,1
J019_2014,0,0,1,0,0,0,0,0,0,0,1,0,0,1,1,1,0,0,1,1,1,1,1,0,1,1,1,1,1,1,0,0,1,0,0,0,0,1,1,0,1,0,1,0,0,1,1,1,1,0,1,1,1,1,1,1,1,1,1,0,1,0,1,1,0,0,1,0,1,0,0,1,0,0
J020_2014,0,0,1,1,0,0,1,1,1,1,0,1,1,1,0,1,1,0,1,0,1,1,0,1,1,1,0,1,1,0,1,1,1,1,0,1,0,0,1,0,1,0,1,1,0,0,0,1,1,1,1,1,0,1,1,1,1,1,1,0,1,1,0,0,1,1,1,0,1,1,1,0,1,1
J020_2017,0,0,1,1,0,0,1,1,1,1,0,1,1,1,0,1,1,0,1,0,1,1,0,1,1,1,0,1,1,0,1,1,1,1,0,1,0,0,1,0,1,0,1,1,0,0,0,1,1,1,1,1,0,1,1,1,1,1,1,0,1,1,0,0,1,1,1,0,1,1,1,0,1,1
J021_2014,0,0,1,1,0,0,1,1,0,1,0,1,1,1,0,0,0,0,1,0,0,0,0,1,0,0,1,1,1,1,1,1,1,1,0,1,0,0,1,0,1,1,0,0,0,0,1,1,1,1,1,1,0,1,1,1,1,1,1,1,0,0,1,1,0,0,1,0,0,1,0,0,1,1
J022_2014,1,1,1,1,0,0,1,1,0,1,0,1,1,1,0,1,1,0,1,0,1,1,1,0,1,1,0,1,1,1,1,1,1,1,1,0,1,1,1,1,0,1,0,0,1,0,0,1,1,1,1,1,1,0,0,1,1,1,1,1,0,1,1,1,1,0,1,1,0,0,0,0,1,1,1
J023_2014,1,1,1,1,0,0,1,0,0,0,0,1,1,1,0,1,1,0,1,1,0,0,1,1,1,0,1,1,1,1,1,1,1,1,1,1,1,1,0,1,1,1,0,1,1,0,1,1,1,1,0,1,1,1,0,1,1,1,1,1,0,0,0,1,1,0,1,1,0,0,1,0,1,0,1
J026_2014,1,1,1,1,0,0,1,1,1,1,0,1,1,1,1,1,1,0,1,1,1,1,1,0,1,0,0,1,1,1,1,1,1,1,0,1,0,0,1,0,1,1,1,0,0,0,1,0,1,1,1,1,1,1,1,1,1,1,1,0,1,1,1,1,0,1,1,1,1,1,0,1,1,1
J026_2017,1,1,1,1,0,0,1,1,1,1,0,1,1,1,1,1,1,0,1,1,1,1,1,0,1,0,0,1,1,1,1,1,1,1,0,1,0,0,1,0,1,1,1,0,0,0,1,0,1,1,1,1,1,1,1,1,1,1,1,0,1,1,1,1,0,1,1,1,1,1,0,1,1,1
J028_2014,0,0,1,1,0,0,1,1,0,0,0,1,1,1,0,1,0,0,1,0,1,1,0,0,1,1,1,1,0,1,1,1,1,1,1,1,0,0,1,0,1,0,0,1,0,1,1,1,1,1,1,1,1,0,1,1,1,1,1,0,1,1,0,1,0,1,1,1,0,0,0,1,1,1
J030_2014,1,0,1,0,0,0,1,1,0,0,0,1,1,1,0,1,1,0,1,0,1,1,1,1,0,1,1,1,1,1,1,1,1,1,1,0,1,0,1,0,1,1,0,1,0,0,1,1,1,1,1,1,0,0,1,1,1,1,1,0,1,1,1,1,0,1,1,1,1,0,1,0,1,1,1
J032_2014,1,1,1,1,0,0,1,1,1,0,0,1,1,1,0,1,0,0,1,1,1,1,0,0,1,0,1,1,0,1,1,1,1,1,0,1,0,0,1,0,1,1,1,1,0,1,1,1,1,1,1,1,1,1,1,1,1,1,1,0,1,1,1,1,0,1,1,0,0,1,0,0,1,1
J032_2017,1,1,1,1,0,0,1,1,1,0,0,1,1,1,0,1,0,0,1,1,1,1,0,0,1,0,1,1,0,1,1,1,1,1,0,1,0,0,1,0,1,1,1,1,0,1,1,1,1,1,1,1,1,1,1,1,1,1,1,0,1,1,1,1,0,1,1,0,0,1,0,0,1,1
J035_2014,0,0,1,1,0,0,0,1,1,0,0,1,1,1,1,1,1,0,1,1,1,1,0,1,1,1,0,1,1,1,1,1,1,1,0,0,0,1,1,0,1,0,1,1,0,1,1,1,1,1,0,1,0,0,1,1,0,0,1,0,0,0,1,1,0,0,0,1,0,1,1,1,1,1
J036_2014,1,1,1,0,0,0,1,1,1,0,0,1,1,1,0,1,0,0,1,1,1,1,1,0,1,1,0,1,1,1,1,1,1,1,1,1,0,0,1,0,1,0,0,1,0,1,1,1,1,1,1,1,0,1,1,1,1,1,1,0,1,1,1,1,0,1,1,0,0,0,0,1,1,0
J037_2014,1,1,1,1,0,0,1,1,0,0,1,1,1,1,0,1,1,0,1,1,1,1,0,0,1,0,1,1,1,1,1,1,1,1,1,0,1,1,1,1,1,1,0,1,1,0,1,1,1,1,0,1,0,0,1,1,0,1,1,1,0,1,1,1,1,0,1,1,1,1,1,0,1,0,0
J041_2014,1,0,1,1,0,0,0,0,0,0,0,1,1,1,0,1,1,0,1,1,1,1,1,0,1,0,1,1,1,1,1,1,0,1,0,0,0,0,1,0,1,0,0,0,0,1,1,0,1,1,1,1,1,0,1,1,1,1,1,0,1,1,1,1,0,1,1,0,1,0,0,1,0,1
J043_2014,0,1,1,1,0,0,0,1,1,0,0,1,1,1,0,1,1,0,1,0,1,1,1,1,0,1,0,1,1,1,1,1,1,1,0,1,1,0,1,1,1,1,1,1,0,0,1,1,1,1,1,1,0,1,1,1,1,1,1,0,1,1,1,1,0,1,1,1,0,0,0,1,1,1
J043_2017,0,1,1,1,0,0,0,1,1,0,0,1,1,1,0,1,1,0,1,0,1,1,1,1,0,1,0,1,1,1,1,1,1,1,0,1,1,0,1,1,1,1,1,1,0,0,1,1,1,1,1,1,0,1,1,1,1,1,1,0,1,1,1,1,0,1,1,1,0,0,0,1,1,1
J047_2014,1,1,1,1,0,0,0,1,1,0,0,1,1,1,0,1,0,0,0,0,1,1,1,0,0,1,1,1,1,1,1,1,1,1,0,1,1,0,1,1,1,0,1,0,0,1,1,1,1,0,1,1,1,1,1,0,1,1,1,0,0,1,1,1,0,0,1,0,0,0,0,1,0,1
J048_2014,1,1,1,1,0,0,1,1,1,0,1,0,1,1,0,1,0,0,0,1,1,1,1,1,1,1,1,1,1,1,1,1,1,1,0,1,0,0,1,0,1,0,0,1,0,1,1,1,1,1,1,1,1,1,1,0,1,1,1,0,1,1,1,1,0,0,1,0,0,1,1,1,0,1
J048_2017,1,1,1,1,0,0,1,1,1,0,1,0,1,1,0,1,0,0,0,1,1,1,1,1,1,1,1,1,1,1,1,1,1,1,0,1,0,0,1,0,1,1,0,1,0,0,1,1,1,1,1,1,1,1,1,1,1,1,1,0,1,1,1,1,0,0,1,0,0,1,1,1,0,1
J049_2014,1,1,1,1,0,0,1,1,1,0,0,0,1,1,0,1,1,0,1,1,0,1,0,0,0,0,1,0,0,1,1,1,1,1,0,0,0,0,1,0,0,1,0,1,0,1,1,1,1,1,1,1,0,0,0,1,1,1,1,0,0,1,1,0,0,1,0,1,0,0,0,0,1,1
J051_2014,1,1,1,1,0,0,1,1,1,1,1,0,1,1,1,1,1,0,0,1,1,1,1,0,1,1,0,1,1,1,1,1,1,1,0,1,1,0,1,0,1,0,0,1,0,1,1,1,1,0,1,1,1,1,1,0,1,1,1,0,0,0,1,0,0,1,1,0,0,0,0,0,0,0
J052_2014,1,1,1,1,0,0,1,1,1,0,0,0,1,1,1,1,0,0,1,1,1,1,1,0,1,1,1,1,0,1,1,1,0,1,1,1,0,0,1,0,1,0,0,0,0,0,1,1,1,1,1,1,1,1,1,0,1,1,1,0,0,1,1,1,0,0,1,1,0,1,0,0,1,0
J054_2014,1,1,1,0,0,0,1,1,1,0,0,1,1,1,0,1,1,0,0,1,1,1,1,0,1,1,1,1,0,1,1,1,1,1,0,1,0,0,1,1,1,0,0,0,0,0,1,1,1,0,1,1,1,1,1,0,1,1,1,0,0,1,1,0,1,1,1,0,0,0,0,0,1,0
J058_2014,1,1,1,1,0,0,1,1,1,0,1,1,1,1,1,1,0,0,1,0,1,1,1,1,1,1,0,1,1,1,1,1,1,1,1,1,0,0,1,1,1,0,1,1,0,1,1,1,1,1,1,1,1,1,1,1,1,1,1,0,0,1,1,0,0,1,1,1,0,0,1,1,1,1
J061_2014,1,1,1,1,0,0,1,1,1,1,0,1,1,1,1,1,1,0,0,1,1,1,1,1,1,1,0,1,1,1,1,1,1,1,0,1,0,0,1,0,0,0,0,0,0,1,1,1,1,0,1,1,0,1,1,1,1,1,1,0,1,1,1,0,0,1,1,1,0,0,0,0,1,0
J064_2014,1,1,1,1,0,0,0,1,1,0,0,1,1,1,0,1,1,0,1,1,1,1,1,1,1,1,1,1,1,1,1,1,1,1,0,1,1,1,1,1,0,0,1,0,0,1,1,1,1,1,0,1,1,1,1,1,1,1,1,0,1,1,1,1,0,1,1,0,1,1,0,1,1,1
J065_2014,1,0,1,1,0,0,0,0,0,0,1,1,1,1,0,1,0,0,1,1,1,1,0,1,1,1,0,1,1,1,1,1,1,1,0,1,1,0,1,1,1,1,1,1,0,0,1,1,1,1,1,1,1,1,1,1,1,1,1,1,0,0,1,1,1,0,1,1,0,0,0,0,1,1,1
J066_2014,1,1,1,1,0,0,1,1,1,1,0,1,1,1,0,1,1,0,0,1,1,1,1,1,1,1,1,0,0,1,1,1,1,0,1,0,1,0,0,1,1,1,0,1,0,0,0,1,1,1,0,1,1,0,1,1,0,1,1,1,0,1,0,1,1,0,1,1,0,0,0,0,1,0,1
J068_2014,0,0,1,1,0,0,0,1,1,1,0,1,1,1,1,1,0,0,1,1,0,0,1,0,0,1,1,1,1,1,1,1,1,1,1,0,0,0,1,1,1,1,0,1,0,1,1,1,1,1,1,1,0,1,1,1,1,1,1,0,1,0,1,0,1,0,1,1,0,0,1,1,0,1
J069_2014,0,0,1,1,0,0,0,1,1,0,0,1,1,1,1,1,1,0,1,1,0,1,0,1,1,1,0,1,0,1,1,1,1,1,0,1,0,0,1,1,1,1,1,0,0,1,1,1,1,1,1,1,1,1,1,1,1,1,1,1,1,0,0,1,1,1,0,0,1,0,0,1,1,1,1,1
J070_2014,1,1,1,1,0,0,0,1,1,0,0,1,1,1,0,1,0,0,0,0,1,1,0,1,1,1,1,1,1,1,1,1,1,1,1,0,1,1,0,1,0,1,0,1,0,0,0,1,1,1,1,1,1,0,1,1,0,1,1,1,0,0,1,1,1,0,0,1,0,0,0,0,0,1,1
J078_2014,1,0,1,1,0,0,1,1,1,0,0,1,1,1,1,1,1,0,1,1,1,1,1,0,1,1,0,1,1,1,1,1,1,1,1,1,0,1,1,0,1,1,1,1,0,1,1,1,1,1,1,1,1,1,1,1,0,1,1,1,0,1,1,1,1,1,1,1,0,0,1,1,0,1,0
J082_2014,1,1,1,1,0,0,0,1,1,0,0,1,1,1,0,1,0,0,1,1,0,1,0,1,1,1,0,1,0,1,1,1,1,1,0,1,1,0,1,0,1,0,0,1,0,1,1,1,1,0,1,1,1,1,1,1,1,1,1,0,1,0,1,1,0,1,1,0,0,1,0,0,0,1
J084_2014,1,0,1,1,0,0,1,1,1,0,1,1,1,1,0,1,1,0,1,0,1,1,1,1,1,0,1,1,1,1,1,1,1,1,1,1,0,0,1,0,1,1,0,0,0,0,1,1,1,1,1,1,1,1,1,1,1,1,1,0,1,0,1,1,0,1,1,0,0,1,0,1,1,1
J087_2014,1,1,1,1,0,0,1,1,1,0,0,1,1,1,0,1,0,0,1,1,1,1,0,1,1,1,0,1,1,1,1,1,1,1,0,1,0,0,1,1,1,0,0,1,0,1,1,1,1,1,1,1,1,0,1,1,1,1,0,1,1,1,1,1,1,0,1,1,1,1,1,1,0,1,1
J088_2014,0,0,1,1,0,0,1,1,1,0,0,1,1,1,1,1,1,0,1,1,1,1,1,0,1,1,1,1,0,1,1,1,1,1,1,0,1,0,1,0,1,0,1,0,0,0,1,1,1,1,1,1,0,1,1,1,1,1,1,0,1,1,1,0,0,1,1,1,0,1,0,1,1,1
J088_2017,0,0,1,1,0,0,1,1,1,0,0,1,1,1,1,1,1,0,1,1,1,1,1,0,1,1,1,1,0,1,1,1,1,1,1,0,1,0,1,0,1,0,1,0,0,0,1,1,1,1,1,1,0,1,1,1,1,1,1,0,1,1,1,0,0,1,1,1,0,1,0,1,1,1
J089_2014,1,1,1,1,0,0,1,1,1,1,0,1,1,1,0,1,1,1,0,0,1,1,1,1,0,0,1,1,1,1,1,1,1,1,1,0,1,1,0,1,0,1,0,1,0,0,1,1,1,1,0,1,1,1,1,1,0,1,1,1,0,0,1,1,1,1,1,1,0,0,0,0,0,0,1
J090_2014,1,1,1,1,0,0,1,1,1,0,0,1,1,1,0,1,1,0,1,0,1,1,1,1,0,1,1,1,1,1,1,1,1,1,1,1,1,0,1,1,1,1,1,1,0,0,1,1,1,1,0,1,1,1,1,1,0,1,1,1,0,1,1,1,1,0,0,1,1,0,1,0,0,0,1
J091_2014,1,1,1,1,0,0,1,1,1,1,1,0,1,1,0,1,1,1,0,1,1,1,1,0,1,0,0,0,1,1,1,1,1,1,0,1,0,0,1,0,1,1,1,1,0,1,1,1,1,1,1,1,1,1,1,0,1,1,1,0,1,0,1,0,0,1,1,0,0,0,0,1,0,1
J092_2014,1,1,1,1,0,0,1,1,1,1,0,1,1,1,0,1,1,0,1,1,1,1,1,0,0,1,0,1,1,1,1,1,1,1,0,1,1,0,1,1,1,0,1,1,0,1,1,1,1,1,1,1,1,1,1,0,1,1,1,0,1,0,1,1,0,0,1,0,0,0,1,1,0,1
J101_2014,1,1,1,1,0,0,1,1,1,1,0,1,1,1,0,1,1,0,1,1,0,1,1,1,0,1,0,0,1,1,1,0,1,1,1,1,1,1,1,0,1,1,0,0,0,0,1,1,1,0,1,1,0,0,1,0,1,1,1,0,0,1,1,0,0,1,0,1,0,0,0,1,1,0
J102_2014,1,0,1,1,0,0,1,1,1,1,0,0,1,1,0,1,1,0,1,1,1,1,1,1,0,1,0,0,1,1,1,1,1,1,1,1,1,1,0,0,1,0,0,0,1,1,1,1,0,1,1,0,0,1,0,1,1,1,0,1,1,1,0,1,1,0,0,0,1,0,1,1,1

J103_2014,1,1,1,0,0,0,0,1,1,0,0,1,1,1,0,1,1,1,1,1,0,1,0,1,0,0,1,0,1,1,0,1,1,1,1,1,1,0,1,0,1,0,1,0,0,0,1,1,1,1,1,1,0,0,1,0,1,1,1,0,0,1,1,0,0,1,0,1,0,1,0,1,1,0
J104_2014,1,0,1,0,0,0,1,1,1,1,0,1,1,1,0,1,1,0,0,1,1,1,1,1,1,1,1,1,0,1,1,1,1,1,0,1,1,1,1,0,1,1,1,0,0,1,1,1,1,0,1,1,1,1,1,0,1,1,1,0,0,1,1,1,0,1,1,0,0,1,0,1,1,1
J106_2014,1,0,1,1,0,0,1,1,1,0,0,1,1,1,1,0,1,0,1,1,0,0,1,1,0,0,1,1,1,1,1,1,1,1,1,0,0,0,1,0,1,0,0,1,0,1,1,1,1,0,1,1,0,1,1,1,1,1,1,0,0,1,1,0,0,1,1,0,0,0,0,1,1,1
J108_2014,1,1,1,1,0,0,0,1,1,1,0,1,1,1,1,0,0,1,1,1,0,1,0,1,1,0,1,1,1,1,1,1,1,1,0,0,1,0,1,0,1,0,0,1,0,1,1,1,1,0,1,1,0,1,1,1,1,1,1,0,0,0,1,1,0,1,1,0,0,1,1,0,0,1
J110_2014,1,0,1,1,0,0,0,1,1,0,0,0,1,1,0,1,0,0,1,1,1,1,1,0,0,1,0,0,1,1,1,1,1,1,1,1,0,1,1,1,1,0,0,1,0,1,1,1,1,0,1,1,1,0,1,0,1,1,1,0,1,0,0,1,1,0,0,1,0,1,0,0,1,1
J111_2014,1,1,1,0,0,0,1,1,1,1,0,1,1,1,0,1,1,0,1,1,1,1,0,0,0,0,1,0,1,1,1,1,1,1,0,1,1,1,1,1,1,1,1,1,0,0,1,0,1,1,1,1,0,1,1,1,1,1,1,1,1,1,1,1,0,1,1,0,0,0,1,1,1,1
J111_2017,1,1,1,0,0,0,1,1,1,1,0,1,1,1,0,1,1,0,1,1,1,1,0,0,0,0,1,0,1,1,1,1,1,1,0,1,1,1,1,1,1,1,1,1,0,0,1,0,1,1,1,1,0,1,1,1,1,1,1,1,1,1,1,1,0,1,1,0,0,0,1,1,1,1
J113_2014,1,0,1,1,0,0,1,1,1,0,0,1,1,1,0,1,1,0,1,0,1,1,0,0,0,0,0,0,0,1,1,1,1,1,1,1,0,0,1,0,1,1,0,1,0,0,1,1,1,1,1,1,0,1,1,0,1,1,1,1,0,1,1,0,1,1,0,0,0,0,0,0,0,0
J116_2014,1,1,1,1,0,0,1,1,1,0,0,1,1,1,0,1,0,0,1,0,1,1,1,0,1,1,0,1,1,1,0,1,1,0,0,0,1,0,1,0,0,0,1,1,0,1,1,0,1,0,1,1,0,0,1,0,1,1,1,0,0,1,1,1,0,0,1,0,1,1,0,1,0,0
J117_2014,1,0,1,1,0,0,0,1,0,0,0,1,1,1,1,1,0,0,1,0,0,1,0,0,1,0,1,1,1,1,1,1,1,1,1,0,0,0,1,0,1,1,1,1,0,1,1,1,1,1,1,1,0,0,1,1,1,1,1,0,1,1,1,0,0,1,1,0,1,0,0,1,0,0
J118_2014,1,0,1,0,0,0,1,1,1,0,0,1,1,1,0,1,1,0,1,1,0,0,1,0,0,0,1,0,0,1,1,1,1,0,0,1,1,1,1,1,1,1,1,1,0,1,1,0,1,1,1,1,0,0,0,1,1,1,1,0,1,1,1,0,0,1,0,1,0,0,0,0,1,0
J120_2014,1,0,0,1,0,0,0,1,1,1,0,1,1,1,0,1,1,0,1,1,0,0,1,1,0,1,1,0,0,1,0,1,1,0,1,0,1,0,1,0,1,0,0,1,0,1,1,0,1,1,1,1,0,0,1,1,1,1,1,0,1,1,1,0,0,1,1,1,1,1,1,0,0,1,0
J121_2014,1,1,1,0,0,0,0,1,1,0,0,1,1,1,0,0,0,0,1,1,1,1,0,0,1,1,1,1,0,1,1,1,1,1,0,1,0,1,1,1,0,1,0,1,0,0,1,1,1,1,1,1,0,0,0,1,1,1,1,0,0,1,0,1,0,1,1,1,1,1,1,0,1,1,1
J122_2014,1,1,1,1,0,0,1,1,1,0,0,1,1,1,1,1,0,0,1,1,0,1,1,1,1,1,0,1,1,1,1,1,1,1,1,0,1,1,0,1,0,1,0,1,1,0,1,0,0,1,1,1,1,1,1,1,1,1,1,1,1,1,0,1,0,1,0,1,1,1,0,1,0,0,0,1
J124_2014,0,1,1,0,0,0,0,1,1,0,0,1,1,1,0,0,0,0,1,1,0,0,1,0,1,0,1,0,0,1,0,0,1,0,0,0,0,1,1,0,1,1,1,1,0,1,1,1,1,0,1,1,0,0,0,1,1,1,1,0,0,1,1,0,0,1,0,1,0,1,1,1,1,1
J129_2014,1,1,1,1,0,0,0,1,1,0,1,1,1,1,0,1,1,0,1,0,0,1,0,1,0,0,1,0,1,1,1,1,1,1,1,1,1,1,1,1,1,1,0,1,0,1,1,1,1,0,1,1,0,1,1,0,1,1,1,0,0,1,1,0,0,1,0,1,0,1,1,0,1,0
J132_2014,1,0,1,1,0,0,0,1,1,0,0,1,1,1,0,1,0,0,1,1,0,1,1,1,1,0,1,0,1,1,1,0,1,1,1,1,0,1,1,0,1,0,1,0,0,1,1,0,1,1,1,1,0,1,0,1,1,1,1,0,1,1,0,0,0,1,0,0,1,0,0,1,1,1
J133_2014,1,0,1,0,0,0,0,1,1,0,0,1,1,1,1,1,1,0,1,1,0,0,1,1,1,0,1,0,1,1,1,1,1,1,1,1,1,1,1,0,0,1,0,0,0,1,1,1,1,1,1,1,0,0,0,1,1,1,1,0,0,0,1,1,0,1,0,0,1,1,0,1,1,1
J134_2016,1,0,1,1,0,0,0,1,1,0,0,1,1,1,0,1,0,0,1,1,1,1,1,1,1,1,1,1,0,1,1,1,1,1,0,0,0,0,1,0,1,0,0,1,0,1,1,1,1,1,1,1,1,1,1,1,1,1,1,1,0,1,1,1,1,0,1,1,0,0,1,1,1,1,1
J135_2014,1,1,1,1,0,0,1,0,0,0,1,1,1,1,0,1,1,0,1,1,0,1,1,1,1,1,1,1,0,1,1,1,1,1,1,1,1,0,1,1,1,0,1,0,0,1,0,1,1,0,1,0,1,1,1,0,0,1,1,0,1,0,1,1,0,1,0,1,1,1,1,0,0,0,1,1
J144_2014,1,0,1,1,0,0,1,1,1,1,1,1,1,1,0,1,0,0,0,1,1,1,0,1,0,1,1,1,1,1,1,1,1,1,1,0,1,0,0,1,0,1,1,1,1,0,1,1,0,1,1,1,1,0,1,1,0,1,1,1,0,1,0,1,0,0,1,1,0,0,1,0,1,1,1
J147_2014,1,0,1,1,0,0,1,1,1,0,0,0,1,1,1,1,1,0,1,1,1,1,1,0,1,0,1,1,0,1,1,0,0,1,0,1,0,0,1,0,1,0,1,0,0,1,1,1,1,0,1,1,1,1,1,0,1,1,1,0,1,1,1,1,0,0,1,1,0,0,0,0,0,1
K025_2015,0,1,1,1,0,0,0,1,1,0,1,0,1,1,0,1,1,1,0,0,0,1,0,1,0,0,1,1,1,1,1,1,1,1,1,0,1,0,1,1,1,1,0,0,0,0,1,0,1,0,1,1,1,1,1,1,1,1,1,0,1,1,1,0,0,1,1,0,0,1,1,0,1,0
K027_2015,0,1,1,1,0,0,0,0,0,0,0,1,1,1,0,1,1,0,1,0,0,1,0,0,1,1,0,1,1,1,1,1,1,1,1,0,1,0,0,1,0,1,0,0,0,0,0,1,1,1,0,1,1,0,1,1,1,1,1,1,0,1,0,1,1,0,1,1,1,0,1,0,1,1,1
K029_2015,1,1,1,1,0,0,0,0,0,0,0,1,1,1,0,1,1,0,1,1,1,1,1,1,1,1,0,1,1,1,1,1,1,1,0,1,1,0,1,0,0,1,0,0,0,0,1,1,1,0,1,1,0,1,1,1,1,1,1,0,1,0,1,0,0,1,1,1,0,0,0,0,1,1
K034_2015,1,1,1,1,0,0,1,1,1,0,1,1,0,1,1,1,0,0,1,1,1,1,0,0,1,1,1,1,1,1,1,1,0,1,1,0,0,0,0,1,0,1,0,1,0,0,1,1,1,1,0,1,1,1,1,0,1,1,1,1,0,1,1,1,1,0,1,1,0,1,0,0,0,0,0
K035_2015,1,1,1,1,0,0,1,1,1,0,0,1,1,1,1,1,0,0,1,1,0,1,1,0,1,1,0,1,1,1,1,1,0,1,0,1,0,0,1,0,1,1,0,1,0,1,1,0,1,0,0,1,1,1,1,1,1,1,1,0,1,1,1,1,0,1,1,1,0,1,0,1,1,1
K037_2015,1,1,1,1,0,0,0,1,1,0,1,1,1,1,0,1,1,0,1,1,0,1,0,0,1,1,0,1,1,1,1,1,1,1,0,1,0,0,1,1,1,0,1,1,0,0,1,1,1,1,1,1,0,1,1,1,1,1,1,0,0,1,0,1,0,1,1,0,0,1,0,1,0,0
K040_2015,1,1,1,1,1,0,1,1,1,0,0,1,1,1,1,1,1,0,0,1,1,1,1,1,1,1,0,1,1,1,1,1,1,1,1,1,1,1,0,1,0,1,0,1,1,0,0,1,1,1,0,1,1,1,1,1,1,1,1,1,1,0,1,1,1,0,0,1,1,0,0,1,1,1,1,1
K046_2015,1,1,1,1,0,0,1,1,1,1,0,0,1,1,0,1,0,0,0,1,1,1,0,1,0,1,0,1,1,1,1,1,1,1,1,0,1,1,0,1,0,1,0,0,1,0,0,1,1,1,1,1,1,0,1,1,1,1,1,1,1,1,1,1,1,0,1,1,0,0,0,1,0,0,0
K050_2015,1,0,1,1,0,0,1,1,1,0,0,0,0,1,0,1,0,0,0,0,1,0,0,0,0,1,0,1,1,1,0,0,0,1,1,0,0,0,1,0,1,1,1,0,0,1,1,0,1,1,1,1,0,1,1,0,1,1,1,0,1,1,1,1,0,1,1,0,1,0,0,1,1,1
K051_2015,1,0,1,1,0,0,1,1,1,0,0,1,1,1,0,1,1,0,1,1,1,1,1,0,1,1,1,1,1,1,1,1,1,1,1,1,0,1,1,1,1,1,1,1,1,0,0,1,1,1,1,1,1,1,1,1,1,1,1,1,1,1,0,0,0,1,0,0,1,1,0,1,0,0,0,0,0
K053_2015,1,1,1,0,0,0,0,1,1,0,1,1,1,1,1,1,1,0,1,1,0,1,0,0,0,0,0,0,1,1,1,1,1,1,1,1,0,1,0,1,0,1,0,1,1,0,0,1,1,1,1,1,1,1,1,1,1,1,1,1,1,0,1,1,1,1,0,1,1,0,0,1,0,1,0,1
K058_2015,1,1,1,1,0,0,0,1,1,0,0,0,1,1,0,1,0,0,1,0,1,1,0,1,1,1,1,1,1,1,1,1,1,1,1,0,1,1,0,1,0,1,0,1,1,0,1,1,1,1,1,1,1,1,1,0,1,1,1,1,1,0,1,0,1,1,0,1,1,0,1,0,1,1,0,1
K059_2015,1,1,1,1,0,0,1,1,0,0,0,1,0,1,0,1,0,0,1,1,1,1,1,0,1,0,1,1,1,1,1,0,1,1,1,0,0,1,1,0,1,1,1,0,0,1,1,1,1,1,0,1,1,1,1,1,1,1,1,1,0,0,0,1,1,0,1,1,0,0,0,1,0,0,0
K060_2015,1,0,1,1,0,0,0,1,1,0,0,1,1,1,0,1,1,0,1,1,0,1,1,1,0,0,1,1,1,1,1,0,1,1,0,0,0,0,1,1,1,0,0,0,0,1,1,1,1,0,1,1,1,1,1,1,1,1,1,1,0,1,0,1,1,0,1,1,1,1,1,0,0,1,1,1
K061_2015,1,1,0,1,0,1,1,1,1,1,0,1,0,0,0,1,0,0,0,1,1,1,0,0,0,1,1,1,0,1,1,0,1,0,0,1,0,0,0,1,0,0,0,1,0,0,1,0,1,1,1,1,0,0,1,1,1,1,1,0,0,0,1,0,0,1,0,0,0,1,0,0,0,0
K062_2015,1,1,1,1,0,0,1,1,1,0,1,1,1,1,0,1,1,0,1,0,1,1,1,1,1,1,0,1,1,1,1,1,1,1,0,1,1,0,1,1,1,0,1,0,0,1,1,1,1,1,1,1,1,1,1,1,1,1,1,1,0,1,1,1,1,0,1,1,1,0,0,0,1,1,1
K063_2015,0,1,1,1,0,0,0,1,0,0,0,0,0,1,0,1,0,0,1,0,0,1,1,0,0,0,1,1,1,1,1,0,1,0,0,1,0,0,1,0,0,0,0,1,0,1,1,0,1,1,0,0,1,1,0,1,0,1,1,0,1,0,1,0,0,0,1,0,0,0,0,0,0,0
K064_2015,1,1,1,0,0,0,0,1,1,0,0,1,1,1,0,1,1,0,0,1,1,0,1,1,1,1,0,1,0,1,1,1,1,1,1,0,1,0,1,0,1,1,0,0,0,0,1,1,1,1,1,1,0,1,1,1,1,1,1,0,0,1,0,1,0,1,1,1,0,1,0,1,1,0
L002_2016,1,1,0,1,0,0,1,1,1,0,0,1,1,1,0,1,0,0,1,0,1,0,1,0,1,1,1,1,1,1,1,1,1,1,0,0,0,0,1,0,1,1,0,1,0,1,1,1,1,0,1,1,0,1,1,1,1,1,1,0,1,0,0,1,1,1,1,0,0,0,1,1,1,1
L003_2016,1,0,1,1,0,0,1,1,1,0,0,1,1,1,0,1,1,0,1,0,1,1,1,1,1,0,1,1,1,1,1,1,1,1,0,0,0,0,1,0,1,0,0,0,0,0,1,1,1,0,1,1,0,1,1,1,1,1,1,0,1,0,1,1,0,1,1,0,0,1,1,0,0,1
L003_2017,1,0,1,1,0,0,1,1,1,0,0,1,1,1,0,1,1,0,1,0,1,1,1,1,1,0,1,1,1,1,1,1,1,1,0,0,0,0,1,0,1,0,0,0,0,0,1,1,1,0,1,1,0,1,1,1,1,1,1,0,1,0,1,1,0,1,1,0,0,1,1,0,0,1
L004_2016,1,1,1,1,0,0,1,1,1,0,1,1,1,1,1,1,1,0,1,1,1,1,1,1,1,1,1,1,1,1,1,1,1,1,0,1,1,0,1,0,1,0,1,0,0,1,1,1,1,0,1,1,0,1,1,1,1,0,1,0,1,1,0,1,0,1,1,1,0,1,1,0,1,1
L004_2017,1,1,1,1,0,0,1,1,1,0,1,1,1,1,1,1,1,0,1,1,1,1,1,1,1,1,1,1,1,1,1,1,1,1,0,1,1,0,1,0,1,0,1,0,0,1,1,1,1,0,1,1,0,1,1,1,1,0,1,0,1,1,0,1,0,1,1,1,0,1,1,0,1,1
L005_2016,1,0,1,0,0,0,1,1,1,0,0,1,1,1,0,1,1,0,1,1,1,0,1,1,1,1,1,1,1,1,1,1,1,1,1,1,0,0,1,0,1,0,1,1,0,0,1,1,1,1,1,1,1,1,1,1,1,1,1,1,0,1,1,0,0,0,1,1,0,0,1,1,1,1,0
L005_2017,1,0,1,0,0,0,1,1,1,0,0,1,1,1,0,1,1,0,1,1,1,0,1,1,1,1,1,1,1,1,1,1,1,1,1,1,0,0,1,0,1,0,1,1,0,0,1,1,1,1,1,1,1,1,1,1,1,1,1,1,0,1,1,0,0,0,1,1,0,0,1,1,1,1,0
L006_2016,1,0,1,1,0,0,1,1,1,0,0,1,1,1,1,1,1,1,0,1,1,1,1,0,1,1,0,0,1,0,1,1,1,1,1,0,1,1,0,1,0,1,1,1,0,0,0,1,1,1,1,1,1,1,1,0,1,1,1,1,0,1,1,1,0,0,1,1,0,0,0,0,0,1,0
L009_2016,1,0,1,1,0,0,1,1,1,1,0,1,1,1,0,1,1,0,1,1,0,1,1,1,1,0,0,1,0,1,1,1,1,1,0,1,0,0,1,1,0,0,1,1,0,1,1,1,1,1,0,1,1,1,1,1,1,1,1,0,1,1,1,1,0,1,1,0,0,1,0,1,1,0
L009_2017,1,0,1,1,0,0,1,1,1,1,0,1,1,1,0,1,1,0,1,1,0,1,1,1,1,0,0,1,0,1,1,1,1,1,0,1,0,0,1,1,0,0,1,1,0,1,1,1,1,1,0,1,1,1,1,1,1,1,1,0,1,1,1,1,0,1,1,0,0,1,0,1,1,0
L010_2016,1,1,1,0,0,0,1,1,1,0,0,1,1,1,0,1,1,0,1,1,0,1,1,1,1,1,1,1,1,1,1,1,1,1,1,1,0,0,1,0,1,1,0,1,0,0,1,1,0,0,1,1,0,0,1,1,1,1,1,0,1,1,0,0,1,1,1,0,0,1,0,1,1,1
L011_2016,1,0,1,1,0,0,1,1,1,1,0,1,1,1,1,1,0,0,1,1,0,1,1,1,1,0,1,1,0,1,1,1,1,1,1,1,1,1,0,1,1,1,0,0,1,0,0,1,1,1,1,1,1,1,1,1,1,1,1,1,0,1,1,1,1,1,1,1,0,0,1,1,1,1,1
L011_2017,1,0,1,1,0,0,1,1,1,1,0,1,1,1,1,1,0,0,1,1,0,1,1,1,1,0,1,1,0,1,1,1,1,1,1,1,1,1,0,1,1,1,0,0,1,0,0,1,1,1,1,1,1,1,1,1,1,1,1,1,0,1,1,1,1,1,1,1,0,0,1,1,1,1,1
L013_2016,1,0,1,0,0,0,1,1,1,0,0,1,1,1,0,1,1,0,1,1,1,1,0,0,1,1,0,1,1,1,1,1,1,1,1,1,1,1,1,1,0,1,1,1,0,0,0,1,1,1,1,1,1,1,0,1,1,1,1,1,0,1,1,1,1,0,1,1,1,0,0,1,1,1,1
L014_2016,1,1,1,1,0,0,1,1,1,0,0,1,1,1,1,1,1,0,1,0,1,1,0,1,1,1,1,1,1,1,1,1,1,1,1,1,0,0,1,0,1,0,1,0,0,1,1,1,1,1,0,1,1,1,1,1,1,1,1,1,1,0,1,1,0,0,1,1,0,1,1,0,1,1

L014_2017,1,1,1,1,0,0,1,1,1,0,0,1,1,1,1,1,1,0,1,0,1,1,0,1,1,1,1,1,1,1,1,1,1,1,1,1,0,0,1,0,1,0,1,0,0,1,1,1,1,1,0,1,1,1,1,1,1,1,1,1,1,0,1,1,0,0,1,1,0,1,1,0,1,1
L015_2016,1,1,1,1,0,0,0,1,1,1,0,1,1,1,1,1,1,0,1,1,1,1,0,0,1,1,0,1,1,1,1,1,1,1,0,0,1,1,1,0,1,0,0,1,0,0,1,1,1,1,1,1,1,1,1,1,1,0,1,0,0,1,1,1,0,0,1,1,0,0,0,0,1,1
L016_2016,1,1,1,1,0,0,1,1,1,1,0,1,1,1,1,1,1,0,1,1,1,1,1,1,1,0,1,1,1,1,1,1,1,1,0,1,0,0,1,0,1,1,1,0,0,0,0,1,1,1,1,1,0,1,1,1,1,1,1,0,1,1,0,1,0,0,1,0,0,1,1,1,1,1
L017_2016,1,0,1,1,0,0,0,1,1,1,0,1,1,1,0,1,1,1,0,1,1,1,0,1,1,0,0,1,1,1,1,1,1,1,1,1,0,0,1,0,1,1,1,1,0,1,1,1,1,1,1,1,0,1,1,1,1,1,1,0,0,1,0,1,1,1,1,0,0,1,0,0,1,1
L017_2017,1,0,1,1,0,0,0,1,1,1,0,1,1,1,0,1,1,1,0,1,1,1,0,1,1,0,0,1,1,1,1,1,1,1,1,1,0,0,1,0,1,1,1,1,0,1,1,1,1,1,1,1,0,1,1,1,1,1,1,0,0,1,0,1,1,1,1,0,0,1,0,0,1,1
L020_2016,1,1,1,1,0,0,1,1,1,0,0,1,1,1,1,1,1,0,1,1,1,0,1,1,1,1,1,1,1,1,1,1,1,1,0,1,0,0,1,0,1,1,1,0,0,0,1,1,1,1,1,1,1,1,1,1,1,0,1,0,1,1,1,1,0,0,1,1,0,1,0,0,1,1
L020_2017,1,1,1,1,0,0,1,1,1,0,0,1,1,1,1,1,1,0,1,1,1,0,1,1,1,1,1,1,1,1,1,1,1,1,0,1,0,0,1,0,1,1,1,0,0,0,1,1,1,1,1,1,1,1,1,1,1,0,1,0,1,1,1,1,0,0,1,1,0,1,0,0,1,1
L027_2016,0,0,1,1,0,0,0,1,1,0,0,1,1,1,0,1,1,0,1,0,0,1,0,0,1,1,0,0,1,1,1,1,1,1,1,1,1,0,1,0,1,1,0,0,0,1,1,1,1,1,1,1,1,1,1,1,1,1,1,0,0,1,1,1,0,1,1,0,1,0,0,1,1,1
L035_2016,1,1,1,1,0,0,0,1,1,0,0,1,1,1,0,1,1,0,1,1,1,1,0,1,1,0,0,1,1,1,1,1,1,1,0,1,1,0,1,1,0,0,0,1,0,0,1,1,1,1,1,1,1,1,1,1,1,1,1,1,1,1,1,1,0,1,1,1,0,1,0,1,1,1
L035_2017,1,1,1,1,0,0,0,1,1,0,1,1,1,1,0,1,1,0,1,1,1,1,0,1,1,0,0,1,1,1,1,1,1,1,0,1,1,0,1,1,0,0,0,1,0,0,1,1,1,1,1,1,1,1,1,1,1,1,1,1,1,1,1,1,0,1,1,1,0,1,0,1,1,1
L037_2016,1,1,1,1,0,0,1,1,1,0,0,1,1,1,0,1,1,0,1,1,1,0,0,1,1,1,0,1,1,1,1,1,1,1,0,1,0,0,1,0,1,0,0,0,0,0,1,1,1,0,1,1,0,1,1,0,1,1,1,0,1,1,0,1,0,0,1,0,0,0,0,1,1,1
L039_2016,1,1,1,1,0,0,1,1,0,0,0,1,1,1,1,1,1,0,1,1,1,1,0,1,0,1,1,1,1,1,1,1,1,1,0,1,1,1,1,0,1,0,0,0,0,0,1,1,1,0,1,1,1,1,1,1,1,1,1,0,1,1,0,1,1,1,1,0,0,1,0,1,0,1
L039_2017,1,1,1,1,0,0,1,1,0,0,0,1,1,1,1,1,1,0,1,1,1,1,0,1,0,1,1,1,1,1,1,1,1,1,0,1,1,1,1,0,1,0,0,0,0,0,1,1,1,0,1,1,1,1,1,1,1,1,1,0,1,1,0,1,1,1,1,0,0,1,0,1,0,1
L043_2016,1,0,1,1,0,0,1,1,1,0,1,1,1,1,0,1,1,0,1,1,1,1,0,0,1,1,1,1,1,1,1,1,0,1,0,1,1,0,1,0,1,1,1,1,0,0,1,1,1,1,1,1,1,1,1,1,1,1,1,0,1,1,0,0,0,1,1,1,1,0,0,0,1,0
L043_2017,1,0,1,1,0,0,1,1,1,0,1,1,1,1,0,1,1,0,1,1,1,1,0,0,1,1,1,1,1,1,1,1,0,1,0,1,1,0,1,0,1,1,1,1,0,0,1,1,1,1,1,1,1,1,1,1,1,1,1,0,1,1,0,0,0,1,1,1,1,0,0,0,1,0
L048_2016,1,0,1,1,0,0,1,1,1,0,0,1,1,1,0,1,0,0,1,0,1,1,0,1,1,0,1,1,0,1,1,1,1,1,0,1,1,0,1,1,1,1,0,1,0,0,1,1,1,1,0,1,0,1,1,1,1,1,1,0,0,0,1,1,0,1,1,0,0,1,1,1,0,1
L056_2016,1,1,1,1,0,0,1,1,1,0,0,0,1,1,0,1,1,0,0,0,1,1,1,0,0,0,0,1,1,1,1,1,1,1,1,0,1,0,1,1,0,1,0,1,1,1,1,1,1,0,1,1,1,0,1,1,1,1,1,0,1,1,1,0,0,1,1,1,1,0,0,1,1,0
L060_2016,1,1,1,1,0,0,1,1,1,0,0,1,1,1,0,1,1,0,1,0,1,1,0,0,0,0,1,1,1,1,1,1,1,1,0,1,0,0,1,0,1,0,1,0,0,0,1,1,1,1,1,1,1,1,1,1,1,1,1,0,1,1,1,1,1,1,1,0,0,1,0,1,1,1
L060_2017,1,1,1,1,0,0,1,1,1,0,0,1,1,1,0,1,1,0,1,0,1,1,0,0,0,0,1,1,1,1,1,1,1,1,0,1,0,0,1,0,1,0,1,0,0,0,1,1,1,1,1,1,1,1,1,1,1,1,1,0,1,1,1,1,1,1,1,0,0,1,0,1,1,1
L087_2016,1,0,1,1,0,0,0,1,1,0,0,1,1,1,1,1,1,0,1,1,1,1,0,1,1,1,0,1,1,1,1,1,1,1,1,1,1,1,1,1,1,1,0,0,0,0,1,0,1,1,1,1,0,1,1,1,1,1,1,0,1,1,1,1,0,1,1,0,0,1,0,1,1,1
L087_2017,1,0,1,1,0,0,0,1,1,0,0,1,1,1,1,1,1,0,1,1,1,1,0,1,1,1,0,1,1,1,1,1,1,1,1,1,1,1,1,1,1,1,0,0,0,0,1,0,1,1,1,1,0,1,1,1,1,1,1,0,1,1,1,1,0,1,1,0,0,1,0,1,1,1
L090_2016,1,0,1,1,0,0,1,1,0,1,0,1,1,1,1,1,1,0,1,1,1,1,0,0,0,1,1,1,1,1,1,1,1,1,0,1,0,0,1,0,1,1,0,1,0,0,1,0,1,1,1,1,1,1,1,1,1,1,1,0,1,1,0,1,0,0,1,1,0,0,0,0,1,1
L090_2017,1,0,1,1,0,0,1,1,0,1,0,1,1,1,1,1,1,0,1,1,1,1,0,0,0,1,1,1,1,1,1,1,1,1,0,1,0,0,1,0,1,1,0,1,0,0,1,0,1,1,1,1,1,1,1,1,1,1,1,0,1,1,0,1,0,0,1,1,0,0,0,0,1,1
L092_2016,0,1,0,1,0,0,1,1,1,0,1,1,0,1,0,1,1,0,1,0,1,0,1,1,1,1,0,1,0,1,1,1,1,1,1,1,1,0,1,0,1,0,0,1,0,0,1,1,1,0,1,0,1,1,1,1,1,1,1,0,1,0,1,1,0,1,1,0,0,1,0,0,1,1
L097_2016,0,1,1,1,0,0,1,1,1,0,0,1,1,1,0,1,1,0,1,1,0,1,1,1,1,0,1,1,1,1,1,1,1,1,0,1,1,1,0,1,0,1,1,0,0,0,1,1,1,1,1,1,0,1,0,1,0,1,1,0,1,1,1,1,0,1,1,0,0,1,1,1,1,1
L099_2016,1,0,1,1,0,0,1,0,0,0,0,1,1,1,0,1,1,0,1,1,0,1,1,1,1,1,1,1,1,1,1,1,0,1,0,0,0,0,1,1,1,1,0,1,0,0,1,1,1,1,0,1,1,1,1,1,1,1,1,0,1,0,1,1,0,1,1,0,0,1,0,0,1,0
L102_2016,1,1,1,1,0,0,1,1,1,0,0,1,1,1,1,1,1,0,1,1,1,1,0,1,1,1,0,1,1,1,1,1,1,1,1,1,0,0,1,0,1,1,1,0,0,0,1,1,1,0,1,1,1,1,1,1,1,1,1,0,0,0,0,0,0,1,1,0,0,1,0,1,0,0
L103_2016,0,0,1,1,0,0,0,1,1,0,1,1,1,1,1,1,0,0,1,1,1,1,1,0,0,1,0,1,1,1,1,1,1,1,0,1,0,0,1,0,1,1,0,0,0,1,1,1,1,0,1,1,1,1,1,1,1,1,1,0,1,1,0,0,0,1,1,0,0,1,0,1,1,1

**# 2) Epigenetic profiles from MSAP analysis:** loci from *HpaII* treatment, not associated with polymorphic loci within the *MspI* treatment. For each individual, ID and sampling year are provide.

Ind_year,Sel1-495,Sel1-485,Sel1-470,Sel1-411,Sel1-350,Sel1-344,Sel1-342,Sel1-317,Sel1-312,Sel1-304,Sel1-298,Sel1-295,Sel1-270,Sel1-268,Sel1-264,Sel1-257,Sel1-216,Sel1-209,Sel1-207,Sel1-205,Sel1-194,Sel1-191,Sel1-190,Sel1-182,Sel1-170,Sel1-168,Sel1-163,Sel1-159,Sel1-153,Sel1-146,Sel1-113,Sel1-112,Sel2-420,Sel2-411,Sel2-400,Sel2-308,Sel2-300,Sel2-290,Sel2-283,Sel2-275,Sel2-268,Sel2-236,Sel2-216,Sel2-188,Sel2-185,Sel2-183,Sel2-170,Sel2-160,Sel2-147,Sel2-131,Sel2-129,Sel3-432,Sel3-405,Sel3-388,Sel3-365,Sel3-352,Sel3-315,Sel3-271,Sel3-207,Sel3-203,Sel3-188,Sel3-168,Sel3-165,Sel3-164,Sel3-161,Sel3-160,Sel3-159,Sel3-158,Sel3-148,Sel3-106,Sel4-400,Sel4-397,Sel4-350,Sel4-333,Sel4-316,Sel4-287,Sel4-281,Sel4-275,Sel4-267,Sel4-258,Sel4-246,Sel4-245,Sel4-236,Sel4-217,Sel4-205,Sel4-188,Sel4-177,Sel4-176,Sel4-150,Sel4-146,Sel4-126,Sel4-120,Sel4-113,Sel4-112,Sel4-111,Sel4-110,Sel5-370,Sel5-200,Sel5-192,Sel5-190,Sel5-175,Sel5-170,Sel5-162,Sel5-160,Sel5-150,Sel5-148,Sel5-142,Sel5-130,Sel5-129
F023_2013,1,0,1,0,0,1,1,0,1,1,1,0,1,0,0,0,0,1,0,1,1,0,0,0,1,0,0,1,1,0,1,1,0,0,0,0,0,0,1,1,1,1,0,1,1,0,1,1,0,1,1,0,1,1,0,0,0,0,1,0,1,0,1,0,1,1,1,0,1,1,0,1,1,0,1,1,1,0,0,0,0,1,0,0,0,1,0,1,0,1,1,1,1,1,1,0,0,0,0,1,1,0,0,0,0,1,0,1,1
H002_2013,1,0,1,0,1,1,0,1,1,1,1,0,1,0,0,1,0,0,0,0,1,0,0,0,0,0,1,1,1,0,1,1,0,1,0,1,1,0,1,1,1,1,0,0,1,0,0,1,0,0,0,1,1,1,0,1,1,1,1,1,1,0,1,0,1,1,1,1,0,1,0,1,1,1,1,0,0,0,0,0,1,1,0,0,1,1,1,0,0,0,1,1,0,0,0,0,0,0,0,1,1,0,0,0,1,1,1,1,1
H004_2013,1,0,1,0,1,1,0,1,1,1,1,0,0,1,0,1,1,1,1,1,1,1,1,0,1,0,1,1,1,0,1,1,0,0,1,1,0,0,1,0,1,1,1,1,1,0,1,0,1,1,1,1,1,1,0,0,1,0,1,1,1,0,1,0,1,1,1,1,1,1,1,1,1,0,1,1,1,0,0,0,0,1,0,0,0,1,1,1,0,1,1,1,1,1,1,0,0,0,1,1,1,0,0,0,0,0,1,1,1,1
H007_2013,1,0,1,0,1,1,0,1,1,1,1,0,0,0,1,0,1,1,1,1,1,1,0,0,1,0,1,1,1,0,1,1,0,1,0,1,0,0,0,1,1,1,0,0,1,0,0,0,0,1,1,1,1,1,0,0,1,1,1,1,1,0,0,0,1,1,1,0,1,1,0,1,1,1,1,1,0,0,0,0,0,1,1,0,1,0,1,0,0,0,1,1,0,0,0,0,0,0,0,1,1,0,0,0,0,1,0,1,1
H024_2013,0,1,1,0,0,1,0,1,1,0,0,0,0,0,0,1,1,0,0,0,0,0,1,1,1,0,0,1,1,1,0,0,0,0,0,1,0,0,0,0,1,1,1,0,1,1,1,0,1,1,1,0,1,1,0,1,0,1,0,0,1,0,0,0,0,0,1,0,0,1,0,0,1,0,1,0,0,0,0,0,0,1,0,0,1,1,1,1,0,0,1,1,1,1,1,0,0,0,1,1,0,0,0,1,0,1,0,1,1
H042_2013,1,1,1,0,0,1,0,1,1,0,1,0,1,0,1,1,1,1,1,1,1,1,0,1,1,0,1,1,1,1,1,1,0,0,1,1,1,0,0,0,1,1,1,1,1,1,0,1,1,1,1,1,0,1,0,0,0,1,1,0,1,0,1,0,1,0,1,0,0,1,0,1,0,1,1,0,0,0,0,0,0,1,0,0,0,1,0,1,0,1,1,1,1,0,0,0,1,0,1,1,1,0,0,1,0,1,0,1,1
H056_2013,1,0,1,1,1,1,1,1,1,1,0,1,1,0,1,1,1,1,1,1,0,1,1,0,1,0,0,1,1,0,1,1,1,1,1,1,0,0,0,1,1,1,1,1,1,1,1,1,1,1,0,1,1,1,1,0,1,1,0,1,1,1,0,1,0,1,1,1,1,1,1,0,0,1,0,1,0,1,0,0,0,0,1,1,0,1,1,1,0,0,1,1,1,1,1,1,1,0,1,1,0,1,1,0,0,0,0,1,0,1,1
H058_2013,0,0,1,0,1,0,1,1,1,0,0,0,0,0,1,1,1,0,0,1,1,1,1,1,1,0,0,1,1,1,1,1,0,1,1,1,0,0,1,0,1,1,1,1,1,1,1,1,1,1,1,1,1,1,0,0,1,0,1,1,1,1,1,0,1,0,1,1,1,1,0,0,0,0,1,0,1,0,0,0,1,1,0,0,0,1,1,1,0,1,1,1,1,1,1,1,1,1,1,1,1,1,1,0,1,1,1,1,1,1
H062_2013,1,0,1,0,0,1,0,0,0,1,1,1,0,1,0,0,0,0,0,1,1,1,1,1,1,0,0,1,1,0,1,1,0,1,1,1,0,0,0,1,1,1,1,1,1,0,0,1,1,1,1,0,1,1,0,1,1,0,0,1,1,1,0,0,1,1,1,1,0,1,0,1,1,1,1,0,1,0,0,0,1,1,0,0,0,1,0,1,0,1,1,0,0,0,0,0,1,0,1,1,1,0,0,1,0,1,1,1,0
H063_2013,1,0,1,0,0,1,0,0,1,1,1,0,1,1,0,1,1,1,1,1,1,0,0,1,1,0,0,1,1,0,1,0,0,1,1,1,1,0,0,0,1,0,1,1,0,1,1,1,1,1,1,1,1,1,1,0,1,1,0,1,1,1,0,0,1,1,0,1,0,1,1,1,1,1,0,1,0,1,0,1,0,0,1,0,0,0,1,1,1,0,1,1,1,1,1,1,1,0,1,0,1,1,1,0,0,1,1,1,1,1,1
H065_2013,0,0,1,0,0,1,0,1,1,0,1,0,1,0,0,0,0,0,0,1,1,1,0,0,1,0,0,1,1,1,1,0,0,0,0,1,1,0,0,1,1,1,1,1,0,0,1,0,1,1,0,1,1,1,0,0,0,0,1,1,1,0,0,0,0,1,1,1,0,1,0,0,1,0,1,0,1,0,0,0,0,0,0,0,1,1,1,0,0,1,1,1,1,1,1,1,0,1,1,0,1,1,1,0,0,1,0,1,1,1
H068_2013,0,0,1,0,1,1,0,1,1,1,1,0,0,1,0,1,1,0,0,1,1,0,0,1,1,0,1,0,1,1,1,1,0,1,1,1,0,0,1,1,1,1,1,1,1,1,0,1,0,0,1,1,1,1,1,0,1,0,0,1,1,1,0,1,0,1,1,1,1,1,1,1,1,0,1,0,1,0,1,0,0,0,0,1,0,0,1,1,1,1,0,1,1,1,1,1,1,1,0,1,1,1,1,1,1,1,1,0,1,1,1,1,0
H073_2013,0,0,1,0,0,1,1,0,1,1,0,0,0,1,0,1,1,1,1,1,1,0,0,1,1,0,1,1,1,1,1,1,0,0,1,1,1,1,0,0,1,1,1,1,1,1,0,0,1,1,1,1,1,1,0,1,0,1,1,1,1,1,1,1,0,0,0,1,1,1,0,1,1,0,0,1,0,1,0,1,0,0,0,0,1,0,0,1,1,1,1,0,1,1,1,1,1,0,0,1,1,1,1,1,1,1,0,0,1,1,1,1,1
I001_2013,1,1,1,1,1,1,1,1,1,1,0,1,0,1,0,1,1,0,1,1,1,1,1,1,0,0,1,1,1,0,1,1,1,1,0,1,1,0,1,1,1,0,1,1,0,0,1,1,1,1,0,1,1,1,1,1,1,1,1,1,1,1,1,1,1,1,1,1,1,1,1,0,0,0,1,0,1,0,0,0,1,0,0,0,0,1,1,1,0,1,1,1,1,1,1,1,0,0,1,1,0,1,1,0,1,0,1,1,1,1
I002_2013,0,0,0,1,1,0,1,0,0,0,1,1,1,1,1,1,1,0,1,1,1,0,1,1,0,0,1,1,1,1,0,0,0,0,1,1,1,0,1,1,1,1,1,1,1,1,0,1,1,1,1,1,0,1,1,0,0,0,0,0,1,1,0,1,1,1,0,1,1,1,1,1,0,1,0,1,0,1,0,0,0,0,1,0,0,1,1,1,0,0,1,1,1,1,1,1,1,1,1,0,0,1,1,0,1,0,1,1,1,1
I003_2013,0,0,1,0,0,0,1,0,1,1,1,0,1,0,0,0,1,1,1,1,1,0,0,0,1,0,0,1,1,0,1,1,0,0,0,1,0,0,0,0,1,1,1,1,1,0,1,1,0,1,1,1,1,0,0,0,0,0,1,1,1,0,1,0,1,1,1,1,1,1,0,0,0,0,1,1,1,0,0,0,0,0,0,0,1,1,0,1,0,0,1,1,1,1,1,0,0,0,0,1,1,1,0,0,0,1,1,1,1
I004_2013,1,1,1,1,1,1,0,1,1,0,1,1,0,1,1,1,1,1,1,1,1,1,1,0,1,0,0,0,1,1,1,0,0,0,1,1,1,1,0,1,1,1,1,1,1,1,1,0,1,1,1,1,1,0,1,1,0,0,0,0,0,0,1,0,1,1,0,0,1,0,1,1,1,0,0,0,1,0,1,0,0,0,1,1,0,0,1,1,1,0,0,1,1,1,0,0,0,0,0,0,1,1,1,1,1,1,1,0,1,0,1,1
I006_2013,0,0,1,1,1,1,0,0,1,1,1,0,0,0,0,1,0,1,0,1,1,1,0,1,1,0,1,1,1,0,1,1,0,0,1,1,1,0,1,1,1,1,0,1,0,1,1,1,1,1,1,1,1,1,1,0,0,1,0,1,1,1,0,1,0,1,1,1,0,1,1,0,0,1,0,1,0,1,0,0,0,0,1,0,0,1,1,1,0,0,1,1,1,1,1,0,0,1,1,1,1,1,0,0,0,0,1,0,1,1
I011_2013,0,0,0,0,0,1,0,1,0,0,1,0,0,0,1,0,0,1,1,0,0,1,0,0,0,0,1,1,1,1,1,1,0,0,0,1,0,0,1,0,1,0,0,1,0,1,0,0,1,0,1,0,0,1,0,0,0,0,0,0,1,0,0,0,1,1,0,0,0,1,0,0,1,0,0,0,0,0,0,0,0,0,1,0,0,0,1,1,1,0,0,1,1,1,1,1,1,0,0,0,1,0,0,0,0,0,1,0,1,1
I013_2013,0,0,1,0,0,1,0,0,0,0,1,0,0,0,1,0,0,1,1,0,1,1,1,0,1,0,0,1,1,0,0,1,0,0,0,0,0,0,1,0,1,1,1,1,1,1,1,1,1,1,0,1,1,0,0,0,1,0,1,0,1,0,1,0,1,1,1,0,1,1,0,0,1,0,1,0,0,0,0,0,0,0,1,0,0,1,1,1,1,0,1,1,0,1,1,1,1,1,0,0,1,1,1,0,0,0,1,0,1,1
I014_2013,0,0,0,0,0,0,0,0,0,1,0,0,0,0,0,0,0,1,0,1,1,1,0,0,0,0,1,0,0,1,1,1,1,1,0,0,0,1,1,0,0,0,1,1,0,1,1,1,1,1,1,1,1,1,0,0,0,0,0,0,1,1,0,1,0,1,0,1,1,0,1,1,1,0,0,0,0,1,0,1,0,0,0,0,1,0,0,0,1,1,0,0,1,1,1,1,0,1,0,1,1,0,1,1,0,0,0,0,1,0,1,1
I015_2013,0,0,1,0,0,1,0,1,1,1,1,0,0,1,1,1,1,0,0,1,1,1,1,0,1,0,0,1,1,1,1,1,0,0,1,1,0,0,1,1,1,0,1,1,1,1,0,1,0,1,1,1,1,1,0,0,0,1,0,1,1,0,1,0,1,0,0,1,1,1,0,0,1,0,1,0,1,0,0,0,0,1,0,0,1,1,0,1,0,1,1,1,1,1,1,0,0,0,0,1,1,1,0,0,0,1,1,1,1
I017_2013,1,1,1,1,1,0,0,1,1,1,1,0,0,0,0,1,0,1,1,1,1,0,0,1,1,0,0,1,1,0,1,1,1,1,0,1,1,0,1,1,1,0,1,1,1,1,1,1,1,1,1,1,1,1,1,0,0,0,1,0,0,1,0,0,0,1,0,0,1,1,1,0,1,0,0,1,0,1,0,0,0,1,1,0,0,1,1,1,1,0,1,1,1,1,1,1,1,1,1,0,0,0,0,0,1,0,0,1,0,1,1
I018_2013,1,0,0,0,0,0,0,0,1,1,1,0,1,1,0,1,1,1,1,1,1,0,0,0,1,0,1,1,1,0,1,1,0,1,1,1,1,0,0,0,1,1,1,0,1,0,1,1,1,1,1,0,0,1,0,0,0,0,0,0,1,0,1,0,1,1,0,1,1,1,0,0,0,1,1,0,1,0,0,0,1,1,0,0,1,0,1,1,0,1,1,1,1,1,1,1,0,0,1,0,0,0,1,1,0,0,0,1,1
I019_2013,1,0,1,1,1,1,1,1,1,1,0,0,1,1,1,1,1,1,0,1,1,1,1,1,1,0,1,1,1,0,1,1,0,1,0,1,0,0,0,0,1,0,1,1,1,1,1,1,1,1,0,1,1,0,1,0,1,1,1,1,0,1,0,1,0,1,1,1,1,0,1,1,1,1,1,1,1,1,1,0,0,0,1,1,0,0,1,1,1,0,0,1,1,1,1,1,0,1,0,0,1,1,1,0,0,0,1,1,1,1,1
I020_2013,0,0,1,0,0,1,0,1,0,0,1,0,0,1,0,1,0,1,1,1,1,1,1,1,1,0,1,1,1,1,1,1,0,1,1,1,1,0,1,1,1,1,1,1,1,1,0,0,1,1,1,1,1,1,1,1,0,0,1,1,1,1,1,0,1,0,1,1,0,1,1,1,0,1,1,0,1,0,1,0,0,0,0,1,0,0,0,1,1,0,0,1,1,1,1,1,0,1,0,1,0,1,1,1,0,0,0,0,1,1,1,1
I021_2013,0,0,1,1,0,1,0,0,1,0,0,0,0,0,1,1,0,1,1,1,1,1,1,1,0,0,1,1,1,1,1,1,0,0,0,0,0,0,1,0,1,1,1,1,1,1,1,1,0,1,1,0,1,0,0,1,1,1,0,0,1,0,1,0,1,1,1,1,1,1,0,0,0,0,1,0,1,0,0,1,1,1,0,0,1,1,1,1,0,1,1,1,1,0,0,0,1,0,1,1,1,0,0,0,0,0,0,1,1
I025_2013,0,0,1,0,0,1,1,1,1,1,1,0,0,1,1,1,0,1,1,1,1,1,1,1,1,0,1,1,1,1,1,1,0,0,1,1,0,0,0,0,1,1,1,1,1,1,1,1,1,1,0,1,1,1,0,0,0,1,1,1,1,0,0,0,1,0,1,0,1,1,0,1,1,0,1,1,1,0,0,0,0,1,0,0,1,1,1,1,0,1,1,1,0,0,1,0,0,0,0,1,1,1,1,0,1,0,1,1,1,1
I026_2013,0,0,1,0,1,1,1,1,1,1,1,1,1,0,1,1,1,1,1,1,1,1,1,1,1,0,1,1,1,1,1,1,1,0,1,1,1,1,0,1,1,1,1,1,1,1,0,1,1,1,1,1,1,1,1,0,1,0,1,1,0,1,0,1,0,1,0,1,0,1,1,1,1,0,1,0,0,1,1,1,0,0,0,1,1,0,0,1,1,1,1,0,1,1,1,1,1,1,1,0,1,0,1,1,1,1,0,0,0,1,1,1,1
I029_2013,0,0,1,0,1,1,0,0,1,1,0,0,1,1,0,1,1,0,0,1,1,1,1,1,1,0,0,1,1,0,1,1,1,0,1,1,1,0,1,1,1,1,1,1,1,1,0,1,1,1,1,1,1,1,1,1,0,0,0,1,1,0,1,0,1,0,1,1,0,1,1,1,0,0,0,0,1,0,1,0,0,0,1,1,0,0,1,1,1,0,0,1,1,1,0,0,0,0,1,1,1,1,1,1,1,0,1,0,1,1,1,1
I030_2013,0,0,1,1,1,1,0,1,1,1,1,0,1,0,0,1,1,1,1,1,1,1,1,0,1,1,0,1,1,1,1,1,1,1,1,1,1,1,1,0,1,1,1,1,0,1,1,1,0,1,0,1,0,0,0,1,0,1,1,0,1,1,1,0,0,0,1,1,1,0,1,1,1,1,1,1,1,1,1,1,0,0,0,0,1,0,0,1,1,1,0,0,1,1,1,1,1,1,1,0,0,0,0,0,0,0,0,0,0,0,0,0,0,1,0
I031_2013,0,0,1,0,1,1,1,0,1,1,1,0,0,1,0,1,0,1,1,1,1,1,1,1,1,0,0,1,1,0,1,0,0,1,1,1,1,0,1,1,1,1,1,1,1,1,1,0,1,1,1,1,0,0,1,0,0,0,0,0,0,1,0,1,0,1,1,0,1,0,1,1,1,1,0,1,1,1,0,0,0,0,0,0,0,1,1,1,1,0,1,1,1,0,0,1,0,1,0,0,1,1,0,0,0,0,1,0,1,0
I033_2013,0,0,1,0,1,1,0,1,1,1,1,0,0,1,0,1,0,1,1,1,1,1,0,1,1,0,1,1,1,1,1,1,0,0,1,1,1,0,0,0,1,1,0,1,1,1,1,1,1,1,1,1,1,1,1,0,0,1,1,1,1,1,0,1,0,1,1,1,1,1,1,0,1,0,0,1,0,1,0,0,0,0,1,0,0,0,1,1,1,0,1,1,1,1,1,1,0,1,1,0,1,1,1,0,1,0,1,1,1,0
I034_2013,1,0,1,0,1,1,0,0,1,1,1,0,0,0,0,1,1,1,0,1,0,1,1,1,1,0,1,1,1,0,0,0,0,0,0,1,1,0,0,1,1,1,1,1,1,1,1,1,1,1,0,1,1,1,1,0,1,1,1,0,1,1,0,1,0,1,1,1,0,0,1,1,0,1,0,1,0,1,0,0,0,1,1,1,0,1,1,1,0,0,1,1,1,1,0,1,0,0,1,0,1,1,0,0,1,1,1,1,1,1
I036_2013,0,0,1,0,1,1,1,1,1,1,1,0,0,1,1,1,1,1,1,1,1,1,0,0,1,0,0,1,1,1,1,0,0,1,1,1,1,0,1,1,1,1,1,1,1,1,1,1,1,1,1,1,1,1,1,1,1,0,0,1,0,1,0,1,0,1,0,1,1,1,0,1,1,0,0,1,0,1,0,1,0,0,0,1,1,0,0,1,1,1,0,0,1,1,1,1,1,1,1,0,1,0,1,1,1,0,0,0,0,1,0,1,0
I037_2013,0,0,1,1,0,0,0,1,1,0,1,0,1,0,0,0,1,0,0,1,0,1,1,0,1,0,0,0,1,1,1,0,1,0,0,1,1,0,1,1,1,1,0,1,1,0,0,1,0,1,0,1,1,1,0,0,1,1,1,1,1,0,0,0,1,0,0,0,1,1,0,0,0,0,1,0,1,0,0,0,1,1,0,0,1,1,1,0,0,1,1,1,0,0,1,0,1,0,1,1,1,0,0,0,0,1,0,1,0
I038_2013,0,0,1,0,0,1,0,1,1,1,0,0,1,1,0,0,0,1,1,1,1,1,1,1,0,0,1,1,1,1,1,1,0,1,1,1,0,0,1,1,1,1,1,1,1,1,1,0,1,1,1,1,1,1,1,1,0,0,0,0,1,1,1,0,0,0,1,1,0,0,1,1,1,1,1,1,1,1,1,0,0,0,0,1,0,0,1,1,1,0,0,1,1,0,1,1,1,1,1,1,0,1,1,1,0,0,0,0,1,1,1,1
I039_2013,0,0,1,0,0,1,0,1,1,0,1,0,0,1,0,1,1,1,1,1,1,1,1,1,1,0,1,1,1,1,1,1,0,1,1,1,1,0,0,1,1,1,1,1,1,0,0,1,1,1,1,1,0,1,0,1,0,1,1,1,1,0,1,0,1,1,0,1,1,1,0,1,1,0,1,1,1,0,0,0,1,1,0,0,1,1,1,1,0,1,1,1,1,0,1,0,1,0,1,1,1,0,0,1,0,1,1,1,1
I050_2013,0,0,1,0,0,1,0,1,1,0,1,0,0,1,1,1,1,1,1,1,1,0,0,1,1,0,0,1,1,0,0,1,0,1,1,1,1,0,0,0,1,0,0,1,0,1,0,1,1,1,1,1,0,0,0,0,1,0,0,1,1,0,1,0,1,1,1,0,1,1,0,1,0,0,1,0,1,0,0,0,0,1,0,0,1,1,1,0,0,1,1,0,1,1,1,0,1,1,0,1,1,1,0,1,1,1,1,1,1
I052_2013,1,0,1,0,1,1,0,1,1,1,1,0,0,1,1,1,1,1,1,1,1,1,1,1,1,0,1,1,1,0,1,1,1,1,1,1,1,1,0,1,1,1,1,1,1,1,0,1,0,1,1,1,1,1,1,1,1,0,0,1,0,1,1,1,0,1,0,1,1,1,1,1,1,1,1,1,0,1,0,1,0,0,0,0,1,0,0,1,1,0,1,0,1,1,1,1,1,0,0,1,0,0,1,1,0,0,0,0,1,1,1,1
I075_2013,0,0,1,1,1,0,1,1,1,0,1,1,1,0,0,1,1,1,1,1,1,1,1,1,1,0,0,1,1,0,0,1,0,0,1,1,1,0,1,0,1,0,1,1,1,1,1,0,1,1,1,1,1,1,1,0,0,0,0,1,1,1,0,0,1,1,0,1,1,1,1,0,0,1,0,1,1,1,0,0,0,1,0,0,0,0,1,1,0,0,1,0,1,0,0,1,1,1,0,0,1,1,1,0,0,0,1,1,1,1
I078_2013,0,0,1,0,1,0,1,0,0,1,1,0,0,0,1,1,0,0,0,1,1,1,0,1,1,0,0,1,1,0,0,0,0,0,0,1,0,0,0,1,1,1,1,1,1,1,0,0,0,0,1,1,1,0,0,0,0,1,0,1,1,1,1,1,0,1,1,1,1,0,1,0,0,0,1,1,0,0,0,0,0,0,0,0,0,0,0,0,1,0,0,0,1,1,0,0,1,1,1,1,0,1,0,1,0,0,0,1,1,1,0
I082_2013,0,0,0,0,0,0,0,1,0,1,1,0,1,0,0,1,0,1,1,1,1,1,0,1,1,0,1,1,1,1,1,0,0,0,1,1,0,0,0,1,1,0,1,1,1,1,1,1,1,1,1,1,1,0,1,0,0,1,1,1,0,1,0,1,0,1,1,1,1,1,1,0,1,1,1,1,1,1,0,0,0,1,1,0,0,1,1,1,0,0,1,1,1,1,1,0,0,1,1,1,1,1,1,1,0,0,0,1,1,1,1
J002_2014,0,0,0,0,1,0,0,1,1,0,1,0,1,0,1,1,0,1,1,1,1,1,0,1,1,0,0,1,1,1,1,1,0,1,1,0,0,0,0,1,1,0,0,1,0,1,0,1,1,0,0,0,0,0,0,0,1,0,0,0,1,0,0,0,1,1,1,1,1,1,0,1,0,0,1,0,0,0,0,0,0,0,0,0,0,1,1,1,1,0,1,1,1,0,0,1,0,0,0,0,1,0,0,0,0,0,1,0,1,1
J004_2014,1,0,1,1,1,1,0,1,1,1,0,0,0,1,0,1,0,1,1,1,1,0,0,0,0,0,0,1,1,0,1,0,0,1,1,1,0,0,1,0,1,1,0,1,1,0,0,1,1,0,1,1,0,1,0,0,1,0,1,1,1,0,1,0,1,1,1,1,1,1,1,1,1,0,1,0,1,0,0,0,0,1,0,0,1,1,1,1,0,1,1,1,0,0,1,0,1,0,1,1,1,0,0,0,0,0,1,0,1,1
J004_2017,1,1,1,0,1,1,0,1,1,1,0,0,0,1,1,1,0,1,1,1,1,1,1,1,1,1,1,1,1,1,1,1,1,0,1,1,1,0,0,1,1,1,1,1,1,0,0,1,1,1,0,1,0,0,1,0,0,1,1,0,0,1,0,1,0,1,1,1,1,1,1,0,1,1,0,1,1,0,0,0,0,0,1,1,0,1,1,1,1,0,1,1,1,1,1,1,1,0,1,1,0,1,1,1,0,1,1,1,1,1,1

J006_2014,1,0,1,1,1,1,0,1,1,0,1,1,1,1,1,1,0,1,0,1,1,1,1,1,1,0,1,1,1,1,1,1,0,1,1,1,1,0,0,1,1,1,1,1,1,1,1,1,1,1,1,1,0,1,0,0,1,0,1,0,1,0,1,0,1,1,1,1,1,1,1,1,1,0,1,1,1,0,0,0,1,0,0,0,1,1,1,1,0,1,1,1,0,0,0,0,1,0,1,1,1,1,0,0,0,1,0,1,0
J007_2014,1,0,1,1,1,1,1,1,1,1,0,0,0,1,0,1,1,1,1,1,1,1,1,1,1,0,1,1,1,0,1,1,0,1,1,1,1,0,1,1,1,1,1,1,1,1,1,1,0,1,1,1,1,1,0,0,0,0,1,0,1,0,1,0,1,1,1,1,1,1,1,0,1,1,1,1,0,1,0,0,0,1,1,0,0,1,1,1,1,0,1,1,1,0,0,1,0,1,1,1,1,1,0,0,1,0,1,1,1,0
J007_2017,1,0,1,0,0,1,1,1,1,1,1,0,0,1,0,1,0,1,1,1,1,1,0,1,1,0,1,1,1,0,1,1,0,1,0,1,1,0,0,0,1,1,1,1,1,1,1,1,1,0,1,0,1,1,0,1,1,0,1,1,1,0,1,0,1,0,0,0,0,1,0,1,1,1,1,0,1,0,0,0,1,1,0,0,1,1,1,1,0,1,1,1,0,0,0,0,1,0,0,1,1,0,0,0,1,0,0,1,1
J008_2014,0,1,1,1,0,1,0,0,1,0,1,0,0,0,0,1,0,1,1,1,0,0,1,1,0,0,0,1,0,1,1,0,1,1,0,0,0,1,0,1,1,1,1,1,1,1,1,1,1,1,0,0,1,1,1,0,1,0,1,0,0,0,1,1,1,1,1,1,1,0,1,0,1,1,1,0,0,0,0,1,0,0,0,1,1,1,0,1,1,1,1,0,0,0,0,1,0,0,0,1,1,1,0,1,1,1,1
J009_2014,1,0,1,0,1,1,0,0,1,1,0,0,1,1,0,1,1,1,0,1,1,0,1,1,1,0,1,1,1,1,1,1,1,1,1,1,1,0,1,1,1,1,1,1,1,1,1,1,0,1,1,1,0,1,0,1,0,0,1,0,1,0,1,0,1,1,1,0,0,1,0,0,1,1,1,0,1,0,0,0,1,1,0,0,1,1,1,1,0,1,1,1,1,1,1,0,1,0,1,1,1,0,0,0,0,1,1,1,1
J010_2014,0,0,1,1,1,1,0,1,1,0,1,0,1,1,0,1,1,1,1,1,1,0,0,1,1,0,1,1,1,0,1,1,1,1,1,1,1,0,0,1,1,0,1,1,1,1,1,1,1,1,1,1,1,1,0,0,1,0,1,0,1,0,1,0,1,1,0,1,0,1,1,1,1,0,1,1,1,0,0,0,1,1,0,0,1,1,1,1,0,1,1,1,1,1,1,0,1,0,1,1,1,0,0,0,0,1,0,1,1
J010_2017,1,0,1,1,1,1,0,1,1,1,1,0,1,1,1,1,1,1,0,1,1,1,1,1,1,0,1,1,1,1,1,1,1,1,1,0,0,0,1,1,1,1,0,1,1,1,1,1,0,1,1,1,1,0,1,1,1,1,1,1,0,1,0,1,0,0,1,1,1,0,1,1,1,1,1,1,0,0,0,1,1,0,0,0,1,1,1,0,1,1,1,1,1,0,0,0,1,0,1,1,0,0,0,0,1,1,1,1
J011_2014,1,1,1,0,1,1,0,0,1,1,1,0,1,0,0,1,1,1,1,1,1,1,0,0,0,0,1,1,1,1,1,1,0,1,1,1,1,0,0,0,1,0,1,1,1,1,0,1,1,1,1,1,1,1,0,1,1,0,0,1,1,0,1,0,0,1,1,1,1,1,0,1,1,0,1,1,1,0,0,0,1,1,0,0,0,1,1,0,0,1,1,1,1,0,1,0,1,1,0,1,1,0,0,0,0,1,0,1,1
J014_2014,1,0,1,0,0,1,0,0,1,1,0,0,1,1,0,0,0,1,0,1,1,0,1,1,1,0,1,1,1,1,0,0,0,0,0,0,1,1,0,1,1,0,1,0,0,1,0,1,1,0,0,0,1,1,0,1,0,0,1,1,1,0,1,0,0,0,1,0,0,1,0,0,1,1,1,1,1,0,1,0,0,0,1,0,0,1,1,0,0,0,1,1,0,0,1,0,1,0,0,1,1,0,0,1,1,1,1,1,1
J015_2014,1,0,1,1,0,0,0,1,1,0,0,1,1,1,0,0,0,1,0,1,1,1,1,1,1,0,1,1,0,0,0,0,0,1,1,1,1,0,1,1,1,1,1,1,1,1,1,1,1,1,1,0,1,0,0,1,0,0,0,1,0,1,0,1,1,0,0,1,1,1,1,1,0,1,0,1,0,0,0,0,1,0,0,1,1,1,0,0,1,1,1,1,1,1,0,0,0,1,1,1,0,0,0,0,1,0,1,0
J017_2014,1,0,1,1,1,1,0,1,1,1,0,0,1,1,1,1,1,1,1,1,1,0,0,1,1,0,1,1,1,1,1,1,0,1,1,1,1,0,0,0,1,1,0,1,0,1,1,1,0,0,1,1,1,1,0,0,1,1,1,1,1,0,1,0,1,1,1,0,0,1,1,0,1,0,1,0,1,0,0,0,1,1,0,0,1,1,1,0,0,1,1,1,0,0,0,0,1,1,1,1,1,1,0,0,0,1,0,1,1
J017_2017,1,0,1,1,1,1,0,1,1,1,0,0,1,0,0,1,1,1,0,1,1,1,1,0,1,0,1,1,1,1,1,1,0,1,1,1,0,0,0,1,1,1,1,0,1,1,0,1,1,1,1,1,1,1,1,0,1,1,0,0,1,1,0,1,0,1,1,0,1,1,1,0,0,1,1,1,0,1,0,0,0,1,1,0,0,1,1,1,0,0,1,1,1,1,1,1,1,1,0,1,1,1,1,1,1,0,0,0,1,1,1,1
J019_2014,0,0,1,0,1,1,0,0,1,0,0,0,0,0,0,1,1,1,1,0,1,1,1,0,0,0,1,1,0,1,1,0,1,0,0,0,0,1,0,1,0,1,0,1,0,0,1,0,1,0,1,1,1,0,1,1,1,1,1,1,0,0,0,1,1,1,1,1,1,1,0,0,0,1,1,1,0,0,0,1,0,0,0,0,1,0,1,0,1,1,1,1,0,0,1,0,0,0,0,0,0,0,1,0,1,0,1,1
J020_2014,1,0,1,0,1,1,1,1,1,1,1,0,1,0,1,1,1,1,0,1,1,1,1,1,1,0,1,1,1,1,1,1,0,1,1,1,1,0,0,0,1,1,1,1,1,1,1,1,1,1,1,1,0,1,0,1,1,1,1,1,1,0,1,0,1,1,1,1,1,1,1,1,1,1,1,0,1,0,0,0,0,1,0,0,1,1,1,1,0,1,1,1,1,1,0,0,1,0,1,1,1,1,0,1,0,1,1,1,1
J020_2017,1,0,1,1,1,1,1,1,1,0,1,0,0,1,1,1,1,1,0,1,1,1,1,1,1,0,1,1,1,1,1,1,0,1,1,1,0,0,0,1,1,1,1,0,0,1,1,1,1,1,1,1,1,1,1,0,1,1,1,0,1,1,0,1,0,1,1,1,0,1,1,0,1,1,1,1,0,1,0,0,0,0,1,0,0,1,1,1,1,0,1,1,1,0,0,1,1,1,0,0,1,1,0,0,1,0,1,0,1,1
J021_2014,0,1,1,1,1,0,1,1,1,1,1,0,1,1,1,1,0,1,1,1,1,1,0,1,1,0,1,1,1,1,1,1,0,1,1,1,0,0,0,0,0,0,1,1,1,1,1,1,1,0,0,1,1,1,0,1,1,0,1,1,1,0,1,0,1,1,1,0,0,1,1,1,1,0,1,1,1,0,0,0,1,1,0,0,1,1,1,1,0,1,1,1,1,0,0,0,1,0,1,1,1,0,0,1,0,0,0,1,1
J022_2014,1,1,1,1,1,1,0,0,1,0,0,0,1,1,0,1,1,1,1,1,1,1,0,1,1,0,1,1,1,1,1,1,0,1,1,1,0,0,0,1,1,0,1,1,1,1,1,1,1,1,1,1,1,1,0,1,1,1,1,1,1,0,1,0,1,1,1,0,0,1,1,1,1,0,1,1,1,0,0,0,1,1,0,0,0,1,1,1,0,1,1,1,1,0,1,0,1,0,1,1,1,0,0,1,0,0,1,1,1
J023_2014,0,0,1,0,1,1,0,1,1,1,0,0,1,0,1,1,1,1,1,1,1,1,0,1,1,1,0,1,1,1,1,1,1,0,1,1,1,1,0,0,1,1,1,1,1,1,1,0,0,1,1,1,1,0,1,0,0,1,0,1,1,1,0,1,0,1,1,0,1,1,1,1,1,1,1,0,1,0,1,0,0,0,0,1,0,0,1,1,1,0,0,1,1,1,1,1,1,0,1,1,1,1,1,1,1,0,1,0,1,0,1,1
J026_2014,1,0,1,0,1,1,0,1,0,0,0,0,0,1,1,1,1,1,1,1,1,1,0,1,0,1,0,1,1,1,1,1,1,0,1,1,1,1,0,0,0,1,1,0,1,0,1,1,1,1,0,1,1,0,1,0,0,1,1,1,0,1,0,1,0,1,0,0,1,1,1,1,0,1,1,1,0,0,0,0,1,0,0,1,1,1,0,1,1,1,1,1,0,0,0,1,1,1,1,1,1,0,0,1,0,1,0,1,1
J026_2017,1,0,1,0,1,1,0,1,1,1,0,0,0,1,1,1,1,1,0,1,1,0,1,0,1,0,0,1,1,1,1,1,0,1,1,1,0,0,0,0,1,0,1,1,1,1,1,1,1,1,1,1,1,1,0,1,1,1,1,0,1,0,1,0,1,1,1,1,1,1,1,0,1,1,0,1,1,1,0,0,0,0,1,0,0,1,1,1,0,0,1,1,1,0,0,0,0,1,1,1,1,1,1,1,0,1,0,1,1,1,1
J028_2014,0,1,1,0,1,1,1,1,1,0,0,0,0,0,0,1,0,1,1,1,1,1,0,0,1,1,0,1,1,1,1,1,1,0,1,1,1,1,0,1,1,1,1,0,1,0,0,0,0,0,0,0,1,1,1,0,0,0,0,1,1,1,0,1,0,1,1,1,0,0,1,1,1,1,0,1,0,1,0,0,0,1,1,1,0,1,1,1,1,0,1,1,1,1,1,1,0,1,1,1,1,1,1,1,0,0,0,1,1,1,1
J030_2014,1,1,1,0,1,1,0,1,1,0,1,0,0,0,0,1,0,1,1,1,1,1,0,1,1,0,1,1,1,1,1,1,1,1,1,1,1,0,0,1,1,1,1,1,0,1,1,1,1,0,1,1,1,1,0,1,1,1,0,0,1,0,1,0,1,1,1,0,1,1,1,1,1,1,1,0,0,0,0,0,1,1,0,0,1,1,1,1,0,0,1,1,1,1,1,1,0,1,1,0,1,1,1,0,1,0,1,0,1,1
J032_2014,1,0,1,1,1,1,0,1,1,1,0,0,1,1,0,1,0,0,0,1,1,1,1,1,1,0,0,0,1,1,1,1,1,1,1,1,1,0,0,1,1,1,1,1,1,1,1,0,1,1,1,1,1,1,0,1,0,1,1,1,0,1,0,1,1,1,0,1,0,1,0,1,0,0,0,0,1,0,0,1,1,1,1,0,1,1,1,1,1,1,0,1,1,1,1,1,1,1,0,0,1,0,1,1,1,1
J032_2017,1,0,1,1,1,1,0,1,1,0,1,0,0,0,0,1,1,1,1,1,1,0,1,1,1,0,0,1,1,1,1,1,1,1,0,1,1,0,0,1,1,1,0,0,0,0,0,1,1,1,1,1,1,1,0,0,0,0,1,1,1,0,1,0,1,1,0,0,1,1,1,1,1,1,1,0,0,1,0,1,1,1,0,1,1,1,0,0,1,1,1,0,1,0,0,0,0,0,1,1,1,0,0,0,0,1,0,1,1
J035_2014,1,0,1,1,1,1,0,1,1,1,1,0,0,0,0,1,0,1,0,0,0,0,1,1,1,0,1,1,1,1,1,1,0,1,1,0,0,0,1,0,1,1,1,1,1,0,1,1,1,1,1,1,0,1,0,1,0,0,0,0,1,0,0,0,1,1,1,1,1,1,1,1,0,0,1,0,1,0,0,0,0,1,0,0,1,0,1,1,0,1,1,0,0,1,1,1,1,1,1,0,0,0,1,1,0,1,1,0,1,1
J036_2014,0,0,1,0,1,1,0,1,1,1,1,0,1,1,1,1,1,1,1,1,1,1,0,1,1,0,1,1,1,1,1,1,0,1,1,0,1,0,0,1,1,1,1,1,1,1,1,1,1,1,1,1,1,1,0,1,0,1,1,1,1,0,1,0,1,1,1,1,1,1,1,1,0,1,0,1,0,1,0,0,0,0,1,0,0,1,1,0,1,0,1,1,1,1,1,1,1,0,1,0,1,1,0,1,0,0,0,1,0,1,1
J037_2014,1,0,1,0,1,1,1,0,1,1,1,0,0,1,1,1,1,1,0,1,1,1,1,1,1,0,1,1,1,1,1,1,0,1,1,1,1,0,1,1,1,1,1,1,0,0,1,1,1,1,1,1,0,1,0,1,0,1,0,0,1,0,1,1,1,1,1,1,1,1,1,1,1,0,1,1,1,0,0,0,0,1,0,0,0,1,1,1,0,1,1,1,0,1,1,0,1,1,1,1,0,1,1,0,0,1,0,1,1
J041_2014,1,1,1,0,0,1,1,1,1,1,1,1,1,1,1,1,1,1,1,1,0,0,0,1,1,0,1,1,1,1,1,1,1,1,1,1,1,0,1,1,1,1,1,1,1,1,1,1,1,1,1,1,1,1,1,1,0,1,1,1,1,0,0,1,1,1,0,1,1,1,0,1,1,1,0,1,0,0,1,0,1,0,0,0,1,0,1,0,1,1,1,0,1,1,0,1,1,0,1,1,1,1,1,1,0,1,1,1,1
J043_2014,1,0,1,1,1,1,1,1,1,1,0,0,0,0,1,1,1,1,1,0,1,1,0,1,0,1,0,1,1,1,1,1,1,0,1,1,1,1,0,1,0,1,1,1,1,1,1,1,1,1,1,1,1,1,1,1,1,1,0,1,1,1,1,1,1,0,1,0,1,1,1,1,0,1,1,1,1,1,1,1,1,1,1,0,0,0,1,1,1,0,1,1,1,0,0,1,1,1,1,1,1,1,0,1,0,1,1,1,0,0,1,0,1,0,1,1
J043_2017,1,0,1,1,1,1,1,1,1,1,0,0,0,1,1,1,1,1,1,1,1,1,1,1,1,0,1,1,1,1,1,1,0,1,0,1,0,0,1,0,1,0,1,1,0,0,1,1,1,1,1,1,1,1,1,0,1,1,1,1,0,1,0,1,0,1,1,1,1,1,1,1,1,1,1,0,1,1,1,0,0,0,1,1,0,0,1,1,1,0,0,1,1,1,0,0,0,0,1,1,1,1,1,1,1,0,1,0,1,1,1,1
J047_2014,1,0,1,1,1,1,1,1,1,1,1,1,1,1,1,1,1,1,1,1,1,1,1,1,1,1,0,1,1,1,0,1,1,0,1,1,1,1,0,0,1,1,1,1,1,1,1,1,1,1,1,1,1,1,1,1,1,1,1,1,0,1,1,1,0,1,0,1,1,1,1,1,1,0,0,1,0,1,0,1,0,0,0,0,1,1,0,1,1,1,0,0,1,1,1,0,1,0,0,1,1,1,1,1,1,0,0,0,0,1,1,1,1
J048_2014,0,0,1,1,1,1,1,1,1,1,1,0,0,1,0,1,1,1,1,1,1,1,1,0,1,0,1,1,1,0,1,1,0,1,1,1,1,0,1,1,1,1,1,1,1,1,1,1,1,1,1,1,1,1,1,1,1,1,0,1,1,1,0,1,0,1,1,0,1,1,1,0,1,1,0,1,1,1,0,0,0,0,1,1,0,1,1,1,0,0,1,1,1,1,1,1,1,0,1,0,1,1,1,0,0,0,0,1,0,1,1
J048_2017,0,0,1,0,0,1,1,1,1,1,1,0,0,1,1,1,0,1,0,1,1,1,1,0,1,0,1,1,1,0,1,1,1,0,1,1,0,0,0,1,1,1,1,0,0,1,1,1,1,1,1,1,1,0,1,0,0,1,0,1,0,1,0,1,0,1,0,0,0,0,1,0,1,1,1,1,1,1,0,0,0,1,1,0,0,1,1,1,0,0,1,1,1,0,1,0,0,1,0,1,1,1,0,0,0,0,1,1,1,1
J049_2014,0,1,1,0,1,1,1,1,1,1,1,1,1,0,1,1,1,0,0,1,1,1,1,1,1,0,0,1,1,0,0,0,0,1,1,1,1,0,0,1,1,0,1,1,0,0,0,0,0,1,1,1,1,1,0,1,1,0,0,1,0,0,1,0,1,0,1,1,0,1,1,0,1,0,1,0,1,0,0,0,1,1,0,0,1,1,1,0,0,1,1,1,0,0,1,1,1,1,0,1,0,1,0,1,0,1,0,1,1
J051_2014,0,0,1,1,1,1,1,1,1,1,1,1,0,1,0,1,1,1,1,1,1,1,1,1,1,1,0,0,1,1,0,1,1,1,1,1,1,1,1,0,0,1,1,1,1,1,1,1,1,1,1,0,1,1,1,1,1,0,1,1,1,1,1,1,1,0,1,0,1,1,1,1,1,1,1,0,1,1,0,1,0,1,0,0,0,0,1,0,0,0,0,1,0,0,1,1,1,0,0,0,0,1,0,1,1,1,1,1,0,1,0,1,1,1,1
J052_2014,0,0,1,1,0,0,1,1,1,0,1,0,0,0,0,0,0,1,0,1,1,1,1,0,0,0,1,1,1,0,0,1,1,1,0,0,0,0,1,0,1,1,0,1,1,1,1,1,1,1,1,1,1,1,1,1,0,1,1,0,1,1,1,0,1,0,1,1,1,0,1,1,0,0,0,0,1,1,1,0,0,0,0,1,0,0,1,1,1,0,0,1,1,1,0,1,1,0,1,0,1,1,1,1,0,0,0,0,1,1,1,1
J054_2014,0,0,1,1,1,1,1,1,1,1,1,1,1,1,1,1,1,1,1,1,1,1,1,1,1,1,0,1,1,1,1,1,1,1,1,1,1,1,1,1,0,1,1,1,1,1,1,1,1,1,1,1,1,1,0,1,1,1,1,0,1,1,1,1,1,1,1,0,1,0,1,1,1,1,1,1,0,1,1,0,1,0,1,0,0,0,0,1,1,0,1,1,1,1,0,1,1,1,1,1,1,1,1,1,1,1,1,1,1,0,0,0,1,0,1,1
J058_2014,1,0,1,0,1,0,1,1,0,1,1,0,1,1,1,0,0,1,0,1,1,1,1,1,1,0,1,1,1,0,0,0,1,1,1,1,1,0,1,1,1,1,1,1,1,1,0,1,1,0,1,1,1,1,1,0,1,1,1,1,1,1,0,0,1,1,1,1,0,1,1,1,1,0,0,1,0,1,0,0,0,1,1,0,0,1,1,0,1,0,1,1,1,1,0,1,0,1,0,1,1,1,1,1,0,1,0,1,0,1,1
J061_2014,1,0,1,0,0,1,0,0,1,0,1,0,1,0,0,1,0,0,0,1,1,0,0,0,0,0,0,1,1,0,0,1,0,1,1,1,0,0,1,0,1,0,1,1,1,1,1,1,1,1,1,0,0,1,0,0,0,0,1,0,1,0,1,0,1,1,0,0,0,1,0,1,1,0,1,0,1,0,0,0,1,1,0,0,1,1,1,0,0,1,1,1,1,1,1,0,1,0,1,1,1,1,0,0,0,1,0,1,1
J064_2014,1,0,1,1,1,1,1,1,1,1,1,0,1,0,0,0,0,0,1,1,1,1,1,1,1,1,0,1,0,1,1,1,1,1,1,1,1,1,0,0,1,1,1,1,1,1,1,1,1,1,1,1,1,0,1,1,1,1,1,1,1,1,1,1,1,1,1,0,1,1,1,1,1,1,1,1,1,1,1,1,1,0,1,0,0,1,1,1,0,0,1,1,1,1,0,1,1,1,1,1,1,1,1,0,1,1,1,1,1,1,1,0,0,1,1,1,1
J065_2014,0,0,1,0,0,1,0,1,0,0,1,0,1,1,1,1,0,0,0,1,1,0,0,0,0,0,1,1,1,1,0,0,0,1,1,1,1,0,0,1,1,1,1,1,1,1,1,1,0,1,1,1,1,0,1,0,0,1,0,1,1,1,0,0,1,1,1,0,1,1,1,1,1,1,1,0,1,0,1,0,0,0,1,1,0,0,1,1,1,1,0,1,1,1,0,0,1,0,1,0,1,1,1,1,0,0,0,1,1,1,1
J066_2014,0,0,1,1,1,1,1,1,1,1,1,1,1,0,1,1,1,1,1,1,1,1,1,1,1,1,0,1,1,1,0,1,1,0,1,1,1,1,0,1,1,1,1,1,1,1,1,1,1,1,1,1,1,1,0,1,0,0,1,1,1,0,1,1,1,0,1,1,1,1,1,1,0,0,1,0,1,0,1,0,0,0,1,1,1,0,0,1,1,1,0,1,1,1,0,0,0,1,1,1,0,1,1,0,0,1,0,1,1,1,1
J068_2014,1,0,1,0,0,1,0,0,1,1,0,0,1,0,0,1,0,1,0,0,1,1,1,1,1,1,0,1,1,1,0,0,0,0,0,1,1,0,1,0,1,1,1,1,1,0,1,0,0,1,1,1,0,1,1,0,1,1,0,1,0,1,0,1,0,1,0,1,1,1,1,0,0,1,1,1,1,1,0,0,1,0,0,0,0,1,0,1,1,0,1,1,1,0,0,1,0,1,0,0,1,1,0,0,0,0,0,0,1,1
J069_2014,1,0,1,0,1,1,0,1,1,0,0,0,1,1,1,1,1,1,0,1,1,1,0,1,1,0,1,0,1,0,1,1,0,1,1,1,1,0,1,1,1,1,1,1,1,1,1,1,1,1,1,1,1,1,0,1,0,0,1,0,1,0,1,0,1,1,0,1,1,1,1,1,1,1,0,1,1,1,0,0,0,0,1,0,0,0,1,1,1,0,1,1,1,1,1,1,1,0,1,0,1,1,1,1,0,0,0,1,1,1,1
J070_2014,1,0,1,1,1,1,1,1,1,1,0,1,1,1,0,1,1,1,1,1,1,1,0,1,0,1,0,1,1,1,0,0,1,0,1,1,1,1,0,0,1,1,1,1,1,1,1,1,1,1,1,1,1,1,1,1,0,1,1,1,1,1,1,0,1,0,1,1,0,1,1,1,0,0,1,0,1,0,1,0,0,0,0,1,1,0,1,1,1,0,0,1,1,1,0,0,0,0,1,1,1,1,1,1,1,0,0,0,1,1,1,1
J078_2014,1,0,1,1,1,1,0,0,1,1,0,1,0,1,0,1,1,1,1,1,1,1,1,1,1,0,1,1,1,0,1,1,0,1,1,1,1,0,1,1,1,1,1,1,1,1,1,1,1,1,1,1,0,1,0,1,1,1,1,1,1,0,1,0,1,1,1,0,1,1,0,1,1,0,1,0,1,0,0,0,0,1,1,0,1,1,1,0,0,1,1,1,1,1,1,1,0,1,0,1,1,1,1,0,0,0,1,1,1,1
J082_2014,1,0,1,0,1,1,0,1,1,1,0,0,0,1,1,1,0,1,1,1,1,1,1,1,1,0,1,1,1,0,1,1,1,1,1,1,1,1,0,0,1,1,1,1,1,1,0,0,1,0,1,1,1,1,1,0,1,1,1,1,0,1,0,1,0,1,1,0,1,1,1,1,1,1,0,1,0,1,0,0,0,0,1,0,0,1,1,1,0,0,1,1,1,1,1,1,1,1,1,1,1,1,1,0,0,1,0,1,1,1,1
J084_2014,1,0,1,0,0,1,0,1,1,1,1,0,1,0,1,1,0,1,1,1,1,1,0,1,1,0,1,1,1,1,1,1,0,0,1,1,1,0,0,0,1,1,1,1,0,0,0,0,0,0,1,1,1,1,0,1,1,1,0,1,1,0,1,0,1,1,1,1,0,1,1,1,1,1,1,1,0,1,0,0,0,1,1,0,0,1,1,0,1,0,1,1,1,1,1,0,0,1,1,0,1,1,1,1,0,0,1,1,1,1
J087_2014,0,0,1,1,1,1,0,1,1,1,0,0,0,1,1,1,1,1,1,1,1,1,1,1,1,1,0,1,1,1,1,1,0,0,1,1,1,0,0,1,0,1,1,1,1,1,0,0,1,0,1,1,1,1,1,0,1,1,1,1,1,1,0,1,0,1,1,1,1,1,1,1,1,1,0,1,0,1,0,0,0,0,1,0,0,1,1,1,0,0,1,1,1,1,0,0,1,0,0,1,1,1,0,0,0,1,1,1,1
J088_2014,1,0,1,0,0,1,0,1,1,1,0,0,1,1,0,1,1,1,1,1,1,1,0,1,1,0,1,1,1,0,0,0,0,0,1,1,1,0,0,0,1,1,0,1,0,1,0,0,1,0,1,1,1,1,1,0,0,0,0,1,1,0,1,0,1,0,0,0,0,0,0,0,1,1,0,1,1,1,1,1,0,1,1,0,0,0,0,0,1,1,1,0,1,0,0,1,0,0,1,1,1,0,0,0,0,0,1,0,1,1
J088_2017,1,0,1,0,1,1,1,1,1,1,1,1,0,1,0,1,1,1,1,1,1,1,1,1,0,1,1,0,1,1,1,1,1,1,1,1,1,0,1,0,0,0,0,0,1,1,1,1,0,0,0,1,1,0,1,1,0,1,0,1,1,0,0,1,1,0,0,0,0,1,1,1,1,1,1,0,0,1,0,1,0,0,0,0,0,0,0,1,0,0,0,0,1,0,1,0,1,1,1,1,1,1,1,1,0,0,0,1,1,0,0,0,0,0,1,0,1,1
J089_2014,0,0,1,1,1,1,1,0,1,1,1,1,1,1,1,0,1,1,1,1,1,1,1,1,1,1,1,0,0,1,1,0,1,1,0,1,1,1,1,0,0,0,1,1,1,1,1,0,1,0,0,1,1,1,1,1,0,1,1,0,1,1,1,0,1,0,1,1,1,1,1,1,0,0,1,0,1,1,1,0,0,0,0,1,0,0,1,1,1,1,0,1,1,1,0,1,1,1,1,1,0,1,1,0,0,0,0,1,1,1,0
J090_2014,1,0,1,1,1,1,1,1,1,1,0,1,1,1,1,1,1,1,1,1,1,1,1,1,1,1,0,0,0,1,0,1,1,1,1,1,1,1,1,0,1,1,1,1,1,1,1,1,1,1,1,1,1,1,1,0,1,0,0,0,0,1,1,1,1,1,0,1,1,0,1,1,1,0,1,1,0,1,0,1,0,0,0,0,0,1,1,0,0,1,1,0,0,1,1,1,0,0,1,1,1,0,1,1,0,1,0,0,0,1,1,1,1
J091_2014,0,0,1,0,1,1,1,0,1,1,1,0,1,1,0,1,1,0,1,1,0,0,0,1,1,0,1,1,1,0,1,0,0,1,0,1,1,0,0,1,1,1,1,1,0,1,0,1,0,1,1,1,0,1,0,0,1,1,1,1,1,1,1,0,1,1,0,1,1,1,0,0,1,0,1,0,1,0,0,0,0,1,0,0,0,0,1,0,0,1,1,1,0,0,0,0,1,0,1,1,0,0,0,0,0,0,1,1,1,0
J092_2014,1,0,1,1,0,1,1,1,1,1,1,0,0,1,0,1,1,1,0,1,1,1,1,1,1,0,1,0,1,0,1,1,0,1,1,1,1,0,1,1,1,1,1,1,1,1,1,1,1,1,1,1,1,1,1,1,1,0,1,1,0,1,1,1,0,1,0,1,1,0,1,1,1,1,1,1,1,0,1,0,1,0,0,0,0,1,1,0,1,0,1,1,0,1,1,1,1,1,1,1,1,1,1,0,1,1,1,1,0,0,0,1,1,1,1

J101_2014,1,0,1,0,0,1,0,0,1,0,0,0,0,0,1,1,1,1,1,1,0,0,0,0,0,1,0,0,0,1,0,0,1,1,1,0,0,0,1,1,1,0,0,0,0,0,0,1,1,0,1,1,0,0,0,0,0,1,1,0,0,0,1,1,1,1,0,1,0,0,0,0,1,0,1,0,0,1,0,1,0,0,1,0,1,1,0,1,1,1,1,1,1,1,1,0,0,1,1,0,0,0,0,1,1,1,1
J102_2014,1,0,1,1,1,1,1,1,1,1,0,1,0,0,0,1,1,0,0,1,0,1,0,1,1,0,1,1,0,1,0,1,1,1,1,1,1,0,1,1,1,1,1,1,0,1,1,1,1,0,1,1,1,1,0,1,1,1,1,1,1,0,1,0,1,1,1,1,1,1,0,1,1,0,1,0,1,0,0,1,0,1,0,0,1,1,0,0,0,1,1,1,1,1,1,0,1,0,1,1,1,0,0,1,0,1,1,1,0
J103_2014,1,0,1,0,0,1,1,1,1,0,0,1,0,0,0,1,1,1,1,1,1,0,0,1,1,0,0,1,1,1,0,1,0,1,1,1,1,0,1,1,1,1,1,1,0,1,0,1,0,1,1,1,1,0,0,1,1,0,1,1,0,0,0,1,1,1,1,1,1,0,1,0,0,1,0,1,0,0,1,0,1,1,0,0,1,1,1,0,1,1,1,0,0,0,0,1,1,0,1,1,0,1,1,0,1,0,1,1
J104_2014,1,0,1,1,1,1,1,1,1,1,1,1,1,1,0,1,1,1,1,1,0,0,0,0,1,0,1,1,1,0,1,0,0,1,1,1,1,0,0,1,1,1,1,1,1,1,1,1,1,1,1,1,1,1,1,1,0,1,1,1,0,1,0,1,1,1,1,1,1,0,0,1,0,1,1,1,0,0,0,0,1,0,0,1,1,1,0,0,1,1,1,0,1,1,1,1,1,1,1,1,0,0,1,0,1,1,1,0
J106_2014,1,0,1,0,1,1,0,0,0,0,1,0,0,1,1,1,1,1,0,1,1,0,1,0,1,0,1,1,1,1,1,1,1,1,0,0,0,0,1,1,0,1,0,1,1,0,1,0,1,1,1,1,1,1,0,0,1,1,1,0,1,0,1,0,1,0,1,0,0,1,1,1,1,0,1,0,1,0,0,1,1,0,0,0,1,1,1,1,0,1,1,1,1,1,1,0,1,0,1,1,1,0,0,0,1,0,1,1,1
J108_2014,1,0,1,1,1,1,0,1,0,0,1,0,1,1,0,1,1,1,0,0,1,0,0,0,1,0,1,1,1,1,1,1,0,0,1,1,0,1,1,1,1,1,1,0,1,1,0,0,1,0,1,1,1,1,0,1,1,1,0,1,1,0,1,0,0,1,0,1,0,1,1,0,0,1,1,1,1,0,1,1,0,0,1,0,0,1,1,0,0,1,1,1,0,0,0,0,1,1,1,1,1,0,0,0,1,1,1,1,1
J110_2014,1,0,1,1,1,1,1,1,1,1,1,1,1,0,1,1,1,1,1,1,1,1,0,0,1,0,0,1,1,0,0,1,0,0,1,1,0,0,1,0,1,1,1,1,0,0,0,0,1,0,1,1,1,1,0,1,1,1,1,1,0,1,0,1,1,1,1,1,1,1,1,0,0,0,1,0,1,0,0,1,0,1,1,0,1,1,1,0,0,1,1,1,0,0,0,1,1,0,0,1,1,0,1,0,0,1,0,1,1
J111_2014,1,0,1,1,0,1,0,1,1,0,1,1,1,0,0,1,1,1,0,1,1,1,1,0,0,0,1,0,0,1,0,1,0,1,1,1,0,0,0,1,1,1,1,1,1,1,0,1,1,0,1,0,1,1,0,0,0,0,0,1,1,0,1,0,1,1,0,1,1,1,0,0,1,0,1,1,0,1,0,0,0,0,0,0,0,0,1,0,0,1,1,1,0,1,1,0,1,0,0,1,1,0,0,1,0,1,1,1,1
J111_2017,1,0,1,0,0,1,0,1,1,1,0,0,1,1,0,0,1,0,1,1,1,1,1,1,1,0,0,0,1,1,1,1,0,1,0,0,1,0,1,0,1,1,1,1,0,1,1,1,0,1,1,1,1,1,0,0,0,0,1,0,1,0,1,0,1,1,1,1,1,1,1,1,1,1,1,1,0,1,0,0,0,0,1,0,0,1,1,0,0,0,1,1,1,0,1,1,1,1,0,1,1,1,0,0,0,0,0,0,1,1
J113_2014,1,0,1,1,1,1,0,1,1,1,0,1,1,0,0,0,1,0,1,1,1,1,1,1,1,0,0,1,1,1,1,1,0,1,1,1,1,0,0,1,1,1,0,1,1,0,1,1,1,0,1,1,1,1,0,1,1,1,0,1,1,0,0,0,1,1,0,1,1,1,0,1,1,0,1,0,1,0,0,1,0,0,0,0,0,0,1,0,0,1,1,1,0,0,0,1,1,0,1,1,1,0,0,0,0,1,1,1,1
J116_2014,0,0,0,0,1,0,0,1,1,0,0,0,0,1,0,1,0,1,1,0,1,1,1,0,0,0,1,1,1,1,0,0,0,1,1,1,0,0,1,1,1,1,1,1,1,1,1,1,1,1,1,1,1,1,0,0,1,0,0,0,1,0,1,0,1,0,0,0,0,1,0,0,1,0,1,0,1,0,0,1,0,1,0,0,1,0,1,0,0,1,1,1,0,0,0,0,0,0,1,1,1,0,0,0,0,1,0,1,0
J117_2014,0,0,0,0,1,1,0,1,1,0,1,0,0,1,0,1,0,1,1,0,1,1,1,0,0,0,1,1,1,1,0,0,0,1,0,1,0,0,1,1,1,0,0,0,1,1,1,1,1,1,1,1,1,0,0,0,1,0,0,0,1,0,1,0,1,0,0,0,0,1,1,1,1,0,1,1,1,0,0,1,0,1,0,0,1,0,1,0,0,1,1,1,0,0,0,0,1,0,1,1,0,1,1,0,0,1,0,1,1
J118_2014,1,0,1,0,0,1,0,1,1,1,0,0,1,1,0,1,0,1,0,1,1,1,0,1,1,0,0,1,1,1,1,1,0,0,1,1,1,0,0,1,1,1,1,1,1,1,1,1,1,1,1,1,0,1,0,1,1,1,1,1,1,1,1,0,1,1,1,1,1,1,0,1,1,0,1,0,1,0,0,1,1,1,0,0,1,1,1,0,0,1,1,1,1,1,1,0,1,0,0,1,1,0,1,0,0,1,1,1,1
J120_2014,0,0,1,0,0,1,0,0,1,0,0,1,0,0,0,1,0,1,0,1,1,0,0,1,1,0,0,1,1,1,1,1,0,1,1,1,1,0,1,0,1,1,1,1,1,0,0,0,1,0,0,1,1,0,1,1,1,1,1,1,0,1,0,1,1,1,0,0,1,1,1,1,0,1,0,1,0,0,1,0,1,0,0,1,1,1,1,0,1,1,1,0,0,1,0,1,0,1,1,1,1,0,1,0,1,1,1,0
J121_2014,1,0,1,0,0,0,0,1,1,0,0,1,0,1,0,1,0,1,0,1,1,0,0,1,1,0,0,1,1,1,0,1,0,0,1,1,0,0,1,0,1,1,1,1,1,0,0,1,1,1,1,1,1,0,0,0,0,1,1,1,0,1,0,1,1,1,1,1,1,0,1,1,0,1,0,1,0,0,1,0,1,0,0,1,1,0,1,0,1,1,1,1,1,1,0,1,1,1,1,1,1,1,1,0,1,0,1,1
J122_2014,0,0,1,0,0,1,0,0,1,0,1,0,1,1,0,1,0,1,1,1,1,1,1,1,1,0,1,1,1,1,1,1,0,1,1,1,0,0,0,0,1,1,1,1,1,1,0,0,0,1,1,1,1,1,0,0,1,1,1,0,1,0,1,0,1,1,0,1,1,1,1,1,1,0,1,0,1,0,0,0,0,0,0,1,1,1,0,0,1,1,1,1,1,1,0,1,0,0,1,1,0,0,1,0,1,0,1,1
J124_2014,1,0,1,0,0,0,0,1,1,0,1,0,1,0,0,1,0,0,0,1,1,1,0,1,1,0,0,1,1,1,0,1,0,0,0,1,0,0,0,1,1,1,1,1,1,1,1,1,1,1,1,1,1,1,0,0,0,0,1,1,1,0,1,0,1,1,1,1,1,1,0,1,0,0,1,0,1,0,0,0,0,1,0,0,1,1,1,1,0,1,1,1,1,1,1,0,1,1,0,1,1,1,0,1,0,1,0,1,1
J129_2014,1,0,1,1,1,1,0,1,1,0,1,1,1,0,0,1,1,1,1,1,0,1,0,1,1,0,0,1,1,0,1,1,0,1,1,1,1,0,1,0,1,0,1,1,1,0,0,1,1,0,1,1,1,1,0,1,1,1,1,1,0,1,0,1,1,1,0,1,1,1,0,1,0,1,0,1,0,0,1,0,1,0,0,1,0,1,0,0,1,1,1,0,1,1,1,1,0,0,1,1,1,0,0,0,1,0,1,1
J132_2014,1,0,1,0,1,1,1,1,1,1,1,0,1,0,0,1,1,0,1,1,0,0,0,1,1,0,1,1,1,1,1,1,0,1,1,1,1,0,1,1,1,0,1,0,1,0,0,0,1,1,1,0,1,1,0,0,1,1,1,1,1,0,1,0,1,1,1,1,1,1,0,1,1,0,1,0,1,0,0,1,0,1,0,0,0,1,1,1,0,1,1,1,1,0,0,0,0,0,0,0,0,0,0,0,0,0,1,0,0
J133_2014,1,0,1,1,1,1,1,1,1,0,0,0,1,1,0,1,1,1,0,1,1,0,1,1,1,0,0,1,1,0,1,1,0,1,1,1,1,0,1,1,1,1,0,1,0,0,1,1,1,1,0,1,1,0,1,1,1,1,1,1,0,1,0,1,0,0,1,1,1,1,1,0,0,1,0,1,0,0,1,0,1,0,0,0,1,1,0,0,1,1,1,1,1,1,0,1,1,1,1,1,1,0,0,0,1,1,1,1
J134_2016,1,0,1,0,1,1,0,0,1,1,1,0,0,1,0,1,1,1,1,1,1,1,1,1,1,1,1,1,1,1,1,1,0,1,1,1,1,0,0,1,1,1,1,1,1,1,1,1,1,1,1,1,1,0,1,0,1,1,1,0,1,0,1,1,1,1,1,1,1,1,0,0,1,0,1,0,0,0,0,1,0,0,1,1,1,0,0,1,1,1,1,1,1,0,1,0,1,1,1,1,1,0,0,1,1,1,1
J135_2014,0,0,1,0,1,1,1,1,1,1,1,0,0,1,0,1,0,0,1,1,1,0,0,1,1,0,0,1,1,1,1,1,0,1,1,1,1,0,0,1,1,1,1,0,0,0,0,0,0,1,0,0,1,1,0,1,0,0,1,1,1,0,1,0,1,1,1,1,0,1,0,0,1,0,1,0,1,0,0,1,0,1,0,0,0,1,1,1,0,1,1,1,1,1,1,0,1,0,1,1,0,0,0,0,0,1,0,1,0
J144_2014,0,0,1,1,1,1,0,1,1,1,1,0,0,0,0,1,1,1,1,1,1,0,0,1,1,0,0,1,1,0,1,0,1,1,1,1,0,0,0,1,1,1,1,1,0,1,0,1,1,1,1,1,0,1,0,1,0,1,1,1,1,1,0,1,1,1,0,1,1,0,1,0,0,1,0,1,0,0,0,0,1,0,0,0,1,0,0,0,1,1,1,1,1,1,1,1,1,1,1,1,1,0,0,0,1,1,1,1
J147_2014,1,0,1,1,1,1,0,1,1,1,1,1,1,1,1,1,1,1,1,1,1,1,1,1,0,1,1,1,1,1,1,1,1,1,1,0,0,0,1,1,1,1,1,1,1,0,1,1,1,1,1,0,1,1,1,1,1,1,0,1,0,1,1,0,1,1,1,0,0,0,1,0,1,0,0,0,1,1,1,0,0,0,1,0,0,1,1,1,1,1,1,1,1,0,1,1,1,1,0,0,0,1,1,1,1
K025_2015,1,1,1,1,1,1,0,1,1,1,1,0,1,0,0,1,0,1,1,1,1,1,0,0,1,0,1,1,1,1,1,1,0,1,1,1,1,0,1,0,1,0,1,1,0,1,1,1,0,1,1,1,1,1,0,1,0,0,1,1,1,0,1,0,1,1,1,1,1,1,0,0,0,1,0,1,0,0,0,1,1,0,0,0,1,1,0,0,1,1,1,0,0,1,0,1,1,0,1,1,1,0,0,0,1,0,1,0
K027_2015,0,1,1,0,0,1,1,1,1,0,0,0,0,1,1,1,0,1,1,1,1,0,0,0,1,0,0,1,1,1,1,1,0,0,1,1,1,0,0,0,1,0,1,1,1,0,1,0,0,1,0,0,1,1,0,1,1,1,1,0,1,0,1,0,1,0,1,1,1,1,1,1,1,0,1,1,1,0,0,0,1,1,0,0,1,1,1,0,0,1,1,1,1,1,1,0,1,0,1,1,1,0,0,0,0,1,1,1,1
K029_2015,1,1,1,1,0,0,1,1,1,1,1,0,1,1,0,0,1,0,0,1,1,1,0,1,1,0,0,1,1,1,0,1,0,1,1,1,0,0,0,0,1,1,1,1,1,1,0,0,0,1,1,1,0,1,0,1,0,1,1,1,1,0,1,0,1,1,0,1,0,1,1,1,1,0,1,1,0,0,0,0,0,1,0,0,1,1,1,0,0,1,1,1,1,1,0,0,1,0,0,1,1,0,0,0,0,1,1,1,1
K034_2015,0,0,0,0,0,0,0,1,1,0,1,0,1,1,0,1,0,1,1,1,1,0,0,1,0,0,0,1,1,1,0,0,0,1,1,1,1,0,0,0,1,0,1,1,0,1,0,1,1,1,1,0,0,1,0,0,0,1,0,0,1,0,0,0,1,1,0,0,0,1,0,0,1,1,1,1,1,0,0,0,1,1,1,0,0,1,1,1,0,1,1,0,1,1,1,1,0,0,0,0,0,0,0,0,0,0,0,0,1,1
K035_2015,0,0,1,1,1,1,0,1,1,1,0,0,0,1,1,1,0,1,1,1,1,1,1,1,1,0,1,1,1,1,1,1,1,1,1,1,1,0,0,1,1,1,1,1,1,1,1,1,1,1,1,1,0,1,0,1,1,1,1,0,1,0,1,0,1,1,1,1,1,1,0,1,1,0,1,0,1,0,0,0,0,1,0,0,1,1,1,0,0,1,1,1,0,0,1,0,1,0,1,1,1,0,0,1,0,1,0,1,1
K037_2015,0,1,1,0,0,1,0,1,1,1,1,0,1,0,1,1,0,1,1,1,1,1,0,1,1,0,0,1,1,1,1,1,1,1,1,1,0,0,0,1,1,1,1,1,1,1,1,1,1,1,0,1,1,1,0,1,1,0,1,1,1,0,0,1,1,1,0,1,0,1,0,1,0,0,1,1,1,0,0,0,0,1,0,0,0,1,0,1,0,1,1,1,0,0,1,1,1,0,0,1,0,1,0,0,0,1,1,1,1
K040_2015,0,0,1,1,0,1,1,1,1,0,1,0,1,0,0,1,0,1,0,1,1,1,0,1,0,0,0,1,1,0,0,1,0,1,1,1,1,0,0,1,1,1,1,1,1,1,1,1,1,0,1,1,1,0,0,1,0,1,1,1,0,1,0,1,1,1,1,0,1,1,1,1,0,1,0,1,0,0,0,1,1,0,0,1,1,1,0,0,1,1,1,1,1,1,0,1,1,1,1,1,1,0,1,0,1,1,1,0
K046_2015,0,0,1,0,1,1,1,1,0,1,1,1,1,1,0,1,0,1,1,1,1,0,0,1,1,0,0,1,1,0,1,1,0,0,1,1,1,0,0,0,1,1,1,1,1,0,1,0,0,1,1,1,1,1,0,0,0,1,1,1,1,1,0,1,1,0,0,1,1,1,1,1,1,0,1,1,1,0,0,0,1,1,0,0,0,0,1,1,0,1,1,1,0,0,1,1,1,0,0,1,1,1,0,1,1,1,1,1,1
K050_2015,0,0,0,0,0,1,0,1,0,1,0,0,0,0,1,1,0,1,0,1,1,1,1,0,1,0,1,0,1,1,0,0,0,1,0,1,0,0,0,0,0,0,1,1,1,1,1,1,1,1,1,0,1,1,0,0,0,1,1,0,1,0,1,0,1,0,0,1,1,1,0,0,0,1,1,1,1,0,0,0,0,1,0,0,1,1,1,1,0,1,1,1,1,1,0,0,0,1,1,1,1,0,0,1,1,1,0
K051_2015,1,0,1,0,1,1,0,1,0,1,0,0,0,0,0,1,0,1,1,1,1,1,1,0,1,0,1,1,1,1,0,0,1,1,0,1,0,0,0,1,1,1,1,1,1,1,1,1,1,1,1,1,1,0,0,0,0,0,1,0,1,0,1,0,1,0,0,1,1,1,1,0,1,0,1,0,1,0,0,0,0,0,0,0,1,1,1,0,0,1,1,1,0,1,1,0,1,0,1,1,1,0,1,0,0,1,0,1,1
K053_2015,0,0,1,0,1,0,0,0,1,0,1,0,1,1,0,1,0,0,0,1,1,1,0,0,1,0,0,1,1,1,1,1,0,1,1,1,1,0,1,1,1,1,1,1,1,0,1,1,1,1,1,1,0,1,0,1,1,0,1,1,0,1,0,1,1,0,1,0,1,0,1,1,0,1,0,1,0,0,0,0,1,0,0,1,1,1,0,0,1,1,1,1,0,1,1,1,0,1,1,1,0,0,0,0,1,1,1,1
K058_2015,1,0,1,0,1,1,0,1,1,1,0,0,0,1,1,1,0,1,1,1,1,1,1,0,1,0,1,1,1,1,0,0,1,1,1,1,0,0,0,0,0,0,1,1,1,1,1,1,0,1,0,1,1,0,0,0,1,1,0,1,1,1,0,1,0,0,1,1,1,1,0,0,0,1,1,1,0,0,1,0,0,1,0,0,1,0,1,0,1,1,1,0,1,1,1,0,0,1,1,0,0,1,0,0,1,0,1,1
K059_2015,1,0,0,0,1,1,0,1,0,1,0,0,0,0,0,0,1,1,0,1,1,1,0,1,0,1,1,1,1,0,0,1,1,1,0,0,0,0,1,1,1,1,1,1,1,1,1,1,1,1,1,1,1,0,1,0,1,0,1,0,1,0,0,1,1,1,0,0,0,1,1,1,0,0,1,0,1,0,0,0,1,1,0,0,1,1,1,1,1,1,0,0,0,1,1,1,1,1,0,1,1,1,1
K060_2015,1,1,1,0,0,1,0,1,0,0,1,1,0,0,0,0,0,1,1,1,0,0,0,1,1,0,0,0,1,0,1,1,0,1,1,1,0,0,0,1,1,1,1,1,0,1,0,0,0,1,1,1,0,1,0,0,0,0,1,1,1,0,0,1,1,1,0,1,1,1,1,0,1,1,1,0,1,0,0,0,1,1,0,0,0,1,1,0,0,1,1,1,1,1,1,0,1,1,1,1,1,1,0,0,0,1,1,1,1
K061_2015,0,1,1,0,1,1,0,1,1,0,1,1,1,1,1,1,1,0,0,1,0,0,1,0,1,0,0,1,1,1,1,1,0,1,1,1,0,0,1,0,1,1,1,1,1,1,1,1,1,1,1,1,1,1,0,1,0,1,1,1,1,0,0,1,1,0,0,0,1,1,1,1,1,0,1,0,1,0,0,0,1,1,0,0,0,1,1,0,0,1,1,0,0,0,1,1,1,0,1,1,1,1,0,0,1,1,0,1,1
K062_2015,1,0,1,0,1,1,1,1,1,1,1,0,0,1,1,1,0,1,1,1,1,1,1,0,1,0,0,0,1,0,0,1,0,1,1,0,1,0,1,1,1,1,1,1,0,1,0,0,0,1,1,1,0,1,0,1,1,0,0,1,1,0,1,0,1,1,0,1,0,1,1,1,1,1,1,0,1,0,0,0,0,1,0,0,1,1,1,0,0,1,1,1,1,1,1,0,1,0,1,1,1,1,1,0,0,1,1,1,0
K063_2015,0,0,0,0,0,0,0,1,0,0,1,0,0,0,1,0,0,1,0,1,1,1,1,0,1,0,1,0,1,1,0,0,1,1,1,0,1,0,0,0,0,0,1,1,1,1,1,1,1,1,1,0,0,1,0,0,0,0,1,0,1,1,1,0,1,0,0,1,1,1,0,0,0,0,1,1,1,0,0,1,1,0,0,0,0,1,0,1,0,1,0,1,0,1,1,1,1,0,1,1,1,1,1,0,0,1,1,1,1
K064_2015,1,0,1,1,1,1,0,1,0,1,1,0,1,1,1,1,0,1,1,1,1,1,1,1,1,0,0,1,1,0,1,1,0,1,1,1,1,0,0,0,1,1,1,1,1,1,0,0,1,1,1,0,1,0,1,0,0,1,1,1,0,1,0,1,1,1,1,1,0,1,0,0,0,1,1,1,0,0,0,1,0,0,0,1,1,1,0,0,1,1,1,0,0,1,1,1,1,1,1,1,1,1,0,0,0,1,1,1,0
L002_2016,1,0,1,1,1,1,0,1,1,0,1,0,1,1,0,1,1,1,0,1,1,1,1,1,1,0,1,1,1,1,1,1,1,1,1,1,1,0,0,1,1,1,1,1,1,1,1,1,1,1,1,1,0,1,0,1,0,0,1,1,1,0,1,0,1,1,1,1,1,1,0,1,1,0,1,0,1,0,0,0,1,1,0,0,1,1,1,0,0,1,1,0,1,1,0,0,1,0,1,1,1,1,1,0,0,1,1,1,1
L003_2016,0,0,1,1,0,1,0,1,1,0,1,0,1,0,0,1,0,0,0,1,0,1,0,1,1,0,1,1,1,0,1,1,1,1,1,1,0,0,1,1,1,1,1,1,1,1,1,1,1,1,1,1,1,0,0,0,0,1,1,0,1,0,1,1,0,1,1,1,0,1,1,0,1,0,1,0,0,0,1,1,0,0,1,1,1,0,0,1,1,1,0,0,0,0,1,1,1,1,1,1,1,1,0,0,1,0,1,1
L003_2017,1,1,1,0,1,1,0,1,1,0,1,0,0,1,0,1,0,1,0,1,1,0,1,0,1,0,0,1,1,0,0,0,1,1,0,0,1,0,0,1,1,1,1,1,0,1,1,1,0,1,1,0,1,1,0,0,1,0,1,1,1,0,1,0,1,0,0,0,1,1,1,1,1,1,1,1,0,0,0,0,1,1,0,0,1,1,1,0,0,1,1,1,0,0,0,0,1,0,1,1,1,1,0,0,0,0,1,1,1
L004_2016,1,1,1,1,0,1,0,1,1,1,1,0,1,1,0,1,0,0,1,1,1,1,0,1,1,0,1,1,1,1,1,1,1,1,1,1,1,1,0,0,1,1,1,1,1,1,1,1,1,1,1,1,0,1,0,1,1,1,1,1,0,1,1,1,1,1,1,1,1,0,0,1,0,1,0,1,0,0,0,1,1,0,0,0,1,1,1,0,1,1,1,1,1,1,0,1,1,1,1,1,1,0,0,0,1,1,1,1
L004_2017,1,0,1,0,1,1,0,1,1,0,1,0,1,1,0,0,0,1,0,1,1,0,0,1,1,0,0,1,1,0,0,0,1,1,0,0,0,0,1,1,1,0,1,1,1,0,1,0,1,0,0,0,1,0,1,0,0,1,1,1,0,0,0,1,0,0,0,0,1,0,0,1,0,1,0,1,0,0,0,0,1,0,0,0,1,1,1,0,1,1,1,0,0,1,0,1,0,1,1,1,1,0,1,1,0,1,1,1
L005_2016,1,0,0,0,0,1,0,0,0,0,0,0,0,0,0,1,0,0,0,1,1,0,0,1,1,0,0,1,1,0,1,1,0,1,1,1,1,0,0,1,1,1,1,1,1,1,1,1,1,1,0,0,1,0,1,0,0,0,0,1,0,0,0,1,1,0,1,0,1,0,1,1,0,1,0,1,0,0,0,1,1,0,0,0,1,1,1,0,1,1,1,1,1,0,0,1,0,1,1,1,1,0,0,0,1,1,1,0
L005_2017,1,0,1,1,1,1,0,1,1,0,1,0,0,0,0,1,1,1,0,1,1,1,0,1,1,0,1,1,1,0,1,1,0,0,0,1,0,0,0,1,1,1,1,1,1,1,1,1,1,0,1,1,0,1,0,0,1,1,1,1,1,1,0,1,0,1,1,1,0,1,1,0,1,1,1,1,1,1,0,0,0,1,1,0,0,1,1,1,0,1,1,1,0,0,0,1,0,0,1,1,1,0,1,1,1,1,1,1
L006_2016,0,0,1,1,0,1,1,1,1,0,0,0,1,0,0,1,1,0,0,1,1,0,0,1,1,0,0,1,1,0,1,0,0,1,1,1,1,0,1,1,1,0,1,1,0,1,1,1,1,1,1,1,0,1,0,1,1,0,0,1,1,0,0,0,1,1,0,1,1,1,0,1,1,0,1,0,1,0,0,0,1,1,0,0,0,1,1,0,0,1,1,1,1,1,0,0,1,1,1,1,1,1,0,0,1,1,0,1,1
L009_2016,0,0,1,0,1,1,1,1,1,1,0,0,1,1,1,1,1,0,0,1,0,1,1,1,1,0,1,1,1,0,1,1,0,1,1,1,0,0,0,1,1,1,1,1,0,1,0,1,1,1,1,1,1,0,0,1,1,1,1,1,0,0,0,1,1,1,0,1,1,1,0,1,0,1,0,1,0,0,0,0,1,0,0,1,1,1,0,0,1,1,0,1,1,1,1,0,0,0,1,0,0,0,0,0,0,1,1,0
L009_2017,0,0,1,1,0,1,0,1,1,1,0,0,1,1,0,1,0,0,0,1,0,0,1,0,1,0,1,1,1,0,0,0,0,1,0,1,0,0,0,0,1,1,1,1,1,0,1,0,1,1,1,0,1,1,0,1,1,1,0,1,0,1,0,1,0,1,0,0,1,1,0,1,1,1,0,1,0,0,0,1,1,0,0,1,1,1,1,0,1,1,1,0,0,0,0,1,0,1,1,0,0,0,0,0,0,0,1,1
L010_2016,0,0,1,0,1,1,0,1,1,0,1,0,1,1,0,1,1,1,1,1,1,1,1,1,1,0,0,1,1,0,1,1,1,1,1,1,1,0,0,1,1,0,1,1,1,1,1,1,0,1,1,1,1,1,0,1,1,0,1,1,0,1,0,1,1,1,1,1,1,1,1,1,0,1,1,1,0,0,0,1,1,0,0,0,1,1,0,0,1,1,1,1,1,1,1,1,1,1,1,1,0,0,0,0,1,1,1
L011_2016,1,0,1,0,0,1,0,0,1,1,0,0,0,1,1,1,1,1,0,1,1,1,1,1,1,0,1,1,1,0,1,1,0,1,1,1,0,0,0,1,1,1,1,1,0,1,1,1,1,1,1,1,1,0,1,0,1,1,1,1,1,0,1,1,1,1,1,1,0,1,1,0,1,0,1,0,0,0,1,1,0,0,1,1,0,1,0,1,1,1,1,1,1,1,1,0,1,1,0,1,0,1,0,1,1,1,1
L011_2017,1,1,1,0,0,1,1,1,1,1,0,0,1,0,0,0,1,1,1,1,1,0,1,1,1,0,1,1,1,1,1,1,0,1,0,1,0,0,1,0,1,1,1,1,1,0,1,1,1,1,0,1,1,0,0,1,1,1,0,1,0,1,0,1,0,0,1,1,1,1,1,1,1,1,0,1,0,0,0,1,1,0,0,1,0,1,1,0,1,1,1,0,0,1,0,1,1,1,1,1,0,0,0,0,0,1,1,1

L013_2016,1,0,1,1,0,1,0,1,1,1,0,0,1,0,1,1,1,1,1,1,1,0,1,1,0,1,1,1,1,0,1,0,1,1,1,0,0,0,1,1,1,1,1,1,1,1,0,1,1,1,1,1,0,1,1,1,0,1,1,0,1,1,1,1,1,1,1,1,1,1,1,1,1,0,1,0,0,0,1,0,0,0,0,1,1,1,0,1,1,1,1,1,0,0,1,0,1,1,0,1,0,0,0,1,1,1,0
L014_2016,0,0,1,1,1,0,1,1,1,1,1,0,1,1,0,1,1,1,1,1,1,1,1,1,1,0,0,1,1,0,1,1,0,1,1,1,0,0,0,0,1,1,1,1,1,1,1,1,1,1,0,1,1,0,1,1,0,1,1,0,1,1,1,0,1,0,1,1,1,1,1,1,1,1,0,0,1,1,1,0,0,1,1,0,0,0,1,1,1,0,0,1,1,1,1,1,1,0,1,1,1,1,1,0,0,0,0,1,1,1,0
L014_2017,0,0,1,0,1,1,1,1,1,1,1,0,0,1,1,1,1,1,0,1,1,1,1,1,1,0,1,1,1,0,1,1,0,1,1,1,0,0,0,0,1,1,1,1,1,1,1,1,1,1,1,0,1,0,1,1,1,0,1,1,0,1,0,1,1,0,1,0,1,1,1,0,1,1,1,1,0,0,0,1,1,0,0,1,1,1,0,0,1,1,1,1,0,0,0,1,1,1,1,1,0,0,0,0,1,1,1,1
L015_2016,0,0,1,1,1,1,1,0,1,1,0,0,0,0,0,1,1,1,1,1,0,1,1,1,1,0,1,1,1,1,1,1,0,1,1,1,1,0,0,1,1,1,1,1,1,0,0,1,1,1,1,1,1,1,0,1,1,1,1,1,1,0,1,0,1,1,1,0,1,1,1,1,1,0,1,0,1,0,0,0,1,1,0,0,0,1,1,1,0,1,1,1,0,0,0,0,1,0,1,1,1,0,0,0,1,1,1,1,0
L016_2016,1,0,1,1,1,1,0,1,1,1,0,0,0,1,1,1,1,1,1,1,1,1,1,1,1,0,1,1,1,1,1,1,0,0,1,1,0,0,0,1,1,1,1,1,1,0,0,0,1,1,1,1,1,1,0,1,1,1,1,1,1,0,1,0,1,1,1,1,1,1,0,1,1,0,1,0,1,0,0,0,1,1,0,0,1,1,1,1,0,1,1,0,0,0,1,0,1,0,1,1,1,1,0,0,0,0,1,1,1
L017_2016,1,0,1,1,1,1,1,1,1,1,1,1,1,1,0,1,1,1,1,1,1,1,1,1,1,0,0,1,1,0,0,1,0,1,1,1,1,0,0,0,1,1,1,1,1,1,1,1,0,1,1,1,0,1,1,0,1,1,1,1,1,0,1,0,1,1,1,1,1,1,0,1,1,0,1,1,1,0,0,0,0,1,0,0,0,1,1,1,0,1,1,1,1,1,1,0,1,0,1,1,1,1,0,0,0,1,1,1,1
L017_2017,1,0,1,1,0,1,1,1,1,1,1,0,1,1,0,0,1,1,1,1,1,1,1,0,0,0,0,1,1,0,1,1,0,1,1,0,0,0,0,1,1,1,0,1,1,1,0,0,1,1,1,0,0,1,0,0,1,0,1,0,1,0,1,0,1,0,0,0,1,1,1,1,1,1,1,1,1,1,0,1,0,0,1,1,0,0,1,1,1,0,1,1,1,0,0,0,0,1,0,1,1,1,0,0,0,0,1,0,1,1
L020_2016,1,0,1,0,0,1,1,1,1,1,0,0,1,0,0,1,0,0,0,1,1,0,0,1,1,0,0,1,1,0,1,1,1,0,1,1,1,0,0,1,1,1,1,1,1,1,1,1,1,1,1,1,1,1,0,1,1,0,1,1,1,0,0,0,1,1,1,1,1,1,0,1,1,0,1,0,1,0,0,0,1,1,0,0,1,1,1,0,0,1,1,1,1,1,1,0,1,1,1,1,1,0,0,1,0,1,1,1,1
L020_2017,1,0,1,0,0,1,0,1,0,0,0,0,0,1,1,1,0,1,0,1,1,0,0,0,1,0,0,1,1,1,1,1,1,0,0,1,1,0,0,1,1,1,1,1,0,1,1,1,1,0,1,1,0,1,0,0,1,1,1,1,1,0,1,0,1,0,1,1,1,1,0,1,1,1,1,1,1,0,0,0,1,1,0,0,1,1,1,0,0,1,1,1,1,1,1,1,1,1,1,1,1,0,0,1,0,1,1,1,1
L027_2016,0,0,1,1,1,1,0,1,1,1,0,0,1,1,0,1,1,0,0,1,1,1,1,1,1,0,1,1,1,0,1,1,0,1,1,1,0,0,0,1,1,1,1,1,1,1,0,1,1,1,1,1,1,1,0,1,1,1,1,1,1,0,1,0,1,1,0,1,1,1,1,0,1,1,1,0,1,0,0,0,1,0,0,0,1,1,1,0,0,1,1,1,1,1,1,1,0,1,1,0,1,1,1,0,0,0,1,0,1,1
L035_2016,1,0,1,1,1,1,1,1,1,1,0,0,1,0,0,1,1,0,0,1,1,1,0,1,1,0,0,1,1,0,1,0,0,1,1,1,1,0,0,0,1,1,1,1,1,1,1,1,0,1,0,1,1,1,0,1,0,0,1,1,1,0,0,0,1,1,1,1,0,1,0,0,1,0,1,0,1,0,0,0,0,0,0,0,0,1,1,0,0,1,1,1,1,0,0,0,1,0,1,1,1,0,0,0,1,1,0,1,0
L035_2017,1,0,1,1,1,1,1,1,1,0,1,0,0,1,0,1,0,1,0,1,1,1,1,0,1,0,1,1,1,1,1,1,0,1,0,1,1,0,0,1,1,1,1,1,0,1,1,1,1,1,1,1,1,1,1,0,0,1,1,1,0,1,0,1,0,1,1,1,1,0,1,0,1,1,1,1,0,1,0,0,0,1,1,0,0,1,1,1,0,0,1,1,1,0,1,1,1,1,0,1,1,1,0,0,1,0,1,1,1,1
L037_2016,1,0,1,1,1,1,0,0,1,1,0,1,1,1,0,1,1,1,1,1,0,1,1,1,1,0,1,1,1,0,0,1,1,1,1,1,1,0,1,1,1,1,1,1,0,1,1,1,0,1,1,1,1,1,0,0,0,1,0,1,1,0,1,1,1,1,1,1,1,1,1,0,1,1,0,1,1,1,0,0,0,0,1,0,0,1,0,1,0,0,1,1,1,1,1,1,1,1,1,0,0,1,1,0,0,0,0,1,1,1,1
L039_2016,1,1,1,1,1,1,0,1,1,1,1,0,1,0,1,1,1,0,0,1,1,0,0,1,1,0,0,0,1,0,1,0,0,1,1,1,0,0,0,0,1,1,1,0,1,1,1,1,0,1,1,0,1,1,0,1,1,0,1,1,1,0,0,0,1,1,0,1,1,1,1,0,1,0,1,0,1,0,0,0,1,1,0,1,0,0,1,0,0,0,1,1,1,1,1,1,1,1,0,0,1,1,1,1,0,0,0,1,0,1,0
L039_2017,1,0,1,0,0,1,0,1,1,0,0,0,1,1,0,1,0,1,1,1,1,1,1,1,0,0,0,1,1,1,1,0,0,0,1,1,1,0,0,0,1,1,1,1,1,0,1,1,1,1,1,1,1,1,1,1,0,1,1,1,0,0,1,0,0,0,0,1,1,1,0,1,1,0,1,1,1,0,1,0,0,0,0,1,0,0,1,0,1,0,0,1,1,1,0,0,0,0,1,1,1,1,1,1,1,0,0,1,1,0,1,1
L043_2016,0,0,1,0,0,1,1,0,1,0,1,0,1,1,0,1,1,0,1,1,1,1,0,1,1,0,1,1,1,1,1,1,1,0,1,1,1,0,0,0,1,1,1,1,1,1,1,1,1,1,1,1,0,0,1,0,1,1,1,1,1,1,0,1,0,1,1,1,0,1,1,1,1,1,0,1,0,1,0,0,0,0,1,0,0,1,1,1,1,0,1,1,1,1,1,1,1,0,1,0,0,1,1,1,0,0,0,1,1,1,1
L043_2017,1,0,1,0,0,1,1,0,1,1,1,0,1,1,1,0,0,1,0,1,1,1,1,0,1,0,0,1,1,0,1,1,0,1,0,1,0,0,0,1,1,1,0,0,0,0,0,0,0,0,0,1,0,1,0,0,1,0,1,1,1,0,1,0,1,0,1,0,1,1,1,1,1,1,1,1,1,1,0,1,0,1,1,1,0,1,1,1,1,0,1,1,1,1,1,1,1,1,1,1,0,0,1,1,1,0,1,1,1,0,1,1
L048_2016,1,0,1,0,0,1,0,1,1,1,0,1,1,1,0,1,1,0,0,1,1,1,1,1,1,0,1,1,1,0,1,1,0,1,1,1,1,0,0,1,1,1,1,1,1,1,1,1,1,1,1,1,1,1,1,0,1,1,1,1,1,1,0,1,0,1,1,0,1,1,1,1,1,0,0,1,0,1,0,0,0,0,1,0,0,1,1,1,0,0,1,1,0,1,0,0,0,1,1,1,1,1,0,0,0,0,1,1,1,1
L056_2016,1,1,1,1,1,1,1,1,1,1,1,1,0,1,0,1,0,1,1,1,1,1,1,1,0,1,0,0,1,1,1,0,0,0,0,0,0,1,0,0,0,1,0,1,0,0,0,0,1,0,0,0,1,0,1,0,1,1,1,1,1,1,0,0,0,1,1,1,0,0,1,1,1,0,0,1,1,1,0,0,0,0,1,0,0,0,0,1,1,0,1,1,0,1,1,1,1,1,0,1,1,0,0,1,1,0,1,1,1,1
L060_2016,1,0,1,1,1,1,0,0,1,0,1,0,0,0,0,1,1,1,0,1,1,1,1,0,1,0,1,1,1,1,1,1,0,0,0,1,1,0,1,1,1,1,0,0,1,1,0,1,1,0,0,1,0,1,0,0,0,0,1,1,1,0,1,0,1,0,1,1,0,1,0,1,1,1,1,1,1,0,0,0,1,1,1,0,1,1,1,0,0,1,1,1,1,1,1,1,1,0,1,1,1,1,1,0,1,0,1,1,1,1
L060_2017,1,0,1,0,1,1,0,0,1,0,1,0,1,0,1,1,1,1,1,1,1,1,1,1,1,1,0,1,1,1,0,1,1,0,1,0,1,1,0,1,1,1,1,1,1,0,0,1,1,0,0,1,1,1,1,0,1,1,1,0,0,1,0,0,0,1,1,1,0,1,1,1,1,1,1,1,1,1,0,0,0,1,1,0,0,1,1,1,0,0,1,1,1,1,1,1,0,0,1,1,1,1,1,1,1,0,1,0,1,1,1,1
L087_2016,1,0,1,0,1,1,0,1,1,0,1,0,0,0,0,1,0,1,1,1,1,1,0,1,0,0,0,1,1,0,0,0,0,0,0,1,1,0,0,1,1,1,1,1,0,1,1,1,1,1,1,1,0,1,0,0,1,1,0,0,1,0,1,0,1,1,1,1,1,1,0,1,1,1,1,1,0,0,0,0,0,1,0,0,1,1,1,1,0,1,1,1,0,0,0,0,0,0,0,1,1,0,0,0,0,1,0,1,1
L087_2017,1,1,1,1,0,1,1,1,1,0,1,0,0,1,1,1,0,1,1,1,1,1,1,1,1,1,1,0,0,1,1,0,1,1,0,1,0,1,0,0,1,0,1,0,1,1,1,0,1,1,1,0,1,1,1,1,0,0,1,1,1,0,1,0,1,0,1,1,1,1,0,1,1,1,1,1,1,0,1,0,0,0,1,1,0,0,1,1,1,0,0,1,1,1,1,1,1,1,1,1,0,1,1,0,0,0,0,1,0,1,1
L090_2016,1,0,1,0,1,1,0,1,1,0,0,0,0,0,1,1,1,1,1,1,1,1,1,1,1,1,0,1,1,1,1,1,1,0,0,0,1,1,0,0,0,1,1,1,1,0,0,1,1,1,1,1,1,0,1,0,0,0,0,1,0,1,0,1,0,1,0,1,1,1,1,0,1,1,1,1,1,1,0,0,0,0,0,0,0,1,1,1,0,0,1,1,1,0,0,0,0,1,0,0,1,1,0,0,1,0,1,0,1,1
L090_2017,1,1,1,0,0,1,1,1,1,0,1,0,1,1,0,0,1,1,1,1,0,1,1,0,1,0,1,1,1,0,1,1,0,0,1,1,0,0,0,1,1,1,1,1,0,0,1,1,1,1,0,1,0,1,0,1,1,1,1,0,1,0,1,0,1,1,1,1,1,1,1,1,1,0,1,1,1,0,0,0,1,1,0,0,1,1,1,0,0,1,1,1,0,0,0,1,1,1,1,1,1,1,0,0,1,0,1,1,1,1
L092_2016,1,0,1,0,1,1,1,1,1,1,0,1,0,1,0,1,1,0,1,1,1,1,1,1,1,1,1,0,0,1,1,0,0,1,1,1,1,1,1,0,0,1,1,1,1,1,1,1,1,1,0,0,1,1,1,0,1,0,1,0,0,1,0,1,0,0,0,1,1,0,1,1,1,1,1,1,1,1,0,1,0,1,0,1,0,0,0,1,1,1,1,0,1,1,1,1,1,1,1,0,1,0,0,1,1,0,0,0,0,1,0,1,0
L097_2016,1,0,1,1,1,1,0,1,1,1,0,1,1,1,1,0,0,1,1,1,1,1,1,1,1,1,0,0,1,0,0,1,0,1,1,1,1,1,0,0,1,1,1,1,1,1,0,1,1,1,1,1,0,0,1,0,1,0,0,0,0,1,0,0,0,1,1,0,1,1,1,1,0,1,0,1,0,1,0,0,0,1,0,0,1,0,1,1,1,0,1,1,1,0,0,0,0,0,0,0,0,1,0,0,0,0,0,0,1,0,1,0
L099_2016,1,0,1,0,1,1,0,1,1,1,1,1,1,0,1,1,1,1,1,1,1,1,1,1,1,1,0,0,0,1,0,1,0,0,1,1,1,1,0,1,0,1,1,1,1,0,1,0,1,1,1,1,1,1,1,0,1,1,0,1,1,1,0,0,0,1,0,1,0,1,1,1,0,1,0,1,0,1,0,1,0,1,1,0,0,1,1,1,1,0,1,1,1,1,1,1,1,0,0,0,0,1,1,0,1,0,0,1,1,1,1
L102_2016,1,0,1,0,1,0,0,1,1,1,1,1,0,1,0,1,1,1,1,1,1,1,1,0,1,1,0,0,1,0,0,0,1,0,1,1,1,1,0,0,1,1,1,1,1,0,0,1,0,0,1,0,1,0,1,0,1,1,0,1,1,1,0,1,0,1,1,1,1,1,1,1,0,1,1,0,1,0,1,0,0,0,0,1,0,0,0,1,1,0,0,1,1,1,1,1,1,0,0,1,0,1,1,1,0,1,0,1,1,0,1,0
L103_2016,0,1,0,0,1,0,0,1,1,1,1,1,0,1,1,1,0,1,1,1,1,1,1,1,1,1,0,0,1,1,0,0,0,0,1,1,0,1,0,0,1,1,1,1,1,0,1,1,1,0,1,1,1,0,1,0,1,1,0,0,0,1,0,1,0,1,0,1,0,1,1,1,0,1,0,1,0,1,0,1,0,1,1,0,0,0,0,1,1,0,1,1,1,1,1,1,0,0,0,0,0,0,1,0,1,0,0,1,1,0,1,0
